# Anesthetic Management for Patients with Placenta Accreta Spectrum: A Scoping Review

**DOI:** 10.3390/jcm14134738

**Published:** 2025-07-04

**Authors:** Tomasz Jasinski, Aleksander Remesz, Rafal Resko, Aleksandra Budynko, Katarzyna Majdylo

**Affiliations:** 1 Department of Anesthesiology and Intensive Therapy, Medical University of Gdansk, 80-210 Gdansk, Poland; 2 Department of Anesthesiology and Intensive Therapy, University Clinical Centre, 80-210 Gdansk, Poland

**Keywords:** anesthesia, cesarean delivery, interventional radiology, obstetric anesthesia, placenta accreta spectrum, placenta diseases

## Abstract

**Background**: Placenta accreta spectrum (PAS) is a condition in which villous tissue pathologically adheres to or invades the uterine wall, which may result in massive bleeding with substantial maternal morbidity and mortality. Despite the constantly increasing prevalence of this condition, an optimal anesthetic management method for this condition has not been fully established. A scoping review of the literature was performed to evaluate current anesthetic management strategies for PAS. **Methods**: This review was conducted using the Joanna Briggs Institute (JBI) methodology for scoping reviews. A search of five databases (MEDLINE, EMBASE, CINAHL Complete, Scopus, and Web of Science) was conducted to identify articles containing data on seven prespecified aspects of PAS anesthetic management. Studies that described the management of miscarriage and abortion procedures were excluded. **Results**: One hundred thirty articles encompassing numerous approaches to PAS management were included in the final review. Data were mostly extracted from case reports (n = 56) and observational studies (n = 64). The most commonly adopted strategy (n = 62) was the creation of a multidisciplinary team comprising various specialists, including anesthesiologists. **Conclusions**: Due to the limited number of interventional studies, the most effective methods of anesthetic management for this condition could not be fully established. However, the safe and effective anesthetic management of PAS patients is feasible. Future research in this field should focus on resolving the identified knowledge gaps and increasing the quality of published studies.

## 1. Introduction

The term placenta accreta spectrum (PAS) describes a complication of pregnancy in which villous tissue pathologically adheres to or invades the uterine wall, which eventually causes the failure of placental separation during delivery [1]. Current data demonstrate that the incidence of this disorder reached 1 in 272 patients in 2016 in comparison to the initial incidence of 1 in 30,000 patients reported in the 1930s [2,3]. The main reason for this change includes the persistently increasing rate of cesarean delivery (CD), which is the most important risk factor for invasive placentation and currently has an incidence of 32.1% in the USA [4]. When placenta previa is also present, the risk of developing PAS may reach 60% after the third CD [5].

The presence of PAS may lead to adverse outcomes, including stillbirth, neonatal death, hemorrhage, emergency hysterectomy, massive transfusions, abdominal organ injury, and intensive care unit admission due to maternal morbidity [6]. Nieto-Calvache et al. analyzed 52 maternal deaths related to PAS and revealed that all cases may have been preventable if proper interventions were implemented [7]. This condition can be managed by a myriad of strategies, including cesarean hysterectomy (which represents a radical [or extirpative] approach) and various uterine-sparing techniques (which represent conservative approaches) [8,9]. Moreover, effective treatment of PAS extends beyond surgical options; according to current guidelines, a multidisciplinary approach is recommended for the management of such complicated pregnancies [1]. PAS referral centers exist in which experienced teams of specialists manage PAS in a systematic manner.

Anesthesiologists play an important role in multidisciplinary PAS teams and are responsible for more than the simple administration of proper anesthetics. These personnel are suited to be actively involved in planning the delivery, assessing the patient, and optimizing the patient’s condition, as well as selecting the transfusion strategy and planning the patient’s postsurgical disposition. The presence of an experienced anesthesiologist in complex obstetrical cases and proper anesthetic management may improve the outcomes of PAS patients. Such attitudes can be identified in the checklist and system preparedness bundle for placenta accreta spectrum created by the Society for Maternal-Fetal Medicine [10]. However, a literature review by Enste revealed that the optimal anesthetic management method for PAS patients is unclear, with only low-quality or nonspecific evidence available [11]. This observation was confirmed in a review by Warrick and colleagues, in which five knowledge gaps concerning major aspects of perioperative management were identified [12].

Studies and evidence-based recommendations to resolve these gaps can facilitate the creation and refinement of standardized protocols for anesthetic management in PAS patients. The adoption of such protocols by multidisciplinary teams in referral centers may enhance obstetric anesthesia education and training and optimize maternal and newborn outcomes after PAS surgery.

Considering the abovementioned factors, we reviewed the methods of anesthetic management used in PAS cesarean delivery and reported outcomes. Before a formal review protocol was created, a preliminary search of MEDLINE (PubMed) was conducted; this did not reveal any guidelines or consensus statements on this topic and indicated that most data would likely be derived from observational rather than experimental studies, which would be a serious limitation for conducting a systematic review. Moreover, we found that valuable data are commonly available in reports on PAS management that did not directly focus on the anesthetic aspects of care. Therefore, our objectives were to identify published studies containing information on the anesthetic management of PAS, summarize the findings, and identify areas where the available data are inconclusive. The results may improve the quality of future studies and ultimately influence practice management. These objectives resemble indications for the creation of a scoping review, as described by Tricco et al. [13]. In conclusion, despite its limitation of being descriptive rather than interpretative in nature, this approach was considered by the study team as the most appropriate for this research.

### Objective

The aim of this review was to verify the available data by addressing two key questions. First, it sought to understand the current practices of anesthetic management in patients experiencing labor with suspected PAS. Second, it examined the outcome measures reported in studies focused on the anesthetic management of patients with suspected or confirmed PAS.

## 2. Materials and Methods

This review was conducted in compliance with the JBI methodology for scoping reviews and in accordance with an a priori protocol [14,15]. The protocol was not registered, as scoping reviews cannot be registered via the PROSPERO database. The manuscript was prepared in accordance with the Preferred Reporting Items for Systematic Reviews and Meta-Analyses extension for Scoping Reviews (PRISMA-ScR) [16].

### 2.1. Eligibility Criteria

#### 2.1.1. Participants

Following an a priori protocol, studies that included pregnant patients who underwent cesarean delivery with suspected or confirmed PAS were selected for this review.

#### 2.1.2. Concept

This scoping review included studies that presented data on methods for managing anesthesia in patients diagnosed with PAS. In order to clarify the term “anesthetic management” to facilitate screening, the extracted results were interpreted according to seven issues of interest:

1. The structure and setting of anesthetic services;
2. The anesthetic technique and pharmacology;
3. Monitoring;
4. Hemodynamic management and fluid therapy;
5. Blood product management and hemostasis;
6. Postoperative care;
7. Maternal and newborn outcomes.

For each of these topics, detailed research questions were formulated (Table 1). Records that contained detailed data on at least one of the topics (I–VI) were considered to have met the inclusion criteria. Records with data on issues V and VI were considered to be eligible only if the data were provided in the context of anesthesia. Such measures prevented the overextraction of data not directly associated with the purpose of this review. Regarding issue VII, our goal was to assess the most clinically valuable, patient-centered outcomes instead of non-selectively assessing the reported outcome measures. Such an approach would verify whether the authors who conducted the included studies used outcomes that helped to optimize the postpartum recovery of PAS patients, which may be strongly influenced by anesthetic management. For this purpose, a list of essential outcomes was created by the study team using the core outcome set for enhanced recovery after cesarean delivery by Sultan and colleagues [17].

#### 2.1.3. Context

This scoping review included studies involving both scheduled and emergency patients who had an intraoperative or postoperative diagnosis of PAS or were suspected of having PAS prior to delivery. Only data from studies of patients who underwent surgical deliveries were examined. Studies in which anesthesia was managed for patients who underwent deliveries for miscarriages and abortions associated with abnormal placentation were not considered to be eligible.

### 2.2. Information Sources and Search Strategy

Both experimental and quasiexperimental studies were considered for inclusion in this scoping review, including the following:

- Randomized controlled trials (RCTs), nonrandomized controlled trials,
- and before-and-after studies;
- Analytical and descriptive observational studies (including prospective and retrospective cohort studies, case–control studies, case series, and individual case reports).

To increase the comprehensiveness of the extracted data, the study team also included conference abstracts, correspondence papers, or letters to the editor if they contained adequate data on anesthetic management. Text and opinion papers were not considered for inclusion in this scoping review.

The search strategy was designed to identify both published and unpublished studies. In the first step, an initial limited search of MEDLINE (PubMed) was performed to identify relevant articles on the topic. The words that were extracted from the titles and abstracts of relevant papers and the index terms describing the articles were analyzed to create a full search strategy; in the next stage, the terms were adapted to screen for articles in the remaining databases. The full search was performed in March 2023 and subsequently updated in November 2024. Only studies published in English were included because all of the authors of this review are fluent in this language. The search was restricted to studies published after 1980 because the research team agreed that data from studies published before that date may not reflect current anesthesiology practices. This also corresponds with the fact that these data indicate the beginning of the period during which the incidence of PAS began to continuously increase [149]. The inclusion and exclusion criteria are summarized in Supplementary Section S3.

The following databases were searched for relevant studies: MEDLINE (PubMed), EMBASE (Elsevier), CINAHL Complete (EBSCO), Scopus (Elsevier), and Web of Science (Clarivate). Google Scholar and Advanced Google were also searched to identify potential gray literature sources or studies that were not identified during the database search. Based on the results of the database search, the study team decided not to perform further gray literature searches. The full search strategy and results are presented in Supplementary Section S1.

### 2.3. Study Selection Methods

**Figure 1:**
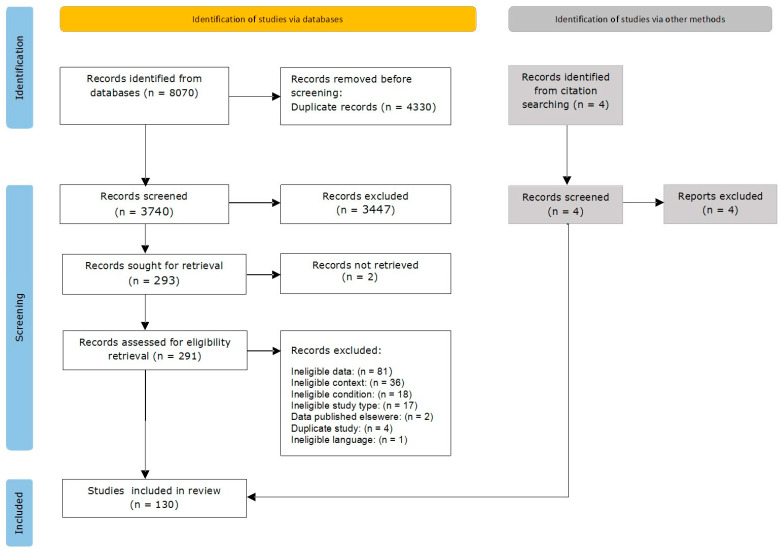
PRISMA flow diagram of the search and study selection process.

Following the search, all of the identified studies were collated and uploaded to Zotero Reference Manager version 6.0.20/2023 (Corporation for Digital Scholarship, Vienna, VA, USA), after which duplicate data were removed. Two independent reviewers subsequently screened the titles and abstracts according to the inclusion criteria. The full texts of the articles that were deemed to be potentially relevant were retrieved, and citations were imported into the JBI System for the Unified Management, Assessment and Review of Information (JBI SUMARI) (JBI, Adelaide, Australia). The full texts of the selected sources were retrieved and assessed to determine whether they met the inclusion criteria; each record was assessed by two reviewers. Studies that did not meet the inclusion criteria were excluded. The reasons for exclusion are provided in Supplementary Section S2. Any disagreements between the reviewers were resolved via discussion with all of the team members. The reference lists of the included studies were manually searched for relevant citations, which were subsequently assessed for relevance based on their titles and abstracts and included in the full-text screening stage if they met the inclusion criteria. The search results are provided in a PRISMA flow diagram shown in Figure 1.

### 2.4. Data Extraction

Study details (author, year of publication, country of origin, study design, population characteristics, and diagnosis) and data related to specific aspects of anesthetic management were extracted using a data extraction tool developed by the research team during the protocol development stage [15]. The data extracted from each included study were critically reviewed by two independent reviewers and subsequently verified by another team member to ensure the accuracy of the process and resolve any discrepancies. The review protocol allowed for possible modifications of the data extraction tool (if necessary).

### 2.5. Deviations from the Original Protocol

After the analysis of the preliminary search results, we decided to extend the original protocol and clarify the general characteristics of the research questions, as described in the “Concept” subsection”. Additionally, we decided to provide a critical appraisal of the studies included in the final review. Such an analysis is not considered mandatory for a scoping review and was initially not planned. However, the study team concluded that this analysis would increase the quality and facilitate the interpretation of the review findings.

### 2.6. Critical Appraisal of the Included Studies

A critical appraisal of the included studies was performed using the JBI Critical Appraisal Checklists and a checklist for conference abstracts, letters to the editor, and correspondence papers, which was created by the authors [15,150].

### 2.7. Data Synthesis

The results of this scoping review are presented in a narrative summary. Considering the issues of interest, figures and tables were deemed to provide the most comprehensible presentation of the data. A narrative summary clarifying the data and describing their relationship to the review objectives is provided.

## 3. Results

### 3.1. Study Selection

A total of 8070 studies were identified by searching databases and additional sources (Figure 1). After 4330 duplicates were removed, the remaining 3740 titles and abstracts were screened according to the inclusion criteria. As a result, 3447 records were excluded, and 293 were chosen for a full-text analysis. A total of 161 studies were excluded at this stage. The full texts of 2 studies could not be retrieved; therefore, 130 studies were included in this review. No additional records were identified by manually searching the reference lists of the included sources.

### 3.2. Study Characteristics

This scoping review included studies that were published between 1987 and 2024, most of which were published during the last 10 years. The studies were conducted in 28 countries across 6 continents. The majority of these studies originated from Asia and North America (Table 2). The most prevalent study types included case reports, case series, cohort studies, and quasiexperimental studies. Three randomized clinical trials were identified. Data were also extracted from 11 conference abstracts, 3 correspondence papers, 3 letters to the editor, and 1 case study. The detailed characteristics of the included studies are presented in Supplementary Section S3.

### 3.3. Critical Appraisal of the Included Studies

The results of the critical appraisal of the included studies are presented in Supplementary Section S3. Answers to questions on the appropriate checklists are provided for each study.

### 3.4. Synthesis of Results

#### 3.4.1. General Data

The distributions of the three main types of PAS among the included studies are presented in Supplementary Section S3. The presence of risk factors (previous CD, other uses of uterine instrumentation, or placenta previa) for the development of PAS was mentioned in 109 of the included studies. Extirpative and conservative approaches were reported in 107 and 50 studies, respectively. Data on emergency procedures due to various factors (such as bleeding or established labor) or an unexpected intraoperative diagnosis of PAS were presented in 48 articles. Detailed data regarding the type of procedure and any adjuvant procedures (such as interventional radiology [IR], REBOA catheter, and cystoscopy) are available in Supplementary Section S3.

PAS procedures are typically performed in operating rooms. Two studies emphasized the use of a general operating room instead of an obstetric operating room because of the availability of additional resources and staff qualifications [30,83]. Morland et al. reported the use of a cardiothoracic theater due to a patient’s comorbidities [84]. IR procedures typically required the use of hybrid rooms or radiological suites, with subsequent patient transfers between facilities for the remaining portion of PAS surgery in 12 and 24 studies, respectively. Detailed data on the PAS and IR procedures are presented in Supplementary Section S3.

#### 3.4.2. Structure of Anesthetic Services

A formal multidisciplinary team (MDT) was identified in 62 studies (Table 1). However, only four studies provided a detailed description of a multidisciplinary procedure protocol (Supplementary Section S3) [21,31,32,33]. The composition of the MDTs varied. A full list of the identified MDT members is presented in Supplementary Section S3. In the remaining studies, the presence of an MDT may not have been mentioned, or other potential alternative personnel structures may have been involved.

Anesthesia providers were mentioned as being members of the MDT in 40 of the studies (Supplementary Section S3). In a study by Russo et al., an anesthesiologist served as the coordinator [85]. Three studies provided details about the complete composition of the anesthesia team and the roles of the specific members [86,87,88]. In two of the identified articles, anesthetics were administered by two anesthesiologists [34,52]. The involvement of a consultant and a senior anesthesiologist (as well as an obstetric anesthesiologist) was reported in four and five studies, respectively [30,31,35,36,53,54,89,90,91]. Lopez-Erazo et al. reported on dedicated anesthesiologists on an MDT and additionally consulted cases of PAS patients anesthetized by individuals who were not members of the MDT [21].

Regarding a risk stratification system including multidisciplinary planning, Weiniger et al. reported on a system of grading patients as a “high suspicion” or “low suspicion” of having PAS; moreover, Nieto-Calvache reported on bleeding risk assessments to stratify surgical strategies and minimize blood loss [22,37,151,152]. The exact influence of these factors on the composition of the MDT and the location of the surgery were not provided.

#### 3.4.3. Anesthetic Technique and Drugs

**Figure 2:**
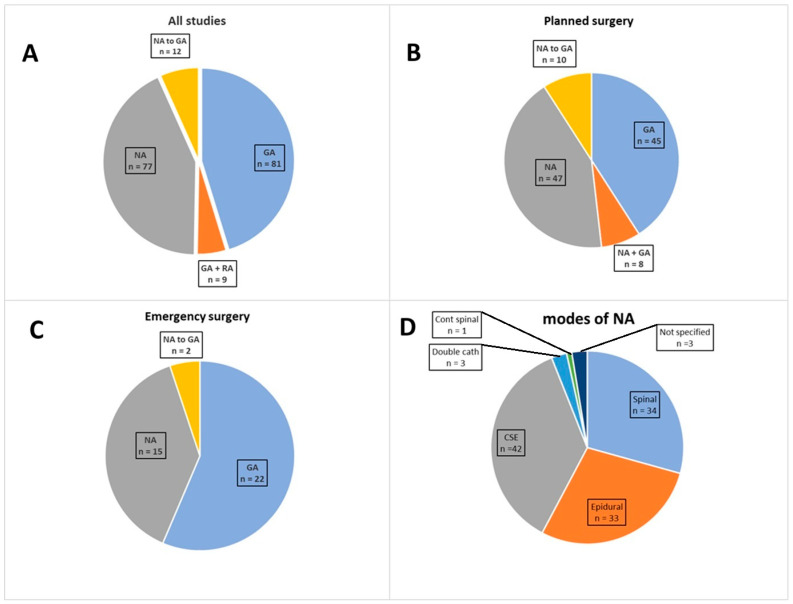
Initial anesthetic approach (**A**). The initial mode of anesthesia in planned surgeries (**B**) and emergency surgeries (**C**) and the methods of regional anesthesia (**D**) identified in the included studies (GA, general anesthesia; RA, regional anesthesia; GA + RA, the predelivery of general and regional anesthesia; NA to GA, planned conversion from neuraxial to neuraxial anesthesia; CSE, combined spinal and epidural anesthesia).

The modes of anesthesia used during cesarean delivery (CD), as well as their distributions among planned and emergency deliveries and the utilized methods of neuraxial anesthesia (NA), are presented in Figure 2. The identified double-catheter neuraxial techniques included lumbar continuous spinal and epidural (CSE) with thoracic epidural anesthesia and lumbar epidural with continuous spinal anesthesia techniques [31,38,55]. Studies in which primary NA was utilized reported typical conversion rates ranging from 20–30% to 66% [39,40,56]. The identified reasons for selecting general anesthesia (GA) or NA and for unplanned conversion from NA to GA with the timing relative to surgery are presented in Table 3. Seven studies included comparisons of outcomes between NA, GA, or NA and subsequent conversion to GA in PAS surgery [18,33,39,41,42,43,44,92]. There was no considerable difference observed between the GA and NA techniques for patients who received planned GA, planned NA, or planned conversion from NA to GA.

Forty studies reported the use of various methods for managing NA during IR PAS procedures (Supplementary Section S3). The avoidance of NA in IR was attributed to either the risk associated with concomitant heparin administration, the anticipated risk of IR intraarterial catheter displacement, or both [23,57,58,93,94]. Two cases of such displacement were identified, one of which resulted in severe adverse effects, as reported by Thon et al. [59,80]. In this case, patient movement was suspected, with the suggestion that NA should precede IR procedures. Among the identified studies, this was the preferred approach (Supplementary Section S3).

For GA, in the majority of studies, intravenous induction with volatile maintenance was performed. In seven studies, the use of total intravenous anesthesia (TIVA) was reported [24,54,56,60,93,95,96]. Konishi et al. described the transition from volatile anesthetics to TIVA in order to facilitate uterine relaxation and uterine contractility assessments using volatile agents and propofol, respectively [95]. Considering the intraoperative conversion of NA to GA, none of the studies mentioned any recommendations for the anesthetic induction technique.

#### 3.4.4. Periprocedural Monitoring

Detailed information on standard monitoring methods (including NIBP, ECG, and pulse oximetry) was obtained from 44 studies (Table 1). Detailed data on the use of other types of noninvasive monitoring are available in Supplementary Section S3.

The main type of invasive monitoring involved invasive blood pressure monitoring. Arterial cannulation and its use for blood pressure measurement were mentioned in 99 studies (Supplementary Section S3). Central venous pressure monitoring was reported in nine studies [24,40,45,61,97,98,99].

With respect to advanced minimally invasive or noninvasive hemodynamic techniques, transesophageal echocardiography and transesophageal Doppler were utilized (Supplementary Section S3). The intraoperative use of Swan Ganz catheters was not reported in any of the included studies.

#### 3.4.5. Hemodynamic Management and Fluid Therapy

The type or volume of infused crystalloids and colloids was reported in 56 and 30 of the studies, respectively (Supplementary Section S3). No data on the reason for particular fluid choice were found. One study assessed the issue of the fluid resuscitation strategy [35].

The most commonly used peripheral venous access scheme (if reported) involved the placement of two large-bore cannulas, which was mentioned in 37 studies (Supplementary Section S3). Central venous cannulation was reported in 59 studies (Supplementary Section S3). No risk stratification systems that defined appropriate venous access were identified, although restrictions of its use to specific cases requiring vasopressor administration, large volumes of fluid administration, or monitoring were presented in three studies [21,40,62].

The use of vasoactive drugs was reported in 44 studies—the remaining studies did not mention such data (Table 1). The most commonly used methods were phenylephrine and ephedrine (Supplementary Section S3). No reasons for the choice of particular agent or comparative data of their use were found. Seven studies reported blood pressure target values or thresholds for vasopressor administration [63,86,87,100,101,102,103,104].

#### 3.4.6. Blood Product Management and Hemostasis

Nieto-Calvache et al. assessed the impact of MDT management on transfusion practices and consequently elucidated the role of anesthesiologists in implementing an interdisciplinary approach to managing bleeding risks [22]. Twenty-two studies reported the use of a formal massive transfusion protocol (MTP) (Table 1). However, only one study by Biji et al. provided detailed information and a graphical representation of the implemented MTP, whereas Humphrey et al. described the use of a protocol adopted from another study; its details are available in the original article [61,105,153].

The reported amount of blood loss varied, with 47 liters observed in a study by Kume and colleagues [64]. However, only 18 studies provided methods for assessing blood loss (Table 1). In one randomized controlled trial, the Mercurali and Nadler formula was used for blood loss calculations [19].

Regarding the transfusion strategy, 11 studies provided target values or thresholds for blood product administration, and 6 studies used a fixed blood product ratio. The use of thromboelastography (TEG) for perioperative coagulopathy assessment was recorded in six studies; however, only Alvarado-Ramos et al. provided defined TEG values that were used as a trigger for plasma transfusion [52]. Among the utilized hemostatic agents, the most commonly used were cryoprecipitate and tranexamic acid. In 12 out of 36 studies reporting intraoperative blood salvage, amniotic fluid risk (AFE) was considered, and precautions (such as the use of filters, a delayed initiation of blood salvage, or AFE outfiltering) were mentioned [22,64,65,93,105,106,107,108,109,110,111,112]. Detailed data on blood product management are available in Supplementary Section S3.

#### 3.4.7. Postoperative Care

Seventy studies reported the postoperative admission of PAS patients to the intensive care unit (ICU), with the most common indication being the need for mechanical ventilation or vasoactive support (Table 1, Supplementary Section S3). Postoperative pain management was the main objective of the study by Panjeton et al. [44]. However, such data were also observed in other studies (Table 1). Pain assessment via the visual analog scale (VAS) was reported by both Plakothina and Traversa et al. [18,113]. Epidural analgesia and patient-controlled analgesia were the most commonly reported methods of postoperative pain management (Table 1). Peripheral blocks (exclusively via transversus abdominis plane blocks) were used for postoperative analgesia in six studies [25,95,103,114,115,116]. In all of the patients, the single-shot technique was used after the surgeries were completed. In addition to the study by Valentine et al., peripheral block utilization was associated with general anesthesia [114]. Only Alford et al. described its use as an emergency treatment for unrelieved pain [115].

#### 3.4.8. Maternal and Newborn Outcomes

The most commonly reported maternal and neonatal outcomes included the length of hospital stay and the Apgar scale score, respectively. Full data on the incidence of outcomes from the aforementioned list of essential outcomes are presented in Table 4 and Supplementary Section S3. With respect to the other identified outcome measures, six studies reported maternal mortality, and eight reported neonatal mortality [19,26,32,40,42,46,66,67,68,80,92,117]. Five studies contained information about fetal loss [26,33,47,48,69]. The umbilical pH was identified in five studies as an outcome reflecting newborn conditions [19,27,44,55,70]. The development of respiratory distress, the duration of mechanical ventilation, and the neonatal length of hospital stay were reported in four studies each [19,32,44,64,67,70,80,93,118].

## 4. Discussion

This scoping review identified more than 100 studies containing data on the methods used to manage anesthesia in PAS delivery over the past 40 years. An analysis of the publication year revealed a gradual increase in interest in the topic of PAS management, as the majority of studies were published after 2010. Most identified studies were observational—there was a considerable paucity of interventional trials.

In summary, PAS is typically managed by MDTs, but a profound analysis of anesthetic management in the included studies showed that the approach to different issues, such as the optimal method of anesthesia and the influence of surgical approach (for example, the utilization of IR) on its choice, varies considerably. Data on other aspects such as the optimal fluid therapy, hemodynamic management, and postoperative care were sparse. Reporting on other aspects such as details on blood management or outcomes was inconsistent.

The management of PAS by an MDT involves adherence to available recommendations. The optimal composition of the MDT and anesthesiology team may depend on local regulations; however, the presence of an experienced anesthesiologist regarding PAS management seems to be highly recommended. For example, in a study by Lopez-Erazo et al., this approach resulted in the increased use of NA [21].

The optimal method of anesthesia for PAS delivery requires further investigation. A systematic review by Hung et al. suggested that NA is an evidence-based management method; however, an analysis of the studies included in our review revealed that various modes of anesthesia and pharmacological compositions are effective if properly adopted in management protocols [154]. Considering the reported reasons for the choice of GA or NA (Table 2), the risks of hemorrhage and hemodynamic instability are crucial factors justifying the choice of GA for the PAS procedure. As a result, greater blood loss and transfusion requirements during GA may be biased by patient selection, wherein patients with a greater risk of hemodynamic instability and complex cases may preferentially receive GA. Ioscovich et al. concluded that the obstetric characteristics of the patient (rather than the anesthetic technique) determine blood loss in abnormal placentation [54]. However, propensity score matching utilized by Liu et al. to compare GA and NA demonstrated consistent benefits for NA in terms of blood loss, the length of surgery, and the length of hospital stay [42]. Conversely, the increased risk of hemodynamic instability during the unplanned conversion of NA to GA cannot be unambiguously confirmed. A study by Markley et al. concluded that such events may cause hemodynamic compromise; however, the use of GA as the sole method for abnormal placentation is also associated with potential maternal hypotension and the need for vasopressor support [39]. These findings further emphasize the role of proper planning, MDT involvement, and the rarely reported use of risk stratification systems when choosing a suitable mode of anesthesia for PAS surgery. The findings also facilitate the consideration of the two main identified reasons for the choice of NA: parturient preferences and the risk of fetal exposure to anesthesia. As demonstrated in a survey-based study by Bartels, the consideration of parturient preferences is a complex issue requiring an interplay between patients’ desires for involvement in decision-making and an understanding of their actual expectations by healthcare providers [155,156]. Postoperative satisfaction and the risk of potential childbirth-related traumatization, which may increase during invasive surgical modes of birth (such as PAS delivery), seem to be particularly important [157,158]. The significance of the latter factor should be investigated in further studies because, among the identified studies, only a case series by Akinaga and colleagues focused on this issue, and larger studies are needed to provide conclusive evidence [70].

The influence of IR procedures on PAS anesthetic management is mainly related to problems with the IR location and the optimal timing of NA and IR. Reported cases of catheter malpositioning have demonstrated a questionable relationship with anesthesia [59,80]. Surprisingly, only two studies provided information on the heparin dose used during IR procedures [112,119]. According to the personal experiences of the authors of this review, administered doses of unfractionated heparin can reach a level at which neuraxial blocks are contraindicated, according to current guidelines [159]. Thus, the reporting of the details of anticoagulant treatments and the associated timing of neuraxial anesthesia is advisable.

Standard noninvasive monitoring with additional invasive blood pressure measurements during PAS surgeries was typically reported. However, the risks of large fluid shifts and hemodynamic instability resulted in practical decisions to utilize minimally invasive or noninvasive hemodynamic measurements. Unfortunately, the results of the studies assessing their use may be difficult to apply in practice, as they were only conducted in specific patients [84,106]. The data on related topics of fluid therapy and hemodynamic support are also inconclusive, as only Zhou et al. have assessed the feasibility of restrictive fluid resuscitation strategies [35]. Among the eight studies mentioning goal-directed hemodynamic management, only Loreto et al. have provided sufficient data suggesting its clinically useful benefits [20,25,27,52,84,91,106,120]. Other identified studies have provided only pure volumes of infused fluids and basic data on vessel cannulation and vasopressor use. Data from trials and metanalyses of general obstetric populations suggest that the adopted fluid resuscitation strategy and the choice of vasopressor are of clinical significance [148,160]. Thus, providing such details in observational studies and conducting studies to comparatively assess various approaches would positively impact the research and practice of anesthetic management in this group of patients.

Blood loss and the number of transfused blood products were almost universally reported in the included studies. Regarding blood management, Zabida et al. revealed that unprotocolized transfusion practices based on clinical judgment resulted in over-transfusion; thus, formal MTPs in PAS referral centers are highly advisable [49]. Unfortunately, the studies provided limited data on their details, leading to issues in comparing the various MTPs. Considering blood loss assessments, the 2019 ACOG Committee Opinion indicated the use of quantitative methods [161]. Such calculations were identified in a small number of included studies. The use of these calculations should be integrated into local protocols used by MDTs, and this information should be included in published studies. Data on adopted blood transfusion thresholds, blood product ratios, and details of point-of-care TEG/ROTEM management should also be provided.

The identified postoperative care data mostly focused on postsurgical admission facilities and rarely on postoperative analgesia. This aspect of anesthetic management in PAS patients is a promising area for future research.

The aim of the outcome assessment was to identify valuable measures for assessing the influence of anesthetic management on postsurgical recovery in PAS patients. Our analysis revealed that these data were rarely and inconsistently reported. Studies tended to include only basic patient-centered outcomes, such as the length of hospital stay, the need for postoperative mechanical ventilation in mothers, and Apgar scores in neonatal assessments. Other outcomes (if reported) were often presented in a non-anesthesiology context, such as the urinary catheter removal time. The main procedure-related data (i.e., blood loss, procedure duration, and transfusion data) are important for comparing various PAS management methods; however, their value in the assessment of postsurgical recovery (which is crucial from an anesthesiologic perspective) is limited. To properly assess how this issue is influenced by various approaches to anesthetic management, a common reporting of various outcomes such as postoperative opioid consumption, time to first mobilization, and successful breastfeeding or neonatal respiratory outcomes would be advisable. Therefore, future research and management protocols should consider the adoption of a standardized patient-centered outcome set, such as the set used by Sultan et al., which was also used in this review [17]. This measure would benefit both the research and practice of anesthetic management for PAS patients.

### 4.1. Strengths and Limitations

This review has several limitations. The assessment of the broad spectrum of strategies for managing anesthesia in PAS patients may have restricted the in-depth assessment of specific issues. Due to the fact that we focused on the extraction of management data (rather than the interpretation of the outcomes), we also included studies with objectives that were not originally focused on anesthesia. This approach enabled us to obtain multiple valuable data points but may have limited the value of particular assessments to some extent, such as the outcome assessment (as the limited reporting of patient-centered outcomes may be explained by the factual aim of the study). In addition, our screening was limited to articles published in English, which may have resulted in missing relevant publications.

### 4.2. Conclusions

The management of anesthesia in PAS patients is complex and requires proper strategies. This scoping review aligns well with the current approach to anesthetic management in PAS patients treated in centers with experienced MDTs. Various approaches to different aspects of anesthesia were identified; however, due to the paucity of interventional studies, the most effective methods for managing anesthesia in these patients have not been fully established and require in-depth assessments and discussion among MDTs.

This review identified and confirmed previously mentioned key knowledge gaps that should be addressed in future studies, such as the optimal mode of anesthesia and its adoption in various surgical and interventional techniques, the utility of advanced monitoring, the effect of goal-directed hemodynamic management and fluid resuscitation strategies, the structure approach to blood product management and hemostasis, or optimal postoperative care.

### 4.3. Implications

Further research is needed to adapt anesthesia methods to patient-specific risks and requirements associated with adopted surgical strategies. To generate higher-quality evidence, researchers should concentrate on increasing the quality of published studies. First, focus should be placed on interventional studies or large observational studies rather than case reports. Anesthetic management protocols and data should be provided in detail by authors whenever possible, and the reason for the selection of specific approaches should be described. A consensus on the reported outcomes is advised. Consistent reporting of patient-centered outcomes would promote enhanced recovery and facilitate the assessment of adopted strategies of anesthetic management, eventually improving evidence gaps.

## Figures and Tables

**Table 1:** Research questions on anesthetic management topics and references for corresponding evidence differentiated by study design. RCT—randomized control trial.

Topic and Question	RCT	Quasiexperimental Studies	Cohort Studies	Case Series	Case Reports
**Structure of anesthetic service**					
➢ What surgical procedures for PAS patients require anesthetic services?	[18,19,20]	[21,22,23,24,25,26,27,28,29]	[30,31,32,33,34,35,36,37,38,39,40,41,42,43,44,45,46,47,48,49,50,51]	[52,53,54,55,56,57,58,59,60,61,62,63,64,65,66,67,68,69,70,71,72,73,74,75,76,77,78,79,80,81,82]	[83,84,85,86,87,88,89,90,91,92,93,94,95,96,97,98,99,100,101,102,103,104,105,106,107,108,109,110,111,112,113,114,115,116,117,118,119,120,121,122,123,124,125,126,127,128,129,130,131,132,133,134,135,136,137,138,139,140,141,142,143,144,145,146,147]
➢ What is the surgical setting where PAS surgeries are performed?	[18,19,20]	[21,22,23,24,25,26,28,29]	[30,31,32,33,34,35,43,45,48,51]	[52,53,55,57,58,59,60,61,66,68,69,71,72,73,74,77,80,82]	[83,84,85,86,87,89,91,93,94,95,100,101,102,103,104,105,106,107,108,109,110,111,112,114,115,116,118,120,121,122,123,124,126,127,129,130,131,132,135,136,143,144,146,147]
➢ Is MDT involvement reported in studies about the management of anesthesia in PAS patients?	[19]	[21,22,26,27,28,29]	[30,31,32,34,35,37,38,40,47,50,89,92]	[52,53,54,57,60,61,62,66,68,74,77,80,81]	[85,86,88,89,91,93,94,95,99,102,103,104,105,107,108,112,113,114,115,116,118,125,126,128,129,130,134,136,139,146]
➢ What are the typical components of a perioperative protocol reported in studies of anesthesia in PAS patients?		[21]	[31,32,33]		
➢ When the MDT is reported, what key personnel are specifically listed as members of the team?		[21,22,26,27,28]	[30,31,32,34,37,38,40,47,50,92]	[52,57,60,61,62,66,74,77,80]	[85,88,89,91,93,94,99,102,104,105,107,108,112,113,114,115,116,118,126,129,134,135,136,146]
➢ What is the optimal size and composition of the clinical team delivering anesthesia services for PAS surgery?		[21]	[30,31,34,35,36,49]	[52,53,54]	[86,87,88,89,90,91]
➢ Does the perioperative protocol include a risk stratification system with implications for multidisciplinary planning?		[22]	[37]		
➢ How do risk stratification systems inform the composition of the team and location of the surgery?					
**Anesthetic technique and drugs**					
➢ What modes of anesthesia are reportedly used for cesarean section in patients diagnosed with PAS?	[18,19,20]	[21,23,24,25,26,27,28,29]	[30,31,32,33,34,35,36,37,38,39,40,41,42,43,44,45,46,47,48,49,50,51,92]	[52,53,54,55,56,57,58,59,60,61,62,63,64,65,66,67,68,69,70,71,72,73,74,75,76,77,78,79,80,81,82]	[83,84,85,86,87,88,89,90,91,93,94,95,96,97,98,99,100,101,102,103,104,105,106,107,108,109,110,111,112,113,114,115,116,117,118,119,120,121,122,123,124,125,126,127,128,129,130,131,132,133,134,135,136,137,138,139,140,141,142,143,144,145,146,147]
➢ What is the preferred technique for regional anesthesia?	[18]	[21,22,23,24,25,26,27,28,29]	[30,31,32,33,34,35,37,38,39,40,41,42,43,44,45,46,47,48,49,51,70,92]	[52,54,55,56,58,59,60,61,62,63,66,67,68,69,71,72,73,74,75,76,79,80,81,82]	[83,84,85,86,89,90,91,95,96,97,99,100,101,103,105,109,110,111,113,114,115,116,117,118,119,120,121,122,123,124,127,130,134,136,138,141,143,144,147]
➢ Do studies about the management of anesthesia in PAS patients report the reason for the choice of a particular technique?	[18,19,20]	[21,23,25,26,28]	[30,32,37,40,42,44,45,47,48,49,50,70]	[52,54,56,57,58,59,60,63,66,71,73,77,82]	[85,87,90,91,93,94,95,96,97,102,108,110,111,113,114,119,120,121,122,124,125,126,129,131,132,135,136,137,138,139,140,141,142,143,145,146,147]
➢ For studies that initiate the procedure with neuraxial anesthesia, what are the indications for conversion to general anesthesia?	[18]	[21,27,28]	[33,34,38,39,42,44,46,49,92]	[54,55,56,58,60,62,69,71,73,75,76,80,81,82,121]	[83,86,90,97,100,101,102,103,109,111,113,116,117,118,120,130,134,136,141,143,144]
➢ How does the preferred technique differ between cases completed under planned general anesthesia, planned neuraxial anesthesia, and planned conversion from neuraxial to general anesthesia?	[18,19,20]	[21,23,24,25,26,27,28,29]	[30,31,32,33,34,35,36,37,38,39,40,42,43,44,45,46,47,48,49,51,70,92]	[52,54,55,56,57,59,60,61,62,63,64,65,66,67,68,69,71,72,74,75,77,78,79,80,81,82]	[84,85,87,88,89,91,93,94,95,96,98,99,102,104,106,107,108,109,110,111,112,113,114,115,116,119,121,122,123,124,125,126,127,128,129,131,132,134,135,137,138,139,140,142,143,145,146,147]
➢ What is the preferred timing of neuraxial block placement relative to the IR procedure?			[33,48,70]	[55,58,60,68,74,82]	[53,84,85,89,95,109,113,114,115,119,120,124,132,134,135,136,137,147]
➢ Do studies about the anesthetic management of PAS patients report the preferred technique of anesthetic maintenance?	[18,20]	[24,27]	[33,36,37,39,44,92]	[54,56,57,58,63,64,65,69,72,74,75,77,80,82]	[83,86,87,88,93,94,96,97,99,102,103,104,105,106,107,120,129,131,132,133,135,139,140,141,142,144]
➢ What are the recommendations for the anesthetic induction technique for the intraoperative conversion of neuraxial anesthesia to general anesthesia?	[18]	[21,27,28]	[33,38,39,44,46,92]	[54,55,56,58,59,60,62,69,71,73,75,76,80,81,82]	[83,86,90,97,100,101,102,103,109,111,117,120,130,134,136,141,143,144]
**Periprocedural monitoring**					
➢ What is the standard of monitoring anesthesia during PAS surgery?	[20]	[24,27]	[48]	[52,53,54,55,56,57,58,59,61,62,63,65,67,69,71,72,73,74,76,77,79,80,81,82]	[83,85,88,93,94,97,103,105,107,109,113,114,115,118,119,120,121,122,127,129,130,132,134,135,137,141,142,145,146]
➢ What types of invasive monitoring are used?	[18,19,20]	[21,23,24,25,28]	[30,31,32,33,37,39,40,44,45,47,48,49,50,92]	[52,53,54,55,56,57,58,59,61,62,63,65,67,69,71,72,73,74,76,77,79,80,81,82]	[83,84,85,86,87,89,90,91,93,94,95,96,97,98,99,100,101,102,104,105,106,107,108,111,112,113,114,116,118,120,121,123,124,125,126,128,129,131,132,133,134,135,136,137,138,139,140,141,142,143,144,146,147]
➢ What types of hemodynamic monitoring are used?	[20]	[25,27]		[52]	[84,91,106,113,118,120,132]
**Hemodynamic management and fluid therapy**					
➢ Uses of what types of fluid are typically reported?	[18,20]	[25,26,27]	[32,33,35,37,41,43,45,49,50]	[52,54,56,57,58,61,67,69,71,72,75,78,79]	[83,85,86,87,88,89,93,94,95,96,97,98,100,101,103,105,106,107,108,109,110,114,120,122,128,129,132,136,138,139,140,141,142,143,144,145,146]
➢ What data on vascular access are reported?	[18,19,20]	[21,23,24,26,27,28]	[30,32,33,37,40,44,45,46,47,48,49,50,70,92]	[52,53,54,55,56,57,58,59,61,62,63,65,66,67,69,71,72,74,75,76,77,78,80,81,82]	[83,85,86,87,88,89,90,91,93,95,97,98,99,101,102,103,104,105,106,107,108,111,112,114,115,120,121,122,123,124,126,127,128,129,130,132,133,134,135,136,137,138,139,140,141,142,143,144,146,147]
➢ Did any studies report the use of a risk stratification system to define appropriate venous access?		[21]	[40]	[62]	
➢ What vasoactive drugs are reported?	[18]	[27]	[33,39,44]	[52,56,61,62,64,66,67,69,71,72,75,80]	[83,84,86,87,88,89,91,93,95,100,101,102,103,105,106,109,110,118,120,121,122,129,131,137,138,142,144]
**Blood product management and hemostasis**					
➢ Is the use of massive transfusion protocols reported during PAS surgery?		[21,22]	[30,32,40]	[56,61,63,69]	[83,87,88,91,94,101,103,104,105,107,108,121,125,128,140,145]
➢ Is the method of blood loss assessment reported?	[19]	[21,23,24,26]	[34,35,37]	[52,58,69,71,77,148]	[72,121,122,129]
➢ Do the authors report fixed-ratio transfusion, goal-directed blood product transfusion, or a combination of the two protocols?		[23]	[30,32]	[52,54,55,61,62,63,69]	[102,104,107,122,143]
➢ What are the types of hemostatic agents used?	[19]	[22,26]	[30,33,34,37,40,41,43,48,49,51]	[52,54,56,59,61,67,69,72,73,75,76,80,81]	[86,87,88,89,90,91,97,99,101,102,103,107,108,109,114,115,117,119,120,123,125,127,128,135,137,141,143]
➢ Is the use of blood salvage techniques during PAS surgery reported and is the risk of amniotic fluid embolism an important restriction associated with its use?	[18]	[22,25,26]	[39,47,48]	[55,59,64,65,66,67,73,75,76,77,80,81,82]	[85,88,89,93,106,107,108,109,110,111,112,114,115,131,133,139,143]
**Postoperative care**					
➢ How often is ICU admission after PAS procedures reported?		[23,25,26,27]	[31,32,33,34,36,37,39,40,42,43,45,46,47,49,51,72,92]	[52,53,54,56,59,60,61,62,64,66,67,68,69,71,73,75,76,77,78,79,80,81]	[84,86,87,88,90,91,96,97,99,101,105,106,107,118,119,120,129,130,132,133,135,136,140,141,142,143,145]
➢ What are the indications for ICU admission?		[23]	[32,33,39,41,45,46,49,72,92]	[52,53,54,60,61,62,64,66,68,69,71,77,78]	[84,87,88,90,91,96,99,101,105,106,107,118,119,120,123,129,133,140,141,142,143,145]
➢ What data regarding postoperative pain therapy are reported?	[18,19]		[31,33,36,37,38,44]	[52,56,57,58,60,67,69,71,75,79,80]	[89,95,102,107,111,112,115,119,122,135,138,141,142,146]
➢ What is the role of peripheral nerve blocks in PAS surgery?		[25]			[95,103,114,115,116]
**Mother and newborn outcomes**					
➢ Do studies about the anesthetic management of PAS patients report the core outcomes for enhanced recovery after cesarean delivery?	[18,19]	[21,23,25,26,28,29]	[32,33,34,35,36,37,38,39,40,41,42,44,45,46,47,48,50,51,70,92]	[52,53,54,55,56,57,58,60,61,62,64,65,66,67,69,71,74,75,76,78,80,81]	[83,84,85,86,87,88,89,90,93,94,95,96,98,99,100,101,102,103,104,105,106,107,108,109,110,112,113,114,115,116,118,119,120,121,122,123,126,127,128,129,130,131,132,133,134,135,136,138,139,140,141,142,143,144,145,146,147]

**Table 2:** Study characteristics.

Study Characteristics:	No. of Studies (%)
**Country:**	
Africa (Egypt)	2 (1.5%)
Australia (Australia, New Zealand)	3 (2.3%)
Asia (China, India, Indonesia, Israel, Japan, Korea, Pakistan, Qatar, Saudi Arabia, Taiwan, Turkey, UAE)	50 (38.5%)
Europe (Germany, Greece, Italy, Ireland, Romania, Russia, Spain, Sweden, UK)	31 (23.9%)
North America (Canada, Mexico, USA)	39 (30%)
South America (Colombia)	5 (3.8%)
**Study type:**	
Case report	56 (43%)
Case series	24 (18.5%)
Cohort study	22 (17%)
Conference abstract	11 (8.4%)
Quasiexperimental study	7 (5.4%)
Randomized controlled trial	3 (2.3%)
Other	6 (4.6%)
Case study	1 (0.8%)
**Publication year**	
2020–2024	52 (40%)
2015–2019	42 (32.3%)
2010–2014	22 (16.9%)
2005–2009	8 (6.2%)
2000–2004	2 (1.5%)
1995–1999	3 (2.3%)
1990–1994	
1985–1989	1 (0.8%)
1980–1984	

**Table 3:** (A) Identified reasons for the choice of GA or RA. (B) Additional elective reasons for conversion from RA to GA and their relation to the stage of the procedure (if identifiable). * If the data in the stage-of-the-procedure columns do not add to “Total” in the remaining studies, such a relation could not be identified. CS, cesarean section; CSE, combined spinal and epidural anesthesia; ERAS, enhanced recovery after surgery; GA, general anesthesia; RA, regional anesthesia; PAS, placenta accreta spectrum; PPH, peripartum hemorrhage.

A. Reason for Choosing GA or RA (No. of Included Studies)				B. Reason for Conversion from RA to GA (No. of Included Studies)					
General Anesthesia		Regional Anesthesia			Total *	Preoperative	After IR—Before Surgery	After Cesarean Delivery—Before Hysterectomy	During Hysterectomy
risk of PPH and/or coagulopathy	20	patient request	5	severe bleeding	14	-	-	9	-
surgical factors (duration, extent, possible extension)	14	reduced pharmacological exposure of the fetus	5	hemodynamic instability	10	-	-	-	1
risk of hemodynamic instability	12	risk associated with airway instrumentation	4	inadequate analgesia/block	9	5	-	1	-
management protocol	4	management protocol	3	patient discomfort	7	1	-	1	2
airway protection or ventilation control	4	ERAS protocol	2	duration of the procedure	5	-	-	1	-
patient request	4	low preoperative risk of PAS/low surgical risk	2	surgical request	2	-	-	-	2
risk of balloon repositioning	3	advantage of RA in CS	1	patient preference	2	-	-	-	-
urgency	3	degree of placental invasion	1	extent of surgical exposure	3	-	-	1	-
degree of placental invasion	3	intraoperative diagnosis—decision to continue RA	1	anticipated hemodynamic instability	2	-	-	1	1
heparin use	3	obesity-associated risks of GA	1	bleeding risk	2	-	-	-	-
influence on placental separation	2	possible extension of the duration of anesthesia (CSE)	1	consciousness reduction	1	-	-	1	-
need for muscle relaxation	2	parturient stable hemodynamic condition	1	hem epidural catheter	1	-	-	-	-
contraindications for RA	2	surgical factors (duration, extent, possible extension)	1	balloon displacement and cardiac arrest	1	-	-	-	1
intraoperative pain control	1	patient condition	1	inability to cope	1	-	-	-	-
difficult airway	1	better postoperative analgesia	1	prolonged immobility	1	-	-	-	-
advantage of GA during hysterectomy	1	reduced blood loss during RA	1	respiratory distress	1	-	-	-	1
high risk of conversion to GA	1	reduced risk of postoperative thrombosis	1						
anesthesiologist preference	1	anesthesiologist preference	1						
patient condition	1								
ability to use TEE	1								

**Table 4:** Reported outcomes from the essential outcomes list (PONV, postoperative nausea and vomiting; pICU, pediatric intensive care unit).

General Outcomes	No. of Studies
Length of hospital stay	80
Maternal hospital readmission rate	7
Maternal reattendance rate	0
**Maternal Outcomes**	
Mechanical ventilation/sedation time	26
Postoperative opioid use	23
Postoperative opioid consumption	2
Maternal satisfaction with analgesia	2
Timing of extubation	52
Postoperative vasopressor use	6
PONV	0
Obstetric Quality of Recovery-10 score	0
Breastfeeding time	0
Duration of preoperative fasting (liquids)	0
Postoperative time to first fluid intake	1
Postoperative time to first solid food intake	1
Time to first mobilization	2
Time to urinary catheter removal	7
**Neonatal Outcomes**	
Apgar scale	60
pICU admission	15
Breastfeeding by time of discharge	2

## Data Availability

The data presented in this study are available on request from the corresponding author.

## Supplementary Materials

The following supporting information can be downloaded at: https://www.mdpi.com/article/10.3390/jcm14134738/s1, Supplementary Section S1: Search Strategy and criteria; Supplementary Section S2: List of excluded studies; Supplementary Section S3 Characteristics of the included studies.; PRISMA-ScR Fillable Checklist.

## Abbreviations

The following abbreviations are used in this manuscript:

AFE	Amniotic fluid embolism
CD	Cesarean delivery
CSE	Combined spinal and epidural anesthesia
ERAS	Enhanced recovery after surgery
GA	General anesthesia
ICU	Intensive care unit
IR	Interventional radiology
MDT	Multidisciplinary team
MTP	Massive transfusion protocol
PAS	Placenta accreta spectrum
pICU	Pediatric intensive care unit
PONV	Postoperative nausea and vomiting
PPH	Peripartum hemorrhage
RCT	Randomized control trial
TEG	Thromboelastography
TIVA	Total intravenous anesthesia
VAS	Visual analog scale
