# Supplementary material for: Anesthetic Management for Patients with Placenta Accreta Spectrum: A Scoping Review
Source: J Clin Med. 2025 Jul 4;14(13):4738. doi: 10.3390/jcm14134738 (PMC12251126; doi:10.3390/jcm14134738)
Supplement: Supplementary file 1 [file jcm-14-04738-s001.zip › Section S1 - Search Strategy and criteria.pdf]

Table S1 Full inclusion and exclusion criteria

Inclusion criteria
• **Topic**<br>1. Study on cesarean delivery with suspected or confirmed PAS presenting data on seven aspects of anesthetic management:<br>I. the structure and setting of anesthetic services<br>II. anesthetic technique and pharmacology<br>III. monitoring<br>IV. hemodynamic management and fluid therapy<br>V. blood product management and hemostasis<br>VI. postoperative care<br>VII. maternal and newborn outcomes.<br>• **Study type**<br>1. experimental and quasi-experimental study:<br>▪ randomized controlled trial (RCTs) and nonrandomized controlled trial<br>▪ before and after study<br>2. analytical and descriptive observational study:<br>▪ prospective and retrospective cohort study<br>▪ case-control study<br>▪ case series<br>▪ case reports<br>3. conference abstracts, correspondence papers or letters containing adequate data on anesthetic management<br>• **Study language: English**<br>• **Publication date: 1980 - present**
**Exclusion criteria**
• **Topic**<br>Study on anesthetic managment for miscarriages and abortions associated with abnormal placentation<br>• **Study type**<br>1. Text and opinion paper

Table S2 PUBMED – medline. Search strategy

Search	Query	Records retrieved
#1	"Placenta Accreta"[Mesh]	2564
#2	placenta accret* OR placenta* incret* OR placenta* percret*	3874
#3	"abnormally invasive placenta*" OR "abnormal placental attachment" OR "morbidly adherent placenta*" OR "abnormal placent*" OR "placenta implantation" OR "adherent placenta" OR "invasive placenta*" OR “placental adhesion disorder*”	2325
#4	#1 OR #2 OR #3	5294
#5	“Anesthesia”[Mesh] OR "Monitoring, Physiologic"[Mesh] OR “perioperative care” [Mesh] OR “fluid therapy” [Mesh] OR "Blood Transfusion"[Mesh]	528098
#5	anesthe* OR anaesthe*	765877
#6	"fluid therapy" OR "hemodynamic monitoring" OR "intraoperative care" OR fluid OR “regional block*” OR “fascial block” OR “peripheral block” OR “spinal” OR “epidural” OR “neuraxial” OR “transfus*” OR “coagul*”	1537270
#7	#5 OR #6 OR #7	2424080
#8	#4 AND #7	1289
	Limited to English; 1.01.1980 – 17.11.2024	1182

Table S3 EMBASE - search strategy

Search	Query	Records retrieved
S1	EMB.EXACT.EXPLODE("placenta accreta")	5218
S2	“placenta* accret*” OR “placenta* incret*” OR “placenta* percret*”	6167
S3	"abnormally invasive placenta*" OR "abnormal placental attachment" OR "morbidly adherent placenta*" OR "abnormal placent*" OR "placenta implantation" OR "adherent placenta" OR "invasive placenta*" OR “placental adhesion disorder*”	3913
S4	S1 OR S2 OR S3	
S5	EMB.EXACT.EXPLODE("anesthesiological procedure") OR EMB.EXACT.EXPLODE("patient monitoring") OR EMB.EXACT.EXPLODE("perioperative care") OR MJEMB.EXACT("rehydration") OR MJEMB.EXACT("fluid resuscitation") OR MJEMB.EXACT("intravenous drug administration") OR EMB.EXACT.EXPLODE("blood transfusion")	1456286
S6	an?esthe*	328578
S7	“fluid therapy” OR "hemodynamic monitoring" OR "intraoperative care" OR “perioperative care” OR fluid OR “regional block*” OR “fascial block” OR “peripheral block” OR “spinal” OR “epidural” OR “neuraxial” OR “transfus*” OR “coagul*”	1956280
S8	S5 OR S6 OR S7	3054670
S9	S4 AND S8	2695
	Limited to English; 1.01.1980 – 17.11.2024	2556

Table S4 CINAHL – search strategy

Search	Query	records retrieved
S1	(MH "Placenta Accreta")	1294
S2	“placenta* accret*” OR “placenta* incret*” OR “placenta* percret*”	1964
S3	"abnormally invasive placenta*" OR "abnormal placental attachment" OR "morbidly adherent placenta*" OR "abnormal placent*" OR "placenta implantation" OR "adherent placenta" OR "invasive placenta*" OR “placental adhesion disorder*”	1005
S4	S1 OR S2 OR S3	2585
S5	(MH "Anesthesia and Analgesia+") OR (MH "Monitoring, Physiologic+") OR (MH "Perioperative Care+") OR (MH "Fluid Therapy+") OR (MH "Blood Transfusion+")	268880
S6	an#esthe*	103387
S7	“fluid therapy” OR "hemodynamic monitoring" OR "intraoperative care" OR “perioperative care” OR fluid OR “regional block*” OR “fascial block” OR “peripheral block” OR “spinal” OR “epidural” OR “neuraxial” OR “transfus*” OR “coagul*”	265015
S8	S5 OR S6 OR S7	511828
S9	S4 AND S8	515
	Limited to English; 1.01.1980 – 17.11.2024	512

Table S5 SCOPUS – search strategy

Search	Query	Records.retrieved
	(“placenta* accret*” OR “placent* incret*” OR “placenta* percret*” OR "abnormally invasive placenta*" OR "abnormal placental attachment" OR "morbidly adherent placenta*" OR "abnormal placent*" OR "placenta implantation" OR "adherent placenta" OR "invasive placenta*" OR “placental adhesion disorder*”) AND (An?esthe* OR monitoring OR “perioperative care” OR “intraoperative care” OR “perioperative management” OR “intraoperative management” OR "fluid therapy" OR “regional block*” OR “fascial block” OR “peripheral block” OR “spinal” OR “epidural” OR “neuraxial” OR “transfus*” OR “coagul*”)	1916
	Limited to English; 1980 – 2024	1841

Table S6 Web of Science – search strategy

Search	Query	Records retrieved
	(“placenta* accret*” OR “placent* incret*” OR “placenta* percret*” OR "abnormally invasive placenta*" OR "abnormal placental attachment" OR "morbidly adherent placenta*" OR "abnormal placent*" OR "placenta implantation" OR "adherent placenta" OR "invasive placenta*" OR “placental adhesion disorder*”) AND (An?esthe* OR monitoring OR “perioperative care” OR “intraoperative care” OR “perioperative management” OR “intraoperative management” OR "fluid therapy" OR “regional block*” OR “fascial block” OR “peripheral block” OR “spinal” OR “epidural” OR “neuraxial” OR “transfus*” OR “coagul*”)	1071
	Limited to English; 1980 – 2024	1033

Table S7 Google Scholar – search strategy

Search	Query	Records retrieved
	(placenta accreta) AND anesthesia	2

Table S8 Google Advanced Search – search strategy

Search	Query	Records retrieved
	"placenta accreta" and anesthesia	2
