# Supplementary material for: Anesthetic Management for Patients with Placenta Accreta Spectrum: A Scoping Review
Source: J Clin Med. 2025 Jul 4;14(13):4738. doi: 10.3390/jcm14134738 (PMC12251126; doi:10.3390/jcm14134738)
Supplement: Supplementary file 1 [file jcm-14-04738-s001.zip › Section S2 - list of excluded studies.pdf]

Table S1. List of excluded studies

	Title	Reason for exclusion
1.	Alhashim, Z. G., Alzayer, Z. A., Alensaif, A. A., Al Darwish, H. A., Almomen, M. A., & Alnsaif, J. M. (2023). Blood Transfusion Predictors in Cesarean Sections for Pregnancies With Placenta Accreta and Placenta Previa: A Monocentric Tertiary Experience. *Cureus*, *15*(10), e47648. https://doi.org/10.7759/cureus.47648	**Ineligible context**
2.	Arif, F. & William, J. F. Prophylactic iliac artery balloons in the management of placenta percreta. *Regional Anesthesia and Pain Medicine* **36**, E206 (2011).	**Ineligible data**
3.	Arif, N., Zafar, B., Ahmed, R. Q. & Shehzad, F. Antenatal Diagnosis, Surgical Approach and Maternal Morbidity with Placenta Accreta. *Pakistan Armed Forces Medical Journal* **72**, 1678–1681 (2022).	**Ineligible context**
4.	Bakacak, Z. *et al.* An examination by year of cases applied with caesarean hysterectomy because of placenta percreta in a tertiary centre: a retrospective cohort study. *Ginekologia polska* **92**, 284–288 (2021).	**Ineligible data**
5.	Bampoe, S., Vinayagam, D., Odor, P., Thilaganathan, B. & Khalil, A. Intra-operative haemodynamic monitoring during high-risk caesarean section: A method comparison study between bioreactance (NICOM®) and pressure waveform analysis (LidCOrapid®). *Anaesthesia* **72**, 66 (2017).	**Ineligible context**<br>**Comment:** No data on anesthetic management of PAS patients
6.	Banovska, J. *et al.* Efficiency of temporary balloon occlusion of iliac arteries in patients at high hemorrhagic risk undergoing cesarean section. *European Journal of Anaesthesiology* **30**, 176 (2013).	**Ineligible data**
7.	Bard, M., Bersot, Y., Legros, V., Raimond, E. & Malinovsky, J. M. Hemodynamic monitoring by the aortic velocity-time integral in supra sternal Doppler echocardiography and total cavo-pulmonary derivation in cesarean delivery. *Journal of Clinical Anesthesia* **46**, 99–100 (2018).	**Ineligible context**
8.	Barry, M. A., Byrne, B. & Fanning, R. A. Peripartum hysterectomy-an anaesthesia perspective. *International Journal of Obstetric Anesthesia* **21**, S34 (2012).	**Ineligible data**<br>Comment: Limited anesthetic management data
9.	Bartels, H. C., Lalor, J. G., Walsh, D., Nieto-Calvache, A. J., Terlizzi, K., Cooney, N., Palacios-Jaraquemada, J. M., O'Flaherty, D., MacColgain, S., Ffrench-O'Carroll, R., & Brennan, D. J. (2024). Anesthesia and postpartum pain management for placenta accreta spectrum: The patient perspective and recommendations for care. *International journal of gynaecology and obstetrics: the official organ of the International Federation of Gynaecology and Obstetrics*, **164**(3), 992–1000. https://doi.org/10.1002/ijgo.15125	**Ineligible study type**<br>Comment: Survey analysis
10.	Bartels, H. C., Walsh, D., Nieto-Calvache, A. J., Lalor, J., Terlezzi, K., Cooney, N., Palacios-Jaraquemada, J. M., O'Flaherty, D., MacColgain, S., Ffrench-O'Carroll, R., & Brennan, D. J. (2024). Anesthesia and postpartum pain management for placenta accreta spectrum: The healthcare provider perspective. *International journal of gynaecology and obstetrics: the official organ of the International Federation of Gynaecology and Obstetrics*, **164**(3), 964–970. https://doi.org/10.1002/ijgo.15096	**Ineligible study type**<br>Comment: Survey analysis
11.	Bartlett, A. *et al.* Abnormally invasive placenta: An evaluation of care over a three-year period. *International Journal of Obstetric Anesthesia* **31**, S48 (2017).	**Ineligible data**
12.	Beattie, E. M. L., McDonnell, N., Knight, T. & Nathan, L. Case series of 136 women delivering with placenta acereta, increta or percreta: A 14-year retrospective study. *International Journal of Obstetric Anesthesia* **35**, S20 (2018).	**Ineligible data**
13.	Beavan, P. S. & Eagland, K. G. Prophylactic internal iliac artery balloon catheters for caesarean section in a Jehovah's Witness with sickle cell disease and placenta praevia. *International Journal of Obstetric Anesthesia* **21**, S35 (2012).	**Ineligible condition**

14.	Bhatia, N., Arora, S., Bhukal, I. & Padmanaban, A. Placenta percreta in a parturient with uncorrected Tetralogy of Fallot. *International Journal of Obstetric Anesthesia* **22**, 358–360 (2013).	**Ineligible condition**<br>**Comment:** Unusual case - low quantity of extractable data
15.	Bilge, A., Feray, A., Ahmet, G. & Dilek, U. Anesthetic Approach in Placental Invasion Anomalies. *Gynecology and Women's Health Research*.	**Ineligible data**
16.	Blumenthal, E. *et al.* Intra-aortic balloon placement and management of placenta percreta. *Obstetrics and Gynecology* **127**, 100S (2016).	**Ineligible data**
17.	Braun, T. *et al.* Abnormally invasive placenta (AIP): Pre-cesarean amnion drainage to facilitate exteriorization of the gravid uterus through a transverse skin incision. *Journal of Perinatal Medicine* **47**, 12–15 (2019).	**Ineligible data**
18.	Brookfield, K. F., Goodnough, L. T., Lyell, D. J. & Butwick, A. J. Perioperative and transfusion outcomes in women undergoing cesarean hysterectomy for abnormal placentation. *Transfusion* **54**, 1530–1536 (2014).	**Ineligible context**
19.	Butwick, A. J. Managing patients with abnormal placentation: What are the best anesthetic and transfusion strategies? *Anesthesiology* **116**, 1156–1157 (2012).	**Ineligible study type**<br>**Comment:** Letter to the editor without extractable data
20.	Campbell, N. Anaesthetic management of caesarean section for placenta praevia - A five-year retrospective study. *Anaesthesia and Intensive Care* **40**, 877 (2012).	**Ineligible data**
21.	Chan, S. & James, R. Case report: Important considerations for neuraxial anaesthesia in the multidisciplinary management of the patient with placenta accreta. *Regional Anesthesia and Pain Medicine* **70**, A18 (2021).	**Ineligible data**
22.	Chaturvedi, S., Panicker, J. & Mohan, S. B. Massive blood transfusion in a post cesarean patient with placenta praevia. *Egyptian Journal of Anaesthesia* **28**, 293–297 (2012).	**Ineligible context**
23.	Chestnut, D. H., Dewan, D. M., Redick, L. F., Caton, D. & Spielman, F. J. Anesthetic management for obstetric hysterectomy: A multi-institutional study. *Anesthesiology* **70**, 607–610 (1989).	**Ineligible data**
24.	Clausen, C. *et al.* Balloon occlusion of the internal iliac arteries in the multidisciplinary management of placenta percreta. *Acta Obstetricia et Gynecologica Scandinavica* **92**, 386–391 (2013).	**Ineligible data**
25.	Clifford, C., Aimee, R., Uppal, S. & Carver, A. 233 The use of rotational thromboelastometry (ROTEM) in cesarean hysterectomies for placenta accreta spectrum (PAS). *American Journal of Obstetrics and Gynecology* **224**, S154 (2021).	**Ineligible context**
26.	Corso, K. J. & Robertson, S. Multidisciplinary Approach to Reduce the Risk of Morbidity and Mortality Related to Placenta Accreta...Proceedings of the 2015 AWHONN Convention. *JOGNN: Journal of Obstetric, Gynecologic & Neonatal Nursing* **44**, S83-4 (2015).	**Ineligible data**
27.	Crofton-Martin, J. C. & Woolnough, M. J. P.23 Effect of ROTEM on management of abnormally invasive placentas in a tertiary referral centre. *International Journal of Obstetric Anesthesia* **46**, (2021).	**Ineligible context**
28.	Davies, M. H., Brunning, T., Kerr, J. & Cullis, K. Anaesthesia for abnormally invasive placenta: a single-institution case series. *International Journal of Obstetric Anesthesia* **32**, 95–96 (2017).	**Ineligible data**
29.	Dergunova-Zimina, A. *et al.* Massive bleeding in parturient: Challenge for anesthesiologist. *Anesthesia and Analgesia* **133**, 1047 (2021).	**Ineligible data**<br>Comment: Limited anesthetic management data
30.	Desai, G. *et al.* Placenta percreta-Challenges in management. *Indian Journal of Urology* **35**, S38 (2019).	**Ineligible data**
31.	Doğru, Ş., Akkuş, F., Atci, A. A., Metin, Ü. S., Uyar, M., & Acar, A. (2024). Fetal and maternal outcomes of segmental uterine resection in emergency and planned placenta percreta deliveries. *Obstetrics & gynecology science*, *67*(1), 58–66. https://doi.org/10.5468/ogs.23154	**Ineligible context**
32.	Dunn, R., MacGillivray, R. & Strachan, L. Undiagnosed placenta percreta with massive antepartum haemorrhage in a non-obstetric centre.	**Ineligible data**

	Anaesthesia **73**, 80 (2018).	
33.	Faris, A. S., Jeyaraj, L., Goheen, S. & Dumitrascu, G. Placenta percreta; Challenges and keys for success (case report). *Regional Anesthesia and Pain Medicine* **36**, E239 (2011).	**Ineligible data**
34.	Flores-Mendoza, H., Shehata, N., Murji, A., Allen, L. M., Kingdom, J. C., Windrim, R. C., Carvalho, J. C. A., Ravi Chandran, A., Papalia, N., & Hobson, S. R. (2023). Use of intraoperative red cell salvage in the contemporary management of placenta accreta spectrum disorders. Canadian journal of anaesthesia = Journal canadien d'anesthesie, 70(9), 1544–1546. https://doi.org/10.1007/s12630-023-02490-7	**Ineligible data**
35.	Fowler, A., Brockelsby, J., Patil, A. & Foukaneli, T. Planning for massive haemorrhage. *Transfusion Medicine* **26**, 57 (2016).	**Ineligible data**
36.	Fox, K. A. *et al.* 285: General endotracheal anesthesia used at the time of delivery for morbidly adherent placenta is associated with increased need for neonatal resuscitation and short-term respiratory morbidity. *American Journal of Obstetrics & Gynecology* **218**, S181–S181 (2018).	**Ineligible data**<br>**Comment:** Conference abstract – lack of anesthetic management data
37.	Fratto, V. M. *et al.* Assessing the multidisciplinary team approaches to placenta accreta spectrum across five institutions within the University of California fetal Consortium (UCfC). *Journal of Maternal-Fetal and Neonatal Medicine* **34**, 2971–2976 (2021).	**Ineligible data**
38.	Fratto, V. *et al.* UC Fetal Consortium (UCfC) multidisciplinary team approach to invasive placenta: Management across a five institution consortium. *American Journal of Obstetrics and Gynecology* **218**, S127–S128 (2018).	**Ineligible data**
39.	Frederiksen, M. C., Glassenberg, R. & Stika, C. S. Placenta previa: A 22-year analysis. *American Journal of Obstetrics and Gynecology* **180**, 1432–1437 (1999).	**Ineligible condition**
40.	Fretwell, D., Smith, M., Martin, E., Manecke, G. R. & Cronin, B. Epidural Intravascular Injection Detection by Transthoracic Echocardiography. *Journal of Cardiothoracic and Vascular Anesthesia* **34**, 1288–1291 (2020).	**Ineligible condition**<br>Comment: Adverse event report with low quantity of extractable data
41.	Fulzele, M., Shyamal, A., Shaikh, J. V., Bokariya, P. & Fulzele, S. Retrospective Analysis of Anesthetic Management in Cesarean Section of Pregnant Women with Placental Anomaly: An Institutional Based Study. *European Journal of Molecular and Clinical Medicine* **9**, 5945–5949 (2022).	**Ineligible data**
42.	Gagnon, J., Boucher, L., Kaufman, I., Brown, R. & Moore, A. Iliac artery rupture related to balloon insertion for placenta accreta causing maternal hemorrhage and neonatal compromise. *Canadian Journal of Anesthesia* **60**, 1212–1217 (2013).	**Ineligible condition**<br>**Comment:** Report of an adverse event with low quantity of extractable data
43.	Gatta, L. A., Grotegut, C. A., West Honart, A., Craig, A. M., Salinaro, J. R., Weber, J. M., Alvarez Secord, A., Habib, A. S., Pabon-Ramos, W., Ronald, J., & Gilner, J. B. (2024). Multivessel embolization followed by immediate hysterectomy for placenta accreta spectrum. American journal of obstetrics & gynecology MFM, 6(10), 101466. https://doi.org/10.1016/j.ajogmf.2024.101466	**Ineligible data**
44.	Gidiri, M., Noble, W., Rafique, Z., Patil, K. & Lindow, S. W. Caesarean section for placenta praevia complicated by postpartum haemorrhage managed successfully with recombinant activated human coagulation Factor VIIa. *Journal of Obstetrics and Gynaecology* **24**, 925–926 (2004).	**Ineligible context**
45.	Ben Goodman, Adam Shonfeld. 6299225 Caesarean-hysterectomy for placenta accreta in a Jehovah's Witness declining allogeneic blood transfusion, International Journal of Obstetric Anesthesia, Volume 58, Supplement 1,2024	**Ineligible data**
46.	Grant, T. R., Ellinas, E. H., Kula, A. O. & Muravyeva, M. Y. Risk-stratification, resource availability, and choice of surgical location for the management of parturients with abnormal placentation: a survey of United States-based obstetric anesthesiologists. *International Journal of Obstetric Anesthesia* **34**, 56–66 (2018).	**Ineligible data**

47.	Guasch, E. *et al.* Obstetrical hemorrhage and blood transfusion during caesarean section: Which risk factors? *European Journal of Anaesthesiology* **28**, 83 (2011).	**Ineligible condition**
48.	Gunaydin, B. Refresher course: Managing a patient with abnormal placentation. *Regional Anesthesia and Pain Medicine* **40**, e30–e31 (2015).	**Ineligible study type**<br>**Comment:** Review with low quantity of extractable anaesthetic management data
49.	Hall, T. *et al.* Prenatal sonographic diagnosis of placenta accreta--impact on maternal and neonatal outcomes. *Journal of clinical ultrasound : JCU* **42**, 449–455 (2014).	**Ineligible context**
50.	Harrison, E. A., Yentis, S. M. & Bennett, A. M. Anaphylaxis during caesarean section in a patient with undiagnosed placenta accreta: It never rains but it pours! *International Journal of Obstetric Anesthesia* **8**, 279–283 (1999).	**Ineligible context**
51. .	Hasegawa, J. *et al.* Maternal deaths in Japan due to abnormally invasive placenta. *International Journal of Gynecology and Obstetrics* **140**, 375–376 (2018).	**Ineligible phenomena of interest**
52.	Hatfield, T., Kraus, H., McConnell, D. & Nageotte, M. Synchronous autotransfusion during cesarean hysterectomy. *American Journal of Obstetrics and Gynecology* **202**, e15–e16 (2010).	**Ineligible data**
53.	Hilario, J. Application of a new cesarean technique on teen patients with placental accretion. *International Journal of Gynecology and Obstetrics* **143**, 642 (2018).	**Ineligible data**
54.	Hoffman, M., Molina, O. V., Pedroza, C., Chauhan, S. & Sibai, B. General vs neuraxial anesthesia for cesarean hysterectomy: A secondary analysis. *American Journal of Obstetrics and Gynecology* **216**, S455–S456 (2017).	**Ineligible data**<br>**Comment :** No detailed data on anaesthetic management
55.	Hort, K., Okutani, R., Shimizu, A. & Nakata, K. Prophylactic balloon occlusion of common iliac arteries in a parturient with placenta previa: A new option of hybrid operating room for high-risk cesarean section. *Anesthesia and Resuscitation* **51**, 49–51 (2015).	**Full text not retrieved**
56.	Huang, F. *et al.* Association of the placenta accreta spectrum score and estimated blood loss in placenta accreta spectrum patients with placenta previa: a retrospective cohort study. *J Anesth* **36**, 715–722 (2022).	**Ineligible data**
57.	Hung, A., Ramos, S. Z., Wiley, R., Sawyer, K., Gupta, M., Chauhan, S. P., Deshmukh, U., Shainker, S., Samshirsaz, A., & Wagner, S. (2024). Evidence-based surgery for cesarean hysterectomy secondary to placenta accreta spectrum: A systematic review. *European journal of obstetrics, gynecology, and reproductive biology*, *302*, 155–166.	**Ineligible study type**<br>**Comment: Systematic** Review with low quantity of extractable anaesthetic management data
58.	Huwe, V. Y. & Mullen, M. Can We Save Her Without Giving Blood? An Incredible Case of Obstetric Hemorrhage. *JOGNN: Journal of Obstetric, Gynecologic & Neonatal Nursing* **43**, S99–S100 (2014).	**Ineligible context**
59.	Ibrahim, T. Efficacy of tranexamic acid in reducing blood loss, blood and blood products requirements in Cesarian sections for patients with placenta accreta. *AIN SHAMS JOURNAL OF ANESTHESIOLOGY* **11**, (2019).	**Ineligible data**
60.	Ioscovich, A. *et al.* Israeli national survey of anesthesia practice related to placenta previa and accreta. *Regional Anesthesia and Pain Medicine* **40**, e177 (2015).	**Ineligible study type**<br>**Comment:** Survey analysis - no extractable data
61.	Ioscovich, A. *et al.* Israeli survey of anesthesia practice related to placenta previa and accreta. *Acta Anaesthesiologica Scandinavica* **60**, 457–464 (2016).	**Ineligible study type**<br>**Comment:** Survey analysis - no extractable data
62.	Jee, Y., Lee, H. J., Kim, Y. J., Kim, D. Y. & Woo, J. H. Association between anesthetic method and postpartum hemorrhage in Korea based on National Health Insurance Service data. *Anesth Pain Med (Seoul)* **17**, 165–172 (2022).	**Ineligible data**
63.	Kalopita, K. *et al.* A four-year review of peripartum hysterectomies in a tertiary teaching hospital in Greece. *Anesthesia and Analgesia* **133**, 1074	**Ineligible condition**<br>Comment: PAS patients data inseparable

	(2021).	from other patients with other diagnosis - limited anaesthetic management data
64.	Kandemir, H., Kirtis, E., Bulbul, G. A., Dogan, S., Mendilcioglu, I., Sanhal, C. Y., Sakinci, M., & Dogan, N. U. (2024). Intraoperative and Postoperative Outcomes of Pfannenstiel and Midline Skin Incisions in Placenta Accreta Spectrum Disorders: Single-Center Experience. *Medicina (Kaunas, Lithuania)*, *60*(7), 1102.	**Ineligible data**
65.	Katyayani, K. Management of placenta accreta at a district general hospital. *Anaesthesia* **72**, 84 (2017).	**Ineligible data**
66.	Kremer, M. E., Ladella, S. & Schmidt, L. J. Timing of administration of tranexamic acid during cesarean hysterectomy for placenta accreta. *Reproductive Sciences* **26**, 252A (2019).	**Ineligible context**
67.	Kuczkowski, K. M. & Miller, T. Cesarean hysterectomy for placenta percreta invading the anterior abdominal wall: anesthetic considerations--a case report. *Middle East journal of anesthesiology* **19**, 1105–1109 (2008).	**Ineligible data**
68.	Kumar, R., Sahay, N., Naaz, S. & Kumar, R. Anesthetic management of complicated placenta percreta. *AIN SHAMS JOURNAL OF ANESTHESIOLOGY* **14**, (2022).	**Ineligible condition**<br>**Comment:** PAS with premature foetal demise
69.	Kuromaki, K. *et al.* Autologous blood transfusion for the patient with placenta previa complicated by placenta increta: A case report. *Asia-Oceania Journal of Obstetrics and Gynaecology* **20**, 155–159 (1994).	**Ineligible data**
70.	Lan, J.-Y., Wang, M.-H., Fan, S.-Z. & Chen, L.-K. Impact of anesthetic methods on neonatal outcome in women receiving temporary balloon occlusion of the common iliac artery during cesarean section for placenta accreta. *Taiwanese Journal of Obstetrics and Gynecology* **50**, 515–517 (2011).	**Duplicate study**
71.	Lau, T. K. & Leung, T. Y. Prenatal diagnosis of morbidly adherent placenta. *International Journal of Obstetric Anesthesia* **20**, 107–109 (2011).	**Ineligible study type**
72.	Lekic, Z., Ahmed, E., Peeker, R., Sporrong, T. & Karlsson, O. A standardized multidisciplinary approach and a modified surgical technique for the management of abnormally invasive placenta; Striking differences in blood loss and need for transfusion: A retrospective comparative study. *Acta Anaesthesiologica Scandinavica* **61**, 981–982 (2017).	**Study data published**<br>Comment: Data duplicated with the another included study of this author
73.	Levy, R. A., Diala, P. C., Rothschild, H. T., Correa, J., Lehrman, E., Markley, J. C., Poder, L., Rabban, J., Chen, L. M., Gras, J., Sobhani, N. C., Cassidy, A. G., & Chapman, J. S. (2024). Roadmap to safety: a single center study of evidence-informed approach to placenta accreta spectrum. Frontiers in surgery, 11, 1347549.	**Ineligible data**
74.	Lim, W. H. ow, Pavlov, T. & Dennis, A. E. Analysis of emergency peripartum hysterectomy in Northern Tasmania. *The Australian journal of rural health* **22**, 235–240 (2014).	**Ineligible data**
75.	Liu, X., Zhu, Y., Ke, D., Liu, D. & Zhu, Z. Mode of anesthesia for cesarean delivery with pernicious placenta previa - a retrospective study. *Ginekologia polska* **91**, 91–94 (2020).	Ineligible data<br>Comment: limited anaesthetic management data
76.	Livingstone, K. & McGrady, E. Major obstetric haemorrhage management in placental pathology cases. *International Journal of Obstetric Anesthesia* **26**, S18 (2016).	**Ineligible data**
77.	Lubis, M. P., Yaznil, M. R., Barus, M. N. G., Asroel, E. M. & Faustine, M. Maternal outcomes of hysterectomy and conservative surgery in placenta accreta. *Current Women's Health Reviews* **16**, 201–205 (2020).	**Ineligible data**
78.	Macarthur, A. Anesthetic considerations for placenta accrete in reply. *INTERNATIONAL JOURNAL OF OBSTETRIC ANESTHESIA* **21**, 381–381 (2012).	**Ineligible data**
79.	Marshall, N. B. & Catling, S. Cardiac arrest due to uterine inversion during caesarean section. *International Journal of Obstetric Anesthesia* **19**, 231–234 (2010).	**Ineligible context**

80.	McCall, S. J., Mansour, S., Khazaal, J., Kayem, G., DeJong, J., & Chahine, R. (2024). Obstetric and haematological management and outcomes of women with placenta accreta spectrum by planned or urgent delivery: Secondary data analysis of a public referral hospital in Lebanon. PloS one, 19(5), e0302366.	**Ineligible data**
81.	McCarthy, C. M., & Donnelly, J. C. (2024). Care considerations for caesarean births in a non-obstetric hospital. *Obstetric medicine*, 1753495X241256219. Advance online publication. https://doi.org/10.1177/1753495X241256219	**Ineligible data**
82.	McDowell, L., McDonnell, C. & Holland, J. A rare case of expectant management for placenta percreta in a district general hospital. *ANAESTHESIA* **75**, 13–13 (2020).	**Ineligible data**
83.	Miller, S. E. *et al.* Red Blood Cell Transfusion in Patients with Placenta Accreta Spectrum: A Systematic Review and Meta-analysis. *Obstetrics and Gynecology* **141**, 49–58 (2023).	**Ineligible data**
84.	Misugi, T. *et al.* Non-invasive continuous blood pressure monitoring using the ClearSight system for pregnant women at high risks of post-partum hemorrhage: comparison with invasive blood pressure monitoring during cesarean section. *OBSTETRICS & GYNECOLOGY SCIENCE* **65**, 325–334 (2022).	Ineligible condition<br>Comment: Only 30 % of patients with PAS diagnosis- data inseparable
85.	Moore, A., Laxton, C. & Weale, N. Use of vaginal cell salvage in an unusual case of abnormally invasive placenta. *International Journal of Obstetric Anesthesia* **50**, 46 (2022).	Ineligible condition<br>Comment: Pregnancy termination
86.	Morato R, Dinis M, Lerias M, et al#35898 Placenta percreta: A near missRegional Anesthesia & Pain Medicine 2023;48:A240.	**Ineligible data**
87.	Munoz, J. L., Blankenship, L. M., Ireland, K. E., McCann, G. A. & Ramsey, P. S. Outcomes of the UT-PAS protocol: uterine artery embolization and tranexamic acid for placenta accreta spectrum. *American Journal of Obstetrics and Gynecology* **228**, S66 (2023).	Ineligible data<br>Comment: Lack of anaesthetic management data
88.	Munoz, J. L., Hernandez, B., Curbelo, J., Ramsey, P. S. & Ireland, K. E. Effect of anesthesia selection on neonatal outcomes in cesarean hysterectomies for placenta accreta spectrum (PAS). *Journal of Perinatal Medicine* **50**, 1210–1214 (2022).	Ineligible data<br>Comment: Lack of anaesthetic management details
89.	Munoz, J. L. *et al.* Impact of placenta accreta spectrum (PAS) pathology on neonatal respiratory outcomes in cesarean hysterectomies - do wycięcia. *Journal of Maternal-Fetal and Neonatal Medicine* **35**, 10692–10697 (2022).	**Ineligible data**
90.	Munoz, J. L., Pfeiffer, A. F., Curbelo, J., Ramsey, P. S. & Ireland, K. E. Neuraxial to general anesthesia conversion has equitable intraoperative and improved post-operative outcomes compared to general anesthesia in cesarean hysterectomy for placenta accreta spectrum (PAS). *Journal of Maternal-Fetal and Neonatal Medicine* **35**, 8640–8644 (2022).	**Ineligible data**
91.	Mushtaq, S., Kurdi, W. & Al-Shammari, M. Prophylactic catheters placement and intraoperative internal iliac artery embolisation in a patient with placenta accreta. *Journal of Obstetrics and Gynaecology* **27**, 853–855 (2007).	**Ineligible data**
92.	Nair, A. S. Balloon occlusion of internal iliac arteries in placenta accreta! *Saudi Journal of Anaesthesia* **11**, 245–246 (2017).	**Ineligible study type**<br>**Comment:** Letter to the editor without extractable data
93.	Nakago, S., Kato, H., Shibata, T., Nishijima, K. & Kotsuji, F. Minimizing abdominal incision for transverse uterine fundal incision by aspiration of amniotic fluid and reduction of uterine size. *Journal of Obstetrics and Gynaecology Research* **47**, 900–903 (2021).	**Ineligible data**
94.	Neely, D. & Elnour, S. Anaesthesia for abnormally invasive placenta: cell salvage and tranexamic acid. *International Journal of Obstetric Anesthesia* **32**, 94–95 (2017).	**Ineligible study type**
95.	Nguyen-Lu, N. *et al.* Anesthetic management of women with invasive placentation: A review of 55 cases. *Canadian Journal of Anesthesia* **60**,	**Ineligible data**

	S99–S100 (2013).	
96.	Nguyen, P. N., Vuong, A. D. B., & Pham, X. T. T. (2024). Neonatal outcomes in the surgical management of placenta accreta spectrum disorders: a retrospective single-center observational study from 468 Vietnamese pregnancies beyond 28 weeks of gestation. *BMC pregnancy and childbirth*, *24*(1), 228.	**Ineligible data**
97.	Nieto-Calvache, Á. J., Aryananda, R. A., Palacios-Jaraquemada, J. M., Cininta, N., Grace, A., Benavides-Calvache, J. P., Campos, C. I., Messa-Bryon, A., Vallecilla, L., Sarria, D., Galindo, J. S., Galindo-Velasco, V., Rivera-Torres, L. F., Burgos-Luna, J. M., & Bhide, A. (2024). One-step conservative surgery vs hysterectomy for placenta accreta spectrum: a feasibility randomized controlled trial. American journal of obstetrics & gynecology MFM, 6(6), 101333.	**Ineligible data**
98.	Nieto-Calvache, A. J. *et al.* 414 Multidisciplinary management facilitates the application of neuraxial anesthesia in placenta accreta spectrum. *American Journal of Obstetrics and Gynecology* **224**, S266 (2021).	**Duplicate study**
99.	Nieto-Calvache, A. J. *et al.* Maternal hemodynamics during aortic occlusion with REBOA in patients with placenta accreta spectrum disorder. *Journal of Maternal-Fetal & Neonatal Medicine* **35**, 5217–5223 (2022).	**Ineligible context**
100.	Nieto-Calvache, A. J. *et al.* Maternal hemodynamics during aortic occlusion with REBOA in patients with placenta accreta spectrum disorder. *The Journal of Maternal-Fetal & Neonatal Medicine* **35**, 5217–5223 (2022).	**Duplicate study**
101.	Nieto-Calvache, A. J. *et al.* A systematic multidisciplinary initiative may reduce the need for blood products in patients with abnormally invasive placenta. *Journal of Maternal-Fetal and Neonatal Medicine* **35**, 738–744 (2022).	**Duplicate study**
102.	Nieto-Calvache, A. J. *et al.* A multidisciplinary approach and implementation of a specialized hemorrhage control team improves outcomes for placenta accreta spectrum. *Journal of Trauma and Acute Care Surgery* **90**, 807–816 (2021).	**Ineligible data**
103.	Nivatpumin, P., Nithi-Uthai, J., Lertbunnaphong, T., Sukcharoen, N., Soponsiripakdee, T., & Yonphan, P. (2024). Perioperative outcomes and causes of postpartum hemorrhage in patients undergoing cesarean delivery in Thailand: A comprehensive retrospective study. *PloS one*, *19*(4), e0300620.	**Ineligible data**
104.	O'Flaherty, D., Enright, S., Ainle, F. N. & Hayes, N. Intraoperative cell salvage as part of a blood conservation strategy in an obstetric population with abnormal placentation at a large Irish tertiary referral centre: an observational study. *Irish Journal of Medical Science* **189**, 1053–1060 (2020).	**Ineligible context**
105.	Okafor, U. V., Ezegwui, H. U. & Okezie, O. Anaesthetic challenges in emergency peripartum hysterectomy in West Africa: A Nigerian perspective. *Southern African Journal of Anaesthesia and Analgesia* **16**, 8–11 (2010).	**Ineligible condition**<br>**Comment:** Only 25% of patients with PAS diagnosis - data inseparable
106.	Parekh, N., Husaini, S. W. U. & Russell, I. F. Caesarean section for placenta praevia: A retrospective study of anaesthetic management. *British Journal of Anaesthesia* **84**, 725–730 (2000).	**Ineligible condition**
107.	Pri-Paz, S. *et al.* Cesarean hysterectomy requiring emergent thoracotomy: A case report of a complication of placenta percreta requiring a multidisciplinary effort. *Journal of Reproductive Medicine for the Obstetrician and Gynecologist* **57**, 58–60 (2012).	**Ineligible context**
108.	Purwosunu, Y. & Haloho, A. H. Placenta accreta complicated with peripartum cardiomyopathy. *BMJ Case Reports* **2018**, 1–3 (2018).	**Ineligible context**
109.	Pyregov, A. & Korolev, A. Regional anesthesia and placenta accreta. *Regional Anesthesia and Pain Medicine* **43**, e149 (2018).	**Ineligible data**
110.	Quist-Nelson, J. *et al.* The compliance with a patient-safety bundle for management of placenta accreta spectrum†. *Journal of Maternal-Fetal and Neonatal Medicine* **34**, 2880–2886 (2021).	**Ineligible data**

111.	Reitman, E., Devine, P. C., Laifer-Narin, S. L. & Flood, P. Case scenario: Perioperative management of a multigravida at 34-week gestation diagnosed with abnormal placentation. *Anesthesiology* **115**, 852–857 (2011).	**Ineligible study type**
112.	Riazanova, O. V. *et al.* Open versus endovascular REBOA control of blood loss during cesarean delivery in the placenta accreta spectrum: A single-center retrospective case control study. *European Journal of Obstetrics and Gynecology and Reproductive Biology* **258**, 23–28 (2021).	**Ineligible context**
113.	Saha, P. K. *et al.* An alternate surgical approach to reduce hemorrhage and complications during cesarean hysterectomy for adherent placenta. *European Journal of Obstetrics and Gynecology and Reproductive Biology* **228**, 215–220 (2018).	**Ineligible context**
114.	Salmanian, B. *et al.* Maternal morbidity in patients with placenta accreta treated with and without a multidisciplinary approach. *American Journal of Obstetrics and Gynecology* **210**, S300 (2014).	**Ineligible data**
115.	Seoud, M., Chahine, R., Arab, W., Jaafar, I., Moubarak, M., El Kassis, N., Abdallah, R., Ramadan, M. K., Nassar, M., Nassar, A., Ayoub, E. N., & Atallah, D. (2024). The Lebanese percreta group: A retrospective cohort study of both radical and conservative management outcomes of abnormally invasive placenta. *International journal of gynaecology and obstetrics: the official organ of the International Federation of Gynaecology and Obstetrics*, *167*(2), 675–684.	**Ineligible data**
116.	Seto, S., Itakura, A., Okagaki, R., Suzuki, M. & Ishihara, O. An algorithm for the management of coagulopathy from postpartum hemorrhage, using fibrinogen concentrate as first-line therapy. *International Journal of Obstetric Anesthesia* **32**, 11–16 (2017).	**Ineligible context**
117.	Shah, M. K. Emergency Caesarean Hysterectomy, a ten year retrospective review in KK Women's and Children's Hospital, Singapore. *Acta Anaesthesiologica Scandinavica* **59**, 24 (2015).	**Ineligible data**
118.	Shamshirsaz, A. A. *et al.* Outcomes of planned compared with urgent deliveries using a multidisciplinary team approach for morbidly adherent placenta. *Obstetrics and Gynecology* **131**, 234–241 (2018).	**Ineligible context**
119.	Sharma, B. *et al.* Peripartum hysterectomy in a tertiary care hospital: Epidemiology and outcomesImproving outcomes for peripartum hysterectomy: Still a long way to go! *Journal of Anaesthesiology Clinical Pharmacology* **33**, 324–328 (2017).	**Ineligible context**
120.	Shaylor, R. *et al.* Pre-delivery remifentanil infusion for placenta accreta cesarean delivery under general anesthesia: an observational study. *Journal of Maternal-Fetal & Neonatal Medicine* **29**, 2793–2797 (2016).	**Ineligible data**
121.	Shifman, E., Kulikov, A., Zhilin, A., Matkovski, A. & Mamayev, S. Recombinant activated VII factor at massive bleeding in case of invasive placenta. *Intensive Care Medicine Experimental* **5**, (2017).	**Ineligible context**
122.	Simonetti, F. M., Algeri, P., Ferrante, I., Pirola, S., Carnelli, M., Patanè, L., Fierro, G., & Frigerio, L. (2023). Placenta Accreta Spectrum Disorders: How to reduce maternal transfusion? A center experience on extraperitoneal retrograde hysterectomy. *European journal of obstetrics, gynecology, and reproductive biology*, *287*, 148–154.	**Ineligible context**
123.	Singh, S., Carusi, D. A., Wang, P., Reitman-Ivashkov, E., Landau, R., Fields, K. G., Weiniger, C. F., & Farber, M. K. (2023). External Validation of a Multivariable Prediction Model for Placenta Accreta Spectrum. *Anesthesia and analgesia*, *137*(3), 537–547.	**Ineligible data**
124.	Sivasankar, C. Perioperative management of undiagnosed placenta percreta: Case report and management strategies. *International Journal of Women's Health* **4**, 451–454 (2012).	**Ineligible data**
125.	Smith, K. M. A., McGuinness, N., McFarland, R. & McNamara, H. P.27 Anaesthesia at the extremes of obstetric haemorrhage. *International Journal of Obstetric Anesthesia* **46**, (2021).	**Ineligible condition**<br>**Comment**: Only 40% of patients with PAS diagnosis - data inseparable
126.	Smulian, J. C. *et al.* Invasive placental disease: The impact of a multi-disciplinary approach to management. *American Journal of Obstetrics*	**Ineligible data**

	and Gynecology **214**, S156 (2016).	
127.	Snegovskikh, D. *et al.* Point-of-care viscoelastic testing improves the outcome of pregnancies complicated by severe postpartum hemorrhage. *Journal of Clinical Anesthesia* **44**, 50–56 (2018).	**Ineligible context**
128.	Soleymani majd, H. *et al.* The modified radical peripartum cesarean hysterectomy (Soleymani-Alazzam-Collins technique): a systematic, safe procedure for the management of severe placenta accreta spectrum. *American Journal of Obstetrics and Gynecology* **225**, 175.e1-175.e10 (2021).	**Ineligible data**
129.	Solórzano Vázquez, J. F., Ruvalcaba Ortiz, G., Hernández Higareda, S. & Morales de Avila, C. N. Blood loss and use of haemoderivates in cases of caesarean hysterectomy due to placentary acretism. *Perinatologia y Reproduccion Humana* **31**, 55–61 (2017).	**Ineligible language**
130.	Sritharan, B. & Gyampoh, B. Management of atypical abdominal pain involving the multidisciplinary team. *BJOG: An International Journal of Obstetrics and Gynaecology* **122**, 223 (2015).	**Ineligible data**
131.	Stanleigh, J. *et al.* Maternal and neonatal outcomes following a proactive peripartum multidisciplinary management protocol for placenta creta spectrum as compared to the urgent delivery. *European Journal of Obstetrics & Gynecology & Reproductive Biology* **237**, 139–144 (2019).	**Ineligible data**
132.	Stubbs, M. K., Wellbeloved, M. A. & Vally, J. C. The management of patients with placenta percreta: A case series comparing the use of resuscitative endovascular balloon occlusion of the aorta with aortic cross clamp. *Indian J Anaesth* **64**, 520–523 (2020).	**Ineligible data**
133.	Subramaniam, R., Dadhwal, V. & Gamanagatti, S. Abnormally adherent placenta: Current concepts and anesthetic management. *Trends in Anaesthesia and Critical Care* **24**, 32–39 (2019).	**Ineligible study type**<br>Comment**:** Review article
134.	Sun, M. S. *et al.* Anesthetic management in parturients with uterine rupture preoperatively--report of two cases. *Acta anaesthesiologica Sinica* **35**, 167–170 (1997).	**Full text not retrieved**
135.	Supraptomo, R. T. & Sunjoyo, A. Regional Anesthesia Conversion to General Anesthesia during Cesarean Section of a Woman with Antepartum Hemorrhage due to Placenta Previa and Ovarian Cyst with Moderate Confirmed COVID-19: A. *Open Access Macedonian Journal of Medical Sciences* **10**, 1946–1949 (2022).	**Ineligible condition**<br>**Comment:** Lack of PAS diagnosis
136.	Sylvester-Armstrong, K., Reeder, C., Patrick, K. & Genc, M. R. Improved management of placenta accreta spectrum disorders: Experience from a single institution. *Journal of Perinatal Medicine* **50**, 286–293 (2022).	**Ineligible data**
137.	Tarantino, F. & Calì, G. Placenta Accreta: Management Protocol and Use of Epidural Anesthesia. *Clinical Management Issues* **15**, 31–34 (2021).	**Ineligible study type**<br>**Comment:** Protocol presentation - no extractable data
138.	Taylor, N. J. & Russell, R. Anaesthesia for abnormally invasive placenta. *International Journal of Obstetric Anesthesia* **26**, S16 (2016).	**Ineligible data**<br>**Comment**: Data duplicated from other study by the same authors
139.	Taylor, N. & Russell, R. Anaesthesia for abnormally invasive placenta: cell salvage and tranexamic acid reply. *INTERNATIONAL JOURNAL OF OBSTETRIC ANESTHESIA* **32**, 95–95 (2017).	**Study data published**
140.	Teare, J., Evans, E., Belli, A. & Wendler, R. Sciatic nerve ischaemia after iliac artery occlusion balloon catheter placement for placenta percreta. *International Journal of Obstetric Anesthesia* **23**, 178–181 (2014).	**Ineligible context**<br>**Comment:** Report of an adverse event with low quantity of extractable data
141.	Teixeira, B., Pinto, P. V., Realista, R., Silva, M., Costa, A., Machado, A. P., & Moucho, M. (2023). Placenta Accreta Spectrum Disorders - The Impact of the Creation of a Multidisciplinary Team on Maternal Outcomes in Portugal. Patologia do espectro do acretismo placentário – O	**Ineligible data**

	impacto da criação de uma equipa multidisciplinar nos desfechos maternos em Portugal. *Revista brasileira de ginecologia e obstetricia : revista da Federacao Brasileira das Sociedades de Ginecologia e Obstetricia*, *45*(12), e747–e753.	
142.	Thadkapally, S., Chakravarthy, K., Alagandala, A. & Nagamani, S. A retrospective study of the efficacy of external abdominal aortic compression in reducing postpartum haemorrhage. *Anesthesia and Analgesia* **133**, 1130 (2021).	**Ineligible condition**
143.	Tussey, C. & Olson, C. Creating a Multidisciplinary Placenta Accreta Program. *Nursing for Women's Health* **22**, 372–386 (2018).	**Ineligible context**
144.	Ueda, K., Miyoshi, K. & Kai, S. The perioperative management of cesarean section in a patient with FXIII deficiency and placenta previa: a case report. *JA Clinical Reports* **8**, (2022).	**Ineligible context**
145..	Vinayagam, D., Bampoe, S., Thilaganathan, B. & Khalil, A. G3. Intraoperative haemodynamic monitoring during high-risk caesarean section: a comparison between bioreactance (NICOM R) and pressure waveform analysis (LidCOrapidR): preliminary results. *Journal of Maternal-Fetal and Neonatal Medicine* **29**, 34 (2016).	**Ineligible context**<br>**Comment:** No data on anaesthetic PAS management
146..	Vuilleumier, P. H. & Surbek, D. Anesthesiologic management of major obstetrical hemorrhage. *Trends in Anaesthesia and Critical Care* **5**, 167–178 (2015).	**Ineligible study type**<br>**Comment**: Review article
147.	Wagner, W. *et al.* Implementation and Outcomes of a Model of Care for Placenta Accreta Spectrum in a Community-Based Private Practice. *American Journal of Perinatology* (2022) doi:10.1055/s-0042-1749664.	
148.	Wali, A. Placenta previa/accreta: Repeat cesarean section regional vs. general. *Journal of Anaesthesiology Clinical Pharmacology* **15**, 510–523 (1999).	**Ineligible study type**
149.	Walker, M. G. *et al.* Obstetric and Anaesthesia Checklists for the Management of Morbidly Adherent Placenta. *Journal of Obstetrics and Gynaecology Canada* **38**, 1015–1023 (2016).	**Ineligible study type**<br>**Comment:** Checklist and procedure presentation - no data from practice
150.	Waters, J. H., Lukauskiene, E. & Anderson, M. E. Intraoperative Blood Salvage during Cesarean Delivery in a Patient with β Thalassemia Intermedia. *Anesthesia and Analgesia* **97**, 1808–1809 (2003).	**Ineligible context**
151.	Weiniger, C. F. & Levin, P. D. Anaphylaxis during cesarean section; the importance of a good history. *International Journal of Obstetric Anesthesia* **18**, S14 (2009).	**Ineligible condition**
152.	Weiniger, C. F., Weissman, C., Ginosar, Y., Elram, T. & Eid, L. Management of 69 consecutive cases of suspected placenta accreta. *International Journal of Obstetric Anesthesia* **18**, S48 (2009).	**Ineligible condition**
153.	Weinstein, J., Muhalwes, R., Ronenson, A., Halpern, S. H., Grisaru-Granovsky, S., Akawi, T., Gozal, Y., Shatalin, D., & Ioscovich, A. (2024). The anesthetic approach to repeated cesarean sections: A prospective cohort study. *European journal of obstetrics & gynecology and reproductive biology: X*, *22*, 100301.	**Ineligible context**
154.	Wen, O., Azer, M. & Boss, L. Establishing and Maintaining stop before You Block-A continuing quality improvement campaign. *Anaesthesia and Intensive Care* **48**, 32–33 (2020).	**Ineligible context**<br>**Comment:** Report of an adverse event, low quantity of eligible data
155.	P115 An evaluation of anaesthesia for abnormally invasive placenta utilising interventional radiology Whitehouse, J. et al. International Journal of Obstetric Anesthesia, Volume 54, 103777	**Ineligible data**
156.	Wijesinghe, V., Rishard, M., & Srisanjeevan, S. (2022). Quality of surgical management of placenta accreta spectrum in a tertiary center in Sri Lanka: baseline study for quality improvement project: problems and solutions. BMC pregnancy and childbirth, 22(1), 509.	**Ineligible context**
157.	Williams, M. G., Lyons, G., McLure, H. & Wilson, R. Prophylactic uterine artery balloon catheters for suspected placenta accreta. *International Journal of Obstetric Anesthesia* **18**, S49 (2009).	**Ineligible data**

158.	Williamson, R. M. Anaesthetic considerations for placenta accreta. *International Journal of Obstetric Anesthesia* **21**, 380–381 (2012).	**Ineligible study type**
159.	Wong, Yiu & Lo, Tsz & Chan, Viola & Ng, Vivian & Yung, Wai & Tsang, Hin & Koo, Chi & Cho, Danny & Lau, Wl & Leung, Wing. (2023). Conservative management for placenta accreta spectrum disorders: experience of a regional hospital from 2013 to 2021. Hong Kong Journal of Gynaecology, Obstetrics and Midwifery. 23. 93-100.	**Ineligible data**
160.	Young, H., Ehrig, J. C., Hammonds, K. & Hofkamp, M. P. Effect of a placenta accreta spectrum multidisciplinary team and checklist on maternal outcomes for planned hysterectomy at time of cesarean delivery. *Baylor University Medical Center Proceedings* **35**, 755–758 (2022).	**Ineligible data**
161.	Yu, Q., Chen, B., Li, P. & Luo, L. Cell salvage for Rh-negative patients without anti-D immunoglobulin. *International Journal of Obstetric Anesthesia* **49**, (2022).	**Ineligible context**
162.	Zbede, A., Berkenstadt, H. & Zahavi, G. Massive transfusion protocol in placenta accreta patients undergoing cesarean section. *Anesthesia and Analgesia* **133**, 1141 (2021).	**Ineligible data**
163.	Zdanowicz, J. *et al.* Impact of patient blood management in obstetrics on postpartum hemorrhage treatment: A retrospective analysis. *Anesthesia and Analgesia* **133**, 58 (2021).	**Ineligible condition**<br>**Comment:** Data od PAS patients inseparable from other PPH cases
