# Supplementary material for: Anesthetic Management for Patients with Placenta Accreta Spectrum: A Scoping Review
Source: J Clin Med. 2025 Jul 4;14(13):4738. doi: 10.3390/jcm14134738 (PMC12251126; doi:10.3390/jcm14134738)
Supplement: Supplementary file 1 [file jcm-14-04738-s001.zip › Section S3 Characteristics of the included studies.pdf]

Table S1. Characteristics of the included studies.

Author (year)	Study type	Diagnosis	Described surgical approach and interventional radiology use	Detailed surgery data	Topics covered	Reported outcomes	Critical appraisal													
							**Critical Appraisal Checklist used**	Q1	Q2	Q3	Q4	Q5	Q6	Q7	Q8	Q9	Q10	Q11	Q12	Q13
Example	*RCT, Quasiexperimental Study, Cohort Study, Case Series, Case Report, etc.*	*Total number of PAS cases and types of PAS*	*Conservative or extirpative approach*<br>*Type of radiological intervention:*<br>*IIABO – internal iliac artery balloon occlusion*<br>*UABO – uterine artery balloon occlusion*<br>*IAABO – intra abdominal aortic balloon occlusion*<br>*FABO – femoral artery balloon occlusion*<br>*UAE – uterine artery embolization*<br>*IIAE – internal iliac artery embolization*<br>*Additional procedures:*<br>*REBOA - resuscitative endovascular balloon occlusion of the aorta*<br>*cystoscopy*	Elective or emergency procedure<br>Type of surgery<br>1. Cesarean delivery with uterine preservation<br>2. Cesarean hysterectomy<br>3. Cesarean delivery with anticipated hysterectomy<br>4. staged delivery	I - Structure and setting of anesthetic service<br>II - Anesthetic technique and pharmacology<br>III - Monitoring<br>IV - Hemodynamic management and fluid therapy<br>V - Blood product management and hemostasis<br>VI - Postoperative care<br>VII - Mother and newborn outcomes		*Name of the checklist used for critical appraisal (number of questions in the checklist)*	Y – *YES,* N – *NO*, U- unclear, N/A – not applicable												
Abdullah S[1] (2022)	RCT	n - 108<br>not specified	conservative and extirpative	elective not specified	II, III, IV		**JBI Critical Appraisal Checklist for Randomized Controlled Trials (Q13)**	**Y**	**U**	**Y**	**N**	**N**	**N**	**U**	**Y**	**Y**	**Y**	**U**	**Y**	**Y**
Ahmad M[2] (2021)	Case Series	n – 30<br>not specified	conservative and extirpative	elective and emergency not specified	II, III, IV, V		**JBI Critical Appraisal Checklist for Case Series (Q10)**	**Y**	**U**	**N/A**	**Y**	**Y**	**N**	**Y**	**N**	**N**	**N/A**			
Akinaga C[3] (2024)	Case Series – letter to the editor	n - 12<br>not specified	surgical approach and interventional radiology method not specified	not specified	II, III, VII	**Apgar scale**<br>**pICU admission**	**Critical Appraisal Checklist of conference abstracts, letters to the editor and correspondence papers (Q6)**	**Y**	**Y**	**Y**	**Y**	**Y**	**Y**							
Alford B[4] (2019)	Case Report	n – 1<br>suspected PAS	conservative<br>IIABO	elective cesarean delivery with uterine preservation	I, II, III, V, VI, VII	**Length of hospital stay**	**JBI Critical Appraisal Checklist for Case Reports (Q8)**	**Y**	**Y**	**Y**	**Y**	**Y**	**N**	**N/A**	**Y**					
Alvarado-Ramos S[5] (2020)	Case Series	n - 43<br>placenta accreta - 40<br>placenta percreta - 3	extirpative	elective cesarean hysterectomy	I, II, III, IV, V, VI, VII	**Length of hospital stay**	**JBI Critical Appraisal Checklist for Case Series (Q10)**	**Y**	**Y**	**N/A**	**Y**	**Y**	**Y**	**Y**	**Y**	**Y**	**Y**			

Angstmann T[6] (2010)	Cohort Study	n - 25<br>placenta accreta - 7<br>placenta increta - 5<br>placenta percreta - 14	extirpative	elective staged delivery with delayed hysterectomy<br>elective cesarean hysterectomy<br>elective cesarean delivery with uterine preservation<br>emergency not specified - initial approach	II, III, VI, VII	**Length of hospital stay<br>Time to urinary catheter removal**	**JBI Critical Appraisal Checklist for Cohort Studies (Q11)**	**Y**	**N/A**	**Y**	**N/A**	**N/A**	**Y**	**Y**	**Y**	**Y**	**N/A**	**Y**		
Anthony Cometa M[7] (2018)	Case Report	n - 1<br>placenta accreta	extirpative	emergency cesarean hysterectomy	II, III, IV, VI, VII	**Length of hospital stay<br>Apgar scale**	**JBI Critical Appraisal Checklist for Case Reports (Q8)**	**Y**	**Y**	**Y**	**Y**	**Y**	**N**	**N/A**	**Y**					
Bamidele B[8] (2018)	Case Report	n - 1<br>placenta accreta	extirpative	elective cesarean delivery with uterine preservation	II, IV, V, VI	**Timing of extubation**	**JBI Critical Appraisal Checklist for Case Reports (Q8)**	**Y**	**Y**	**N**	**N**	**Y**	**N**	**N/A**	**Y**					
Batra J[9] (2024)	Case Report	n – 1<br>placenta percreta	extirpative	elective cesarean delivery with anticipated hysterectomy	I, II, III, IV, VI, VII	**Mechanical ventilation/sedation time<br>Timing of extubation<br>Breastfeeding by time of discharge**	**JBI Critical Appraisal Checklist for Case Reports (Q8)**	**y**	**y**	**y**	**y**	**y**	**U**	**N/A**	**y**					
Bell-Thomas SM[10] (2003)	Case Report	n - 1<br>placenta percreta	extirpative<br>IIABO<br>Postdelivery cystoscopy	elective cesarean delivery with uterine preservation	I, II, V, VI, VII	**Mechanical ventilation/sedation time<br>Timing of extubation<br>Time to urinary catheter removal**	**JBI Critical Appraisal Checklist for Case Reports (Q8)**	**Y**	**U**	**N**	**N**	**Y**	**U**	**Y**	**Y**					
Bergakker SA[11] (2010)	Case Report	n - 1<br>placenta accreta	Conservative	elective cesarean delivery with uterine preservation	II, III, IV, VI, VII	**Postoperative opioid use<br>Timing of extubation<br>Time to urinary catheter removal**	**JBI Critical Appraisal Checklist for Case Reports (Q8)**	**Y**	**Y**	**Y**	**Y**	**N**	**Y**	**Y**	**Y**					
Bhavani-Shankar K[12] (2000)	Case Report	n - 1<br>not specified – suggestive of percreta	Extirpative	elective cesarean delivery with emergency hysterectomy	II, IV, V, VII	**Time to urinary catheter removal**	**JBI Critical Appraisal Checklist for Case Reports (Q8)**	**Y**	**Y**	**N**	**Y**	**N**	**N**	**U**	**Y**					
Biele C[13] (2021)	Case Report	n - 1<br>placenta percreta	Conservative	elective cesarean delivery with uterine preservation	II, V, VI, VII	**Length of hospital stay<br>Time to urinary catheter removal<br>Apgar scale**	**JBI Critical Appraisal Checklist for Case Reports (Q8)**	**Y**	**Y**	**Y**	**Y**	**Y**	**Y**	**Y**	**Y**					
Biji KP[14] (2021)	Case Series	n - 2<br>placenta percreta	extirpative	elective cesarean hysterectomy	II, III, IV, V, VI, VII	**Mechanical ventilation/sedation time<br>Timing of extubation**	**JBI Critical Appraisal Checklist for Case Series (Q10)**	**U**	**Y**	**Y**	**N**	**N**	**Y**	**Y**	**Y**	**N**	**N/A**			
Binici O[15] (2019)	Cohort Study	n - 92<br>placenta percreta - 42<br>placenta increta - 1	not specified	not specified	II, IV, V, VI, VII	**Apgar scale**	**JBI Critical Appraisal Checklist for Cohort Studies (Q11)**	**Y**	**Y**	**N/A**	**N/A**	**N/A**	**N/A**	**Y**	**Y**	**Y**	**N/A**	**Y**		
Busani S[16] (2015)	Case Report	n - 1<br>not specified – suggestive of increta	extirpative	elective cesarean hysterectomy	I, II, III, IV, V, VI, VII	**Length of hospital stay<br>Mechanical ventilation/sedation time<br>Timing of extubation**	**JBI Critical Appraisal Checklist for Case Reports (Q8)**	**Y**	**Y**	**Y**	**Y**	**Y**	**Y**	**N/A**	**Y**					

Butwick AJ[17] (2011)	Case Series	n - 5<br>placenta accreta - 3<br>placenta percreta - 2	extirpative<br>preoperative cystoscopy	elective cesarean hysterectomy	II, III, IV, V, VI, VII	**Length of hospital stay<br>Mechanical ventilation/sedation time<br>Timing of extubation<br>Apgar scale**	**JBI Critical Appraisal Checklist for Case Series (Q10)**	**Y**	**Y**	**Y**	**N**	**U**	**N/A**	**N/A**	**Y**	**Y**	**Y**			
Buyukkurt S[18] (2023)	Cohort Study	n – 161<br>placenta increta<br>placenta percreta	extirpative	elective or emergency cesarean hysterectomy<br>elective or emergency cesarean delivery with anticipated hysterectomy	II, V, VI, VII	**Length of hospital stay<br>Time to first fluid intake postoperatively<br>Time to first solid food intake postoperatively<br>Time to first mobilization<br>Apgar scale**	**JBI Critical Appraisal Checklist for Cohort Studies (Q11)**	**y**	**y**	**y**	**N/A**	**N/A**	**N/A**	**y**	**y**	**y**	**N/A**	**Y**		
Chao WT[19] (2022)	Case Series	n - 5<br>placenta accreta - 3<br>placenta increta - 2	conservative<br>IIABO, UAE	elective cesarean delivery with uterine preservation	I, II, III, IV, V, VI, VII	**Length of hospital stay<br>Postoperative opioid use<br>Timing of extubation<br>Apgar scale**	**JBI Critical Appraisal Checklist for Case Series (Q10)**	**Y**	**Y**	**Y**	**Y**	**Y**	**Y**	**Y**	**Y**	**N/A**	**Y**			
Chavez R[20] (2017)	Case Study	n - 1<br>placenta increta	extirpative<br>UABO	elective cesarean hysterectomy	II		**JBI Critical Appraisal Checklist for Case Reports (Q8)**	**Y**	**Y**	**Y**	**Y**	**Y**	**N**	**N**	**U**					
Churchill S[21] (2018)	Case Report – conference abstract	n - 1<br>placenta percreta	extirpative	elective cesarean delivery with anticipated hysterectomy	I, II, V, VI		**Critical Appraisal Checklist of conference abstracts, letters to the editor and correspondence papers (Q6)**	**Y**	**Y**	**N/A**	**N/A**	**Y**	**N/A**							
Clapham E[22] (2018)	Quasi experimental Study – conference abstract	n - 31<br>PAS - not specified	not specified	not specified	II, III, IV, V, VII	**Length of hospital stay**	**Critical Appraisal Checklist of conference abstracts, letters to the editor and correspondence papers (Q6)**	**Y**	**Y**	**Y**	**Y**	**Y**	**Y**							
Dayican HY[23] (2023)	Case Series - conference abstract	n – 8<br>placenta percreta	not specified	not specified	II, III, IV, V, VI		**Critical Appraisal Checklist of conference abstracts, letters to the editor and correspondence papers (Q6)**	**y**	**y**	**y**	**y**	**y**	**N/A**							
Dhansura T[24] (2015)	Case Report	n - 1<br>placenta percreta	conservative<br>IIABO + IIAE	elective cesarean delivery with uterine preservation	II, III, VI	**Length of hospital stay<br>Timing of extubation**	**JBI Critical Appraisal Checklist for Case Reports (Q8)**	**Y**	**Y**	**N**	**N**	**Y**	**N**	**N/A**	**Y**					
Dogra S[25] (2023)	Case Report	n – 2<br>placenta percreta	extirpative	elective and emergency cesarean hysterectomy	I, II, III, IV, V, VI, VII	**Mechanical ventilation/sedation time<br>Apgar scale**	**JBI Critical Appraisal Checklist for Case Reports (Q8)**	**y**	**y**	**U**	**N**	**Y**	**N**	**Y**	**Y**					
Dolling S[26] (2022)	Case Series - conference abstract	n - 61<br>PAS - not specifed	conservative and extirpative<br>IIABO	elective and emergency not specified	II, V		**Critical Appraisal Checklist of conference abstracts, letters to the editor and correspondence papers (Q6)**	**Y**	**Y**	**N/A**	**Y**	**Y**	**N/A**							
Elagamy A[27] (2013)	Case Series	n - 20<br>PAS - not specified	conservative and extirpative	elective cesarean delivery with anticipated hysterectomy	II, III, V, VI	**Length of hospital stay**	**JBI Critical Appraisal Checklist for Case Series (Q10)**	**Y**	**Y**	**Y**	**N**	**N**	**Y**	**Y**	**Y**	**N**	**Y**			

Estella NM[28] (1997)	Case Report	n - 1<br>placenta percreta	Extirpative<br>preoperative cystoscopy	elective cesarean hysterectomy	II, IV, VII	**Length of hospital stay<br>Apgar scale**	**JBI Critical Appraisal Checklist for Case Reports (Q8)**	**Y**	**Y**	**Y**	**Y**	**Y**	**Y**	**N/A**	**Y**					
Faris AS[29] (2014)	Case Report	n - 1<br>placenta percreta	extirpative	elective cesarean hysterectomy	I, II, III, V, VI, VII	**Length of hospital stay<br>Mechanical ventilation/sedation time<br>Timing of extubation<br>Apgar scale<br>pICU admission**	**JBI Critical Appraisal Checklist for Case Reports (Q8)**	**Y**	**Y**	**Y**	**Y**	**Y**	**N**	**N/A**	**U**					
Feldman JB[30] (2019)	Case Report	n - 1<br>placenta percreta	Extirpative<br>preoperative cystoscopy	elective cesarean delivery with anticipated hysterectomy	I, II, IV, V, VI, VII	**Length of hospital stay<br>Mechanical ventilation/sedation time<br>Timing of extubation<br>Apgar scale**	**JBI Critical Appraisal Checklist for Case Reports (Q8)**	**Y**	**Y**	**Y**	**Y**	**Y**	**Y**	**Y**	**Y**					
Feng S[31] (2017)	Quasiexperimental Study	n - 41<br>placenta accreta - 14<br>placenta increta - 22<br>placenta percreta - 5	conservative and extirpative<br>IIABO	elective cesarean delivery with anticipated hysterectomy	II, V, VI, VII	**Length of hospital stay<br>Apgar scale<br>pICU admission**	**JBI Critical Appraisal Checklist for Quasiexperimental Studies (Q9)**	**Y**	**Y**	**Y**	**Y**	**N**	**N/A**	**Y**	**Y**	**Y**				
Frasca D[32] (2012)	Case Report	n - 1<br>placenta percreta	extirpative	emergency cesarean hysterectomy	I, II, IV, V, VI, VII	**Length of hospital stay<br>Mechanical ventilation/sedation time<br>Timing of extubation**	**JBI Critical Appraisal Checklist for Case Reports (Q8)**	**Y**	**Y**	**Y**	**Y**	**Y**	**N**	**N/A**	**Y**					
Gatta LA[33] (2022)	Cohort Study	n - 87<br>placenta accreta - 32<br>placenta increta - 39<br>placenta percreta - 15	extirpative<br>UAE	elective and emergency cesarean hysterectomy – early or delayed	I, II, V, VI, VII		**JBI Critical Appraisal Checklist for Cohort Studies (Q11)**	**Y**	**N/A**	**N/A**	**N/A**	**N/A**	**Y**	**Y**	**Y**	**N/A**	**N/A**	**Y**		
Glynn JC[34] (2007)	Case Report - correspondence	n - 1<br>placenta accreta	extirpative	not specified	II, V		**Critical Appraisal Checklist of conference abstracts, letters to the editor and correspondence papers (Q6)**	**Y**	**Y**	**N/A**	**N/A**	**Y**	**N/A**							
Günaydın B[35] (2016)	Case Report	n - 1<br>placenta increta	extirpative	emergency cesarean hysterectomy	II, III, IV, V, VI, VII	**Length of hospital stay<br>Apgar scale**	**JBI Critical Appraisal Checklist for Case Reports (Q8)**	**Y**	**Y**	**Y**	**Y**	**Y**	**N**	**N**	**Y**					
Hajmurad OS[36] (2017)	Case Report	n - 1	extirpative<br>IIABO	emergency cesarean hysterectomy	II, IV, V, VI	**Length of hospital stay**	**JBI Critical Appraisal Checklist for Case Reports (Q8)**	**Y**	**Y**	**Y**	**Y**	**Y**	**Y**	**Y**	**Y**					
Hania A[37] (2022)	Case Series	n - 9	extirpative<br>IIABO	elective cesarean delivery with anticipated hysterectomy	I, II, V, VI	**Length of hospital stay**	**JBI Critical Appraisal Checklist for Case Series (Q10)**	**Y**	**U**	**Y**	**Y**	**U**	**Y**	**Y**	**N**	**N/A**	**Y**			
Hegde HV[38] (2024)	Case Series	n - 216<br>PAS not specified	Conservative and extirpative<br>IIABO	elective cesarean delivery with anticipated hysterectomy<br>emergency cesarean hysterectomy	II, III, IV, V, VI, VII	**Length of hospital stay<br>Mechanical ventilation/sedation time<br>Timing of extubation<br>Postoperative vasopressor use<br>Apgar scale<br>Postoperative opioid use**	**JBI Critical Appraisal Checklist for Case Series (Q10)**	**y**	**y**	**y**	**y**	**y**	**y**	**y**	**y**	**N/A**	**y**			

Herbert KA[39] (2022)	Cohort Study	n - 113<br>placenta accreta - 33<br>placenta increta - 47<br>placenta percreta - 25	extirpative<br>IIABO + UAE	elective cesarean hysterectomy – early or delayed<br>elective cesarean delivery with emergency hysterectomy<br>emergency cesarean hysterectomy	I, II, IV, VI, VII	**Length of hospital stay<br>Maternal hospital readmission rate<br>Postoperative opioid use<br>Postoperative opioid consumption**	**JBI Critical Appraisal Checklist for Cohort Studies (Q11)**	**Y**	**Y**	**Y**	**Y**	**Y**	**Y**	**Y**	**Y**	**Y**	**N/A**	**Y**		
Humphrey J[40] (2015)	Case Report	n - 1<br>placenta increta - 1	Extirpative<br>postoperative cystoscopy	elective cesarean hysterectomy	II, III, IV, V, VII	**Length of hospital stay<br>Mechanical ventilation/sedation time<br>Timing of extubation<br>Apgar scale**	**JBI Critical Appraisal Checklist for Case Reports (Q8)**	**Y**	**Y**	**Y**	**Y**	**Y**	**Y**	**Y**	**Y**					
Hunter T[41] (1996)	Case Report	n - 1<br>placenta percreta	Extirpative<br>preoperative cystoscopy	elective cesarean hysterectomy	II, III, IV, V, VI, VII	**Timing of extubation<br>Time to urinary catheter removal<br>Apgar scale**	**JBI Critical Appraisal Checklist for Case Reports (Q8)**	**Y**	**Y**	**N/A**	**Y**	**Y**	**Y**	**N/A**	**Y**					
Ioscovich A[42] (2009)	Case Series	n – 108 (at risk of PAS)<br>PAS not specified	conservative and extirpative	elective and emergency cesarean delivery with anticipated hysterectomy	I, II, III, V, VI, VII	**Apgar scale**	**JBI Critical Appraisal Checklist for Case Series (Q10)**	**Y**	**Y**	**Y**	**Y**	**Y**	**Y**	**Y**	**Y**	**N/A**	**Y**			
Ismail S[43] (2019)	Case Report	n - 1<br>placenta percreta	extirpative	elective cesarean delivery with anticipated hysterectomy	I, II, III, IV, V, VI, VII	**Length of hospital stay<br>Postoperative opioid use<br>Timing of extubation<br>pICU admission**	**JBI Critical Appraisal Checklist for Case Reports (Q8)**	**Y**	**Y**	**Y**	**Y**	**Y**	**U**	**N/A**	**Y**					
Jheng-Yan Lan[44] (2011)	Case Series	n - 19<br>placenta accreta/percreta - not specified	extirpative<br>IIABO	elective cesarean hysterectomy	I, II, VII	**Apgar scale**	**JBI Critical Appraisal Checklist for Case Series (Q10)**	**Y**	**U**	**U**	**U**	**U**	**Y**	**Y**	**Y**	**N/A**	**Y**			
Ji SM[45] (2020)	Case Report	n - 1<br>placenta percreta	extirpative<br>REBOA	emergency cesarean hysterectomy	I, II, V, VI, VII	**Length of hospital stay<br>Mechanical ventilation/sedation time<br>Timing of extubation<br>Apgar scale**	**JBI Critical Appraisal Checklist for Case Reports (Q8)**	**Y**	**Y**	**Y**	**N/A**	**Y**	**Y**	**N/A**	**Y**					
Jiang X[46] (2019)	Case Report	n - 1<br>placenta accreta	extirpative	emergency cesarean hysterectomy	I, II, III, IV, V, VII	**Length of hospital stay<br>Apgar scale**	**JBI Critical Appraisal Checklist for Case Reports (Q8)**	**Y**	**Y**	**Y**	**Y**	**Y**	**N**	**Y**	**Y**					
Kamani AA[47] (1987)	Case Report	n - 1<br>placenta accreta	extirpative	emergency cesarean hysterectomy	II, III, IV, V, VI		**JBI Critical Appraisal Checklist for Case Reports (Q8)**	**Y**	**Y**	**Y**	**N/A**	**Y**	**N**	**N/A**	**Y**					
Karacaer F[48] (2018)	Case Series	n - 89<br>placenta accreta - 85<br>placenta percreta - 4	conservative and extirpative<br>preoperative cystoscopy	elective and emergency cesarean hysterectomy<br>elective and emergency cesarean delivery with uterine preservation	I, II, III, IV, V, VI, VII	**Length of hospital stay<br>Postoperative opioid use<br>Timing of extubation<br>Postoperative vasopressor use<br>Apgar scale<br>pICU admission**	**JBI Critical Appraisal Checklist for Case Series (Q10)**	**Y**	**Y**	**Y**	**U**	**U**	**Y**	**Y**	**Y**	**Y**	**Y**			
Kessack LK[49] (2010)	Case Report	n - 1<br>placenta accreta	extirpative<br>IIABO	elective cesarean delivery with anticipated hysterectomy	II, III, IV, V, VII	**Length of hospital stay**	**JBI Critical Appraisal Checklist for Case Reports (Q8)**	**Y**	**Y**	**Y**	**Y**	**Y**	**N**	**Y**	**Y**					
Khokhar RS[50] (2016)	Case Report	n - 1<br>placenta accreta	Extirpative<br>preoperative cystoscopy	elective cesarean hysterectomy	I, II, III, IV, V, VI	**Postoperative opioid use<br>Timing of extubation<br>Apgar scale**	**JBI Critical Appraisal Checklist for Case Reports (Q8)**	**Y**	**Y**	**Y**	**Y**	**Y**	**N**	**N/A**	**N**					

Kocaoglu N[51] (2012)	Cohort Study	n – 65 (total), 28 - PAS<br>placenta accreta – 12<br>placenta increta – 8<br>placenta percreta - 8	conservative and extirpative	elective and emergency cesarean delivery with anticipated hysterectomy	II, V, VI, VII	**Length of hospital stay**	**JBI Critical Appraisal Checklist for Cohort Studies (Q11)**	**Y**	**Y**	**Y**	**N/A**	**N/A**	**Y**	**Y**	**Y**	**Y**	**N/A**	**Y**		
Konishi Y[52] (2016)	Case Report	n - 1<br>placenta percreta	extirpative<br>UAE<br>preoperative cystoscopy	elective staged delivery with delayed hysterectomy	II, IV, V, VI, VII	**Length of hospital stay**<br>**Postoperative opioid use**<br>**Apgar scale**	**JBI Critical Appraisal Checklist for Case Reports (Q8)**	**Y**	**Y**	**Y**	**N/A**	**Y**	**N**	**N/A**	**Y**					
Korolev A (2019)[53]	Case Series	n - 3<br>placenta increta	conservative and extirpative	elective cesarean delivery with uterine preservation<br>emergency cesarean hysterectomy	I, II, IV, V, VI, VII	**Length of hospital stay**<br>**Postoperative opioid use**<br>**Timing of extubation**<br>**Time to first mobilization**<br>**Apgar scale**	**JBI Critical Appraisal Checklist for Case Series (Q10)**	**Y**	**Y**	**Y**	**Y**	**Y**	**Y**	**N/A**	**Y**					
Kuczkowski KM[54] (2006)	Case Report	n - 1<br>PAS not specified	extirpative<br>IIABO	elective cesarean delivery with anticipated hysterectomy	II, VI, VII	**Length of hospital stay**<br>**Apgar scale**	**JBI Critical Appraisal Checklist for Case Reports (Q8)**	**Y**	**Y**	**Y**	**Y**	**Y**	**N**	**N/A**	**Y**					
Kume K[55] (2014)	Case Report	n - 1<br>placenta percreta	extirpative<br>IIAE	emergency cesarean hysterectomy	II, IV, V, VI, VII	**Length of hospital stay**<br>**Mechanical ventilation/sedation time**<br>**Timing of extubation**<br>**Apgar scale**	**JBI Critical Appraisal Checklist for Case Reports (Q8)**	**Y**	**Y**	**N**	**Y**	**Y**	**Y**	**N/A**	**Y**					
Laranjo M[56] (2024)	Case Report	n – 1<br>placenta accreta	extirpative	emergency cesarean hysterectomy	II, III, IV, V, VI, VII	**Length of hospital stay**<br>**Timing of extubation**<br>**Postoperative vasopressor use**<br>**Apgar scale**<br>**pICU admission**	**JBI Critical Appraisal Checklist for Case Reports (Q8)**	**y**	**y**	**y**	**N/A**	**y**	**y**	**N/A**	**y**					
Lekic Z[57] (2017)	Quasiexperimental Study	n - 19<br>PAS type not specified<br>- 1x placenta percreta	extirpative<br>preoperative cystoscopy (?)	elective and emergency cesarean hysterectomy	I, II, III, V, VI, VII	**Length of hospital stay**	**JBI Critical Appraisal Checklist for Quasiexperimental Studies (Q9)**	**Y**	**Y**	**Y**	**Y**	**N**	**Y**	**Y**	**Y**	**Y**				
Li P[58] (2020)	Cohort Study	n - 57<br>placenta accreta - 15<br>placenta increta - 12<br>placenta percreta - 11	conservative and extirpative	elective cesarean delivery with anticipated hysterectomy	I, II, IV, V, VI, VII	**Length of hospital stay**<br>**Mechanical ventilation/sedation time**<br>**Timing of extubation**<br>**Apgar scale**	**JBI Critical Appraisal Checklist for Cohort Studies (Q11)**	**Y**	**Y**	**Y**	**Y**	**Y**	**Y**	**Y**	**Y**	**Y**	**N/A**	**Y**		
Lilker SJ[59] (2011)	Case Series	n - 23<br>unclear detailed diagnosis - placenta accreta	conservative<br>UABO, UAE	elective cesarean delivery with uterine preservation	I, II, V, VI, VII	**Maternal hospital readmission rate**<br>**Mechanical ventilation/sedation time**<br>**Postoperative opioid use**<br>**Timing of extubation**	**JBI Critical Appraisal Checklist for Case Series (Q10)**	**Y**	**Y**	**Y**	**Y**	**Y**	**Y**	**Y**	**Y**	**Y**	**Y**			
Liu C[60] (2024)	Cohort Study	n - 425<br>placenta accreta - 7<br>placenta increta - 370<br>placenta percreta - 48	conservative and extirpative	elective cesarean delivery with anticipated hysterectomy	II, V, VI, VII	**Length of hospital stay**<br>**Apgar scale**<br>**pICU admission**	**JBI Critical Appraisal Checklist for Cohort Studies (Q11)**	**y**	**y**	**y**	**y**	**N/A Y**	**y**	**y**	**y**	**N/A**	**y**			

Lopez-Erazo LJ[61] (2021)	Quasiexperimental Study	n - 81<br>placenta accreta or increta - 46<br>placenta percreta - 13	conservative and extirpative<br>IIABO,<br>REBOA<br>preoperative cystoscopy	elective and emergency cesarean delivery with anticipated hysterectomy	I, II, III, IV, V, VII	**Length of hospital stay**	**JBI Critical Appraisal Checklist for Quasiexperimental Studies (Q9)**	**Y**	**Y**	**Y**	**Y**	**N/A**	**Y**	**Y**	**Y**	**Y**				
Loreto M[62] (2022)	Quasiexperimental Study	n - 40<br>unclear detailed diagnosis - placenta accreta	conservative and extirpative	elective cesarean delivery with anticipated hysterectomy	I, II, III, IV, V, VI	**Length of hospital stay**<br>**Maternal hospital readmission rate**	**JBI Critical Appraisal Checklist for Quasiexperimental Studies (Q9)**	**Y**	**U**	**Y**	**Y**	**Y**	**N/A**	**Y**	**Y**	**Y**				
Ma Y[63] (2019)	Case Report	n - 1<br>placenta accreta	conservative<br>IIABO	elective cesarean delivery with uterine preservation	II, III V, VII	**Apgar scale**	**JBI Critical Appraisal Checklist for Case Reports (Q8)**	**Y**	**Y**	**Y**	**Y**	**Y**	**N**	**N/A**	**Y**					
Ma Y[64] (2020)	Case Report	n - 1<br>placenta accreta	conservative	elective cesarean delivery with uterine preservation	II, IV, V, VII	**Length of hospital stay**<br>**Apgar scale**	**JBI Critical Appraisal Checklist for Case Reports (Q8)**	**Y**	**Y**	**Y**	**Y**	**Y**	**U**	**N/A**	**Y**					
Malik Z[65] (2017)	Case Series - conference abstract	n - 27<br>placenta accreta - 18<br>placenta percreta - 9	conservative<br>IIABO	elective cesarean delivery with uterine preservation	II, III V, VI, VII	**Length of hospital stay**	**Critical Appraisal Checklist of conference abstracts, letters to the editor and correspondence papers (Q6)**	**Y**	**Y**	**N/A**	**Y**	**Y**	**N/A**							
Mansouri M[66] (2017)	Cohort Study	n - 28<br>PAS type not specified	extirpative<br>preoperative cystoscopy	elective cesarean hysterectomy	II, IV, V, VII	**Length of hospital stay**	**JBI Critical Appraisal Checklist for Cohort Studies (Q11)**	**U**	**U**	**U**	**N/A**	**N/A**	**Y**	**Y**	**Y**	**N/A**	**N/A**	**Y**		
Markley JC[67] (2018)	Cohort Study	n – 129 (total), 81 - PAS<br>placenta accreta – 23<br>placenta increta – 31<br>placenta percreta - 27	conservative and extirpative<br>unspecified method of interventional radiology<br>preoperative cystoscopy	elective cesarean delivery with anticipated hysterectomy	II, III, IV, VI, VII	**Length of hospital stay**<br>**Maternal hospital readmission rate**<br>**Apgar scale**	**JBI Critical Appraisal Checklist for Cohort Studies (Q11)**	**Y**	**Y**	**Y**	**Y**	**Y**	**Y**	**Y**	**Y**	**Y**	**N/A**	**Y**		
Mauritz AA[68] (2016)	Case Report	n - 1<br>placenta percreta	extirpative<br>UAE	elective staged delivery with delayed hysterectomy	I, II, III, IV, V, VII	**Length of hospital stay**<br>**Apgar scale**	**JBI Critical Appraisal Checklist for Case Reports (Q8)**	**Y**	**U**	**Y**	**Y**	**Y**	**Y**	**N/A**	**Y**					
Mok M[69] (2008)	Case Series	n - 13<br>placenta accreta - 3<br>placenta percreta - 2	extirpative<br>IIABO	elective and emergency cesarean hysterectomy	I, II, III, V, VI		**JBI Critical Appraisal Checklist for Case Series (Q10)**	**Y**	**Y**	**Y**	**Y**	**Y**	**Y**	**Y**	**Y**	**N/A**	**Y**			
Morland D[70] (2009)	Case Report - conference abstract	n - 1<br>placenta accreta - suspicion of percreta	extirpative<br>IIABO	elective cesarean hysterectomy	II, III, IV, VII	**Length of hospital stay**	**Critical Appraisal Checklist of conference abstracts, letters to the editor and correspondence papers (Q6)**	**Y**	**Y**	**Y**	**N/A**	**Y**	**N/A**							

Munoz LA[71] (2015)	Case Series	n - 40<br>placenta accreta - 16<br>placenta increta - 19<br>placenta percreta - 5	extirpative	elective cesarean hysterectomy	I, II, III, IV, V, VI, VII	**Length of hospital stay**<br>**Postoperative vasopressor use**<br>**Apgar scale**	**JBI Critical Appraisal Checklist for Case Series (Q10)**	**Y**	**Y**	**Y**	**Y**	**N**	**Y**	**Y**	**Y**	**N/A**	**Y**			
Nagy CJ[72] (2008)	Case Report	n - 1<br>placenta percreta	extirpative	elective cesarean hysterectomy	II, III, IV, V, VI, VII	**Length of hospital stay**<br>**Timing of extubation**<br>**Apgar scale**	**JBI Critical Appraisal Checklist for Case Reports (Q8)**	**Y**	**Y**	**Y**	**Y**	**Y**	**Y**	**N/A**	**Y**					
Nanji JA[73] (2019)	Case Report	n - 1<br>placenta percreta	extirpative	elective cesarean hysterectomy	I, II, III, IV, V, VI		**JBI Critical Appraisal Checklist for Case Reports (Q8)**	**Y**	**Y**	**Y**	**Y**	**Y**	**N**	**Y**	**Y**					
Neef V[74] (2024)	Cohort Study	n – 17<br>placenta increta – 1<br>placenta percreta - 16	conservative and extirpative<br>UAE	elective staged delivery with anticipated delayed hysterectomy	II, III, V, VI, VII	**Length of hospital stay**	**JBI Critical Appraisal Checklist for Cohort Studies (Q11)**	**y**	**y**	**y**	**N/A**	**N/A**	**N/A**	**y**	**y**	**y**	**N/A**	**Y**		
Nguyen-Lu N[75] (2016)	Cohort Study	n - 50<br>placenta accreta - 11<br>placenta increta - 15<br>placenta percreta - 24	conservative and extirpative<br>IIABO, UAE	elective and emergency cesarean delivery with anticipated hysterectomy	II, III, IV, V, VI, VII	**Length of hospital stay**<br>**Maternal hospital readmission rate**<br>**Mechanical ventilation/sedation time**<br>**Postoperative opioid use**<br>**Timing of extubation**<br>**Apgar scale**	**JBI Critical Appraisal Checklist for Cohort Studies (Q11)**	**Y**	**Y**	**Y**	**N/A**	**N/A**	**N/A**	**Y**	**Y**	**Y**	**N/A**	**Y**		
Nieto AJ[76] (2020)	Quasiexperimental Study	n - 62<br>placenta accreta - 31<br>placenta increta - 8<br>placenta percreta - 9	conservative and extirpative<br>IAABO, IIABO<br>preoperative cystoscopy	elective and emergency cesarean hysterectomy<br>elective cesarean delivery with anticipated hysterectomy	I, II, III, IV, V, VI, VII	**Length of hospital stay**<br>**pICU admission**	**JBI Critical Appraisal Checklist for Quasiexperimental Studies (Q9)**	**Y**	**Y**	**Y**	**Y**	**N/A**	**N/A**	**Y**	**Y**	**Y**				
Nieto-Calvache AJ[77] (2021)	Quasiexperimental Study-conference abstract	n - 81<br>placenta percreta - 13<br>remainder not specified	conservative and extirpative	elective cesarean delivery with anticipated hysterectomy	II, V, VI, VII	**Length of hospital stay**	**Critical Appraisal Checklist of conference abstracts, letters to the editor and correspondence papers (Q6)**	**Y**	**Y**	**Y**	**Y**	**Y**	**Y**							
Nieto-Calvache AJ[78] (2022)	Quasiexperimental Study	n - 66<br>placenta accreta - 35<br>placenta increta -6<br>placenta percreta - 15	conservative and extirpative<br>IAABO, IIABO	elective and emergency cesarean delivery with anticipated hysterectomy	I, V, VI		**JBI Critical Appraisal Checklist for Quasiexperimental Studies (Q9)**	**Y**	**U**	**Y**	**Y**	**N/A**	**Y**	**Y**	**Y**	**Y**				
Ohsugi E[79] (2023)	Case Report	n – 1<br>placenta increta	extirpative<br>IAABO	emergency cesarean hysterectomy	II, III, IV, V, VI, VII	**Length of hospital stay**<br>**Apgar scale**<br>**pICU admission**	**JBI Critical Appraisal Checklist for Case Reports (Q8)**	**y**	**y**	**y**	**N/A**	**N/A**	**Y**	**Y**	**Y**					
Omowanile YA[80] (2017)	Case Report	n - 1<br>placenta percreta	extirpative<br>IIABO	elective cesarean hysterectomy	I, II, IV, V		**JBI Critical Appraisal Checklist for Case Reports (Q8)**	**Y**	**Y**	**Y**	**Y**	**Y**	**Y**	**N/A**	**Y**					

Orr T[81] (2018)	Case Report - conference abstract	n - 1<br>placenta percreta.	extirpative	emergency cesarean hysterectomy	II, III, V, VI, VII	**Mechanical ventilation/sedation time**<br>**Timing of extubation**<br>**Apgar scale**	**Critical Appraisal Checklist of conference abstracts, letters to the editor and correspondence papers (Q6)**	**Y**	**Y**	**Y**	**N/A**	**Y**	**N/A**							
Otero CR[82] (2021)	Case Report	n - 1<br>placenta increta	extirpative<br>REBOA	elective cesarean hysterectomy	I, II, III, IV, V, VII	**Timing of extubation**<br>**Apgar scale**	**JBI Critical Appraisal Checklist for Case Reports (Q8)**	**N**	**Y**	**N**	**N**	**Y**	**N**	**N/A**	**Y**					
Oygen O[83] (2024)	Cohort Study	n – 631<br>PAS not specified	conservative and extirpative	elective and emergency initial approach not specified	II, IV, V, VI		**JBI Critical Appraisal Checklist for Cohort Studies (Q11)**	**Y**	**N**	**N**	**N/A**	**N/A**	**Y**	**Y**	**Y**	**N/A**	**N/A**	**Y**		
Pandey M[84] (2024)	Case Report	n - 1<br>placenta accreta - 1	conservative<br>IIABO	elective cesarean delivery with anticipated hysterectomy	II, III, V, VI, VII	**Length of hospital stay**	**JBI Critical Appraisal Checklist for Case Reports (Q8)**	**Y**	**Y**	**Y**	**Y**	**Y**	**Y**	**N/A**	**Y**					
Panigrahi AK[85] (2017)	Cohort Study	n - 136<br>placenta accreta - 60<br>placenta increta - 17<br>placenta percreta - 17	conservative and extirpative<br>UABO	elective cesarean delivery with anticipated hysterectomy	I, II, V		**JBI Critical Appraisal Checklist for Cohort Studies (Q11)**	**Y**	**Y**	**Y**	**N/A**	**N/A**	**Y**	**Y**	**Y**	**N/A**	**N/A**	**Y**		
Panjeton GD[86] (2022)	Cohort Study	n - 39<br>placenta accreta - 9<br>placenta increta - 5<br>placenta percreta - 6	conservative and extirpative	elective cesarean delivery with anticipated hysterectomy	II, III, IV, V, VI, VII	**Length of hospital stay**<br>**Postoperative opioid use**<br>**Postoperative opioid consumption**<br>**Apgar scale**<br>**pICU admission**	**JBI Critical Appraisal Checklist for Cohort Studies (Q11)**	**Y**	**Y**	**Y**	**N/A**	**N/A**	**Y**	**Y**	**Y**	**Y**	**N/A**	**Y**		
Pareek S[87] (2024)	Case Report	n - 1<br>placenta increta - 1	extirpative	elective cesarean delivery with anticipated hysterectomy	I, II, III, IV, V, VI, VII	**Timing of extubation**<br>**Apgar scale**	**JBI Critical Appraisal Checklist for Case Reports (Q8)**	**Y**	**Y**	**N/A**	**Y**	**Y**	**Y**	**N/A**	**Y**					
Patil Y[88] (2023)	Case Series	n - 14<br>placenta accreta - 7<br>placenta increta - 1<br>placenta percreta - 2	conservative and extirpative<br>IIABO<br>UAE	elective cesarean delivery with anticipated hysterectomy	II, V, VI		**JBI Critical Appraisal Checklist for Case Series (Q10)**	**Y**	**Y**	**Y**	**U**	**U**	**Y**	**Y**	**N/A**	**N/A**	**Y**			
Parva M[89] (2010)	Case Report	n - 1<br>placenta percreta	extirpative<br>IIABO	elective cesarean hysterectomy	I, II, IV, V, VI, VII	**Length of hospital stay**<br>**Timing of extubation**<br>**Time to urinary catheter removal**<br>**Apgar scale**	**JBI Critical Appraisal Checklist for Case Reports (Q8)**	**Y**	**Y**	**Y**	**Y**	**Y**	**Y**	**Y**	**Y**					
Paull JD[90] (1995)	Case Report	n - 1<br>placenta percreta	extirpative<br>IAABO	elective cesarean hysterectomy	II, IV, V, VI, VII	**Length of hospital stay**<br>**Postoperative opioid use**<br>**Timing of extubation**<br>**Apgar scale**	**JBI Critical Appraisal Checklist for Case Reports (Q8)**	**Y**	**Y**	**Y**	**Y**	**Y**	**Y**	**N/A**	**Y**					

Pini R[91] (2023)	Case Report – letter to the editor	n - 1<br>PAS not specified	extirpative<br>REBOA	emergency cesarean hysterectomy	II, III, IV, V		**Critical Appraisal Checklist of conference abstracts, letters to the editor and correspondence papers (Q6)**	**Y**	**Y**	**Y**	**Y**	**Y**	**Y**							
Plakhotina EN[92] (2020)	RCT	n - 80<br>placenta accreta (?)	conservative	elective cesarean delivery with uterine preservation	II, III, IV, V, VI, VII	**Length of hospital stay<br>Postoperative opioid use<br>Timing of extubation<br>Maternal satisfaction with analgesia<br>Apgar scale**	**JBI Critical Appraisal Checklist for Randomized Controlled Trials (Q13)**	**U**	**U**	**U**	**N**	**N**	**N**	**Y**	**N/A**	**Y**	**Y**	**U**	**N**	**N**
Rana S[93] (2020)	Cohort Study	n - 109<br>PAS not specified	conservative and extirpative	elective and emergency cesarean hysterectomy<br>elective cesarean delivery with anticipated hysterectomy	I, II, V, VI, VII	**Length of hospital stay<br>Timing of extubation**	**JBI Critical Appraisal Checklist for Cohort Studies (Q11)**	**Y**	**Y**	**U**	**N/A**	**N/A**	**Y**	**Y**	**Y**	**Y**	**N/A**	**Y**		
Riveros-Perez E[94] (2018)	Case Series	n - 43<br>placenta accreta - 25<br>placenta increta - 8<br>placenta percreta - 10	conservative and extirpative<br>IIABO<br>preoperative cystoscopy	elective and emergency cesarean delivery with anticipated hysterectomy	I, II, V, VI, VII	**Length of hospital stay<br>Postoperative vasopressor use**	**JBI Critical Appraisal Checklist for Cohort Studies (Q11)**	**Y**	**Y**	**Y**	**Y**	**U**	**Y**	**Y**	**Y**	**Y**	**Y**			
Ronel I[95] (2023)	Cohort Study – correspondence	n - 29<br>PAS not specified	conservative and extirpative<br>IIABO	elective and emergency cesarean delivery with anticipated hysterectomy<br>elective cesarean delivery with uterine preservation	II, V, VI, VII	**Length of hospital stay<br>Timing of extubation**	**Critical Appraisal Checklist of conference abstracts, letters to the editor and correspondence papers (Q6)**	**Y**	**Y**	**Y**	**Y**	**Y**	**Y**							
Rubab UI[96] (2023)	Case Report	n – 1<br>placenta percreta	extirpative	elective cesarean delivery with anticipated hysterectomy	I, II, III, IV, V, VI, VII	**Timing of extubation<br>pICU admission**	**JBI Critical Appraisal Checklist for Case Reports (Q8)**	**Y**	**Y**	**Y**	**Y**	**Y**	**N**	**N/A**	**Y**					
Russo M[97] (2011)	Case Report	n - 2<br>placenta increta - 1<br>placenta percreta - 1	extirpative<br>IIABO<br>preoperative cystoscopy	elective cesarean hysterectomy	II, III, V, VII	**Length of hospital stay<br>Timing of extubation**	**JBI Critical Appraisal Checklist for Case Reports (Q8)**	**Y**	**Y**	**Y**	**Y**	**Y**	**Y**	**N/A**	**Y**					
Sadashivaiah J[98] (2011)	Case Series	n - 13<br>placenta accreta - 4	conservative<br>UAE	elective cesarean delivery with uterine preservation	II, III, V, VII	**Apgar scale<br>pICU admission**	**JBI Critical Appraisal Checklist for Case Series (Q10)**	**Y**	**Y**	**Y**	**U**	**U**	**Y**	**Y**	**Y**	**N/A**	**Y**			
Salim R[99] (2015)	RCT	n - 27<br>placenta accreta and increta - 21 (not specified)<br>placenta percreta - 1	conservative and extirpative<br>IIABO<br>preoperative cystoscopy	elective cesarean delivery with anticipated hysterectomy	II, III, V, VII	**Length of hospital stay<br>Maternal hospital readmission rate<br>Postoperative opioid use<br>Apgar scale**	**JBI Critical Appraisal Checklist for Randomized Controlled Trials (Q13)**	**Y**	**Y**	**Y**	**N**	**N**	**N**	**Y**	**Y**	**Y**	**Y**	**Y**		
Schiraldi R[100] (2012)	Case Report	n - 1<br>placenta percreta	not specified<br>IIABO	elective not specified	II, III, IV, V, VI, VII	**Length of hospital stay<br>Mechanical ventilation/sedation time<br>Timing of extubation<br>Apgar scale**	**JBI Critical Appraisal Checklist for Case Reports (Q8)**	**Y**	**Y**	**Y**	**U**	**Y**	**Y**	**N/A**	**Y**					

Serrano C[101] (2023)	Case Report	n – 1<br>not specified	extirpative	emergency caesarean hysterectomy	I, II, III, VII	**Length of hospital stay<br>Apgar scale**	**JBI Critical Appraisal Checklist for Case Reports (Q8)**	**Y**	**N**	**Y**	**N**	**Y**	**Y**	**N/A**	**Y**					
Setyawan N[102] (2021)	Case Series	n - 5<br>PAS not specified	extirpative	elective caesarean delivery with anticipated hysterectomy	II, IV, V, VI, VII	**Length of hospital stay<br>Mechanical ventilation/sedation time<br>Timing of extubation<br>Apgar scale**	**JBI Critical Appraisal Checklist for Case Series (Q10)**	**Y**	**U**	**U**	**U**	**U**	**Y**	**Y**	**N**	**N/A**	**Y**			
Seyhan TO[103] (2014)	Case Report	n - 1<br>placenta accreta	extirpative	elective caesarean delivery with anticipated hysterectomy	II, III, IV, V, VII	**Length of hospital stay<br>Postoperative opioid use<br>Apgar scale**	**JBI Critical Appraisal Checklist for Case Reports (Q8)**	**Y**	**Y**	**Y**	**N**	**Y**	**Y**	**N/A**	**Y**					
Shamshirsaz AA[104] (2015)	Cohort Study	n - 90<br>PAS not specified	extirpative<br>preoperative cystoscopy	elective caesarean hysterectomy	II, IV, V, VI, VII	**Length of hospital stay<br>Maternal hospital readmission rate**	**JBI Critical Appraisal Checklist for Cohort Studies (Q11)**	**Y**	**Y**	**Y**	**Y**	**Y**	**Y**	**Y**	**Y**	**Y**	**N/A**	**Y**		
Shantikaratri ET[105] (2023)	Case Report – conference abstract	n - 4<br>PAS not specified	extirpative	elective caesarean hysterectomy	II, III, IV, V, VI	**Postoperative vasopressor use**	**Critical Appraisal Checklist of conference abstracts, letters to the editor and correspondence papers (Q6)**	**Y**	**Y**	**Y**	**N/A**	**Y**	**Y**							
Silva M[106] (2011)	Case Report	n - 1	not specified	emergency not specified	I, II, III, IV, V, VI, VII	**Length of hospital stay**	**JBI Critical Appraisal Checklist for Case Reports (Q8)**	**Y**	**Y**	**Y**	**N**	**Y**	**N**	**N**	**Y**					
Sindos M[107] (2022)	Case Series	n - 69<br>placenta accreta - 7<br>placenta increta - 31<br>placenta percreta - 19	extirpative	elective and emergency caesarean hysterectomy	II, IV, V, VI, VII	**Length of hospital stay<br>Postoperative opioid use<br>Timing of extubation<br>Postoperative vasopressor use<br>Apgar scale**	**JBI Critical Appraisal Checklist for Case Series (Q10)**	**Y**	**Y**	**Y**	**Y**	**Y**	**Y**	**Y**	**Y**	**Y**	**Y**			
Steel C[108] (2010)	Case Report	n - 1<br>placenta accreta - 1	extirpative	emergency caesarean hysterectomy	II, IV, V, VI, VII	**Mechanical ventilation/sedation time<br>Timing of extubation<br>Apgar scale**	**JBI Critical Appraisal Checklist for Case Reports (Q8)**	**Y**	**Y**	**Y**	**Y**	**Y**	**N**	**Y**	**Y**					
Styron AG[109] (2008)	Case Report	n - 1<br>placenta percreta - 1	conservative with delayed hysterectomy<br>IIABO, UE<br>preoperative cystoscopy	elective staged delivery with delayed hysterectomy	I, II, III, IV, V, VI, VII	**Timing of extubation<br>Apgar scale<br>pICU admission**	**JBI Critical Appraisal Checklist for Case Reports (Q8)**	**Y**	**Y**	**Y**	**Y**	**Y**	**Y**	**Y**	**Y**					
Sud S[110] (2020)	Case Report - letter to the editor	n - 1<br>placenta accreta - 1	extirpative<br>IIABO	elective caesarean hysterectomy	I, II, III, V	**Mechanical ventilation/sedation time<br>Timing of extubation**	**Critical Appraisal Checklist of conference abstracts, letters to the editor and correspondence papers (Q6)**	**Y**	**Y**	**N**	**N/A**	**Y**	**N/A**							
Sultan P[111] (2012)	Case Report	n - 1<br>placenta accreta - 1	extirpative	elective caesarean delivery with anticipated hysterectomy	II, IV, V, VI, VII	**Length of hospital stay<br>Postoperative opioid use<br>Apgar scale**	**JBI Critical Appraisal Checklist for Case Reports (Q8)**	**Y**	**Y**	**Y**	**N/A**	**Y**	**Y**	**Y**	**Y**					
Supraptomo RT[112] (2023)	Case Report	n – 1<br>placenta accreta - 1	conservative	elective caesarean delivery with uterine preservation	II, V, VI, VII	**Apgar scale**	**JBI Critical Appraisal Checklist for Case Reports (Q8)**	**Y**	**Y**	**Y**	**Y**	**Y**	**Y**	**N/A**	**Y**					

Supraptomo RT[113] (2021)	Case Report	n - 1<br>placenta percreta	extirpative<br>IAABO	elective cesarean hysterectomy	I, II, III, IV, V, VI, VII	**Length of hospital stay**<br>**Mechanical ventilation/sedation time**<br>**Postoperative opioid use**<br>**Timing of extubation**	**JBI Critical Appraisal Checklist for Case Reports (Q8)**	**Y**	**Y**	**Y**	**Y**	**Y**	**Y**	**N/A**	**Y**					
Takekawa D[114] (2017)	Case Report	n - 1<br>PAS not specified	conservative<br>IIAE	emergency cesarean delivery with uterine preservation	II, III, IV, V, VI, VII	**Length of hospital stay**	**JBI Critical Appraisal Checklist for Case Reports (Q8)**	**Y**	**Y**	**Y**	**N/A**	**Y**	**Y**	**Y**	**Y**					
Taylor NJ[115] (2017)	Case Series	n - 40<br>placenta accreta - 31<br>placenta increta - 2<br>placenta percreta - 7	conservative and extirpative<br>IIABO<br>preoperative cystoscopy	elective and emergency cesarean delivery with anticipated hysterectomy	I, II, III, V, VI, VII	**Length of hospital stay**<br>**Postoperative opioid use**<br>**Timing of extubation**<br>**Postoperative vasopressor use**<br>**Apgar scale**<br>**pICU admission**	**JBI Critical Appraisal Checklist for Case Series (Q10)**	**Y**	**Y**	**Y**	**U**	**U**	**Y**	**Y**	**Y**	**N/A**	**Y**			
Thomas MA[116] (2019)	Case Series - conference abstract	n - 32<br>placenta accreta - 92.9%<br>placenta increta – 3.6%<br>placenta percreta – 3.6%	conservative and extirpative	elective cesarean delivery with anticipated hysterectomy	II, III, IV, V, VI, VII	**Length of hospital stay**	**Critical Appraisal Checklist of conference abstracts, letters to the editor and correspondence papers (Q6)**	**Y**	**Y**	**Y**	**Y**	**Y**	**N/A**							
Thon S[117] (2011)	Case Series	n - 13<br>placenta accreta - 3<br>placenta increta - 1<br>placenta percreta - 2	extirpative<br>IIABO	elective cesarean delivery with anticipated hysterectomy	I, III, V, VI		**JBI Critical Appraisal Checklist for Case Series (Q10)**	**Y**	**Y**	**Y**	**Y**	**Y**	**Y**	**Y**	**Y**	**N/A**	**Y**			
Traversa M[118] (2024)	Case Report - conference abstract	n - 1<br>placenta accreta - 1	extirpative<br>FABO	elective cesarean hysterectomy	I, II, III, VI, VII	**Timing of extubation**<br>**Time to first mobilization**<br>**Maternal satisfaction with analgesia**	**Critical Appraisal Checklist of conference abstracts, letters to the editor and correspondence papers (Q6)**	**Y**	**Y**	**Y**	**Y**	**N/A**	**Y**							
Urfalioglu A[119] (2020)	Cohort Study	n - 95<br>PAS not specified	not specified	elective and emergency cesarean delivery with anticipated hysterectomy	II, III, IV, V, VI, VII	**Mechanical ventilation/sedation time**<br>**Timing of extubation**	**JBI Critical Appraisal Checklist for Cohort Studies (Q11)**	**Y**	**U**	**U**	**N/A**	**N/A**	**Y**	**Y**	**Y**	**N/A**	**N/A**	**Y**		
Usman N[120] (2014)	Case Report - correspondence	n - 1<br>placenta percreta	extirpative<br>REBOA	elective cesarean hysterectomy	II, III, IV, V, VII	**Length of hospital stay**<br>**Mechanical ventilation/sedation time**<br>**Timing of extubation**	**Critical Appraisal Checklist of conference abstracts, letters to the editor and correspondence papers (Q6)**	**Y**	**Y**	**Y**	**N/A**	**Y**	**N/A**							
Valentine S[121] (2019)	Case Report	n - 1<br>placenta percreta	extirpative<br>UAE	elective cesarean delivery with anticipated hysterectomy	II, III, IV, V, VI, VII	**Length of hospital stay**<br>**Postoperative opioid use**<br>**Timing of extubation**<br>**Apgar scale**	**JBI Critical Appraisal Checklist for Case Reports (Q8)**	**Y**	**Y**	**Y**	**Y**	**Y**	**Y**	**N/A**	**Y**					
VanMatre M[122] (2010)	Case Report	n - 1<br>placenta percreta	extirpative	elective cesarean delivery with anticipated hysterectomy	II, III, IV, V, VI	**Timing of extubation**	**JBI Critical Appraisal Checklist for Case Reports (Q8)**	**Y**	**Y**	**Y**	**U**	**Y**	**N**	**Y**	**U**					

Wei X[123] (2016)	Case Series	n - 45<br>placenta accreta - 22<br>placenta increta - 20<br>placenta percreta - 3	conservative and extirpative<br>IAABO	elective cesarean delivery with anticipated hysterectomy	II, III, V, VII	**Length of hospital stay<br>Postoperative opioid use<br>Apgar scale**	**JBI Critical Appraisal Checklist for Case Series (Q10)**	**Y**	**Y**	**Y**	**Y**	**Y**	**Y**	**Y**	**Y**	**N/A**	**Y**			
Weiniger CF[124] (2005)	Cohort Study	n - 28<br>placenta accreta and increta - 26<br>placenta percreta - 2	conservative and extirpative	elective and emergency cesarean delivery with anticipated hysterectomy	I, II, III, IV, V, VI, VII	**Length of hospital stay<br>Mechanical ventilation/sedation time<br>Postoperative opioid use<br>Timing of extubation**	**JBI Critical Appraisal Checklist for Cohort Studies (Q11)**	**Y**	**Y**	**Y**	**N/A**	**N/A**	**Y**	**Y**	**Y**	**N/A**	**N/A**	**Y**		
Wijaya T[125] (2021)	Case Report	n – 1<br>PAS not specified	conservative	elective cesarean delivery with uterine preservation	II, III, IV, V, VI, VII	**Length of hospital stay<br>Mechanical ventilation/sedation time<br>Timing of extubation<br>Apgar scale**	**JBI Critical Appraisal Checklist for Case Reports (Q8)**	**Y**	**Y**	**Y**	**Y**	**U**	**U**	**N/A**	**Y**					
Yamada T[126] (2019)	Case Series	n - 3<br>placenta percreta - 3	extirpative<br>UAE<br>preoperative cystoscopy	elective cesarean hysterectomy	II, IV, V		**JBI Critical Appraisal Checklist for Case Series (Q10)**	**Y**	**Y**	**N/A**	**Y**	**Y**	**N**	**N/A**	**Y**					
Young H[127] (2022)	Cohort Study	n – 30<br>placenta accreta - 14<br>placenta increta - 4<br>placenta percreta - 11	extirpative	elective and emergency cesarean hysterectomy	I, II, V, VI, VII	**Length of hospital stay**	**JBI Critical Appraisal Checklist for Cohort Studies (Q11)**	**Y**	**U**	**U**	**N/A**	**N/A**	**N/A**	**Y**	**Y**	**Y**	**N/A**	**Y**		
Zabida A[128] (2023)	Cohort Study	n - 221<br>placenta accreta - 64<br>placenta increta - 28<br>placenta percreta - 93	conservative and extirpative<br>UABO	elective and emergency cesarean delivery with anticipated hysterectomy	I, II, III, IV, V, VI		**JBI Critical Appraisal Checklist for Cohort Studies (Q11)**	**Y**	**Y**	**Y**	**N/A**	**N/A**	**Y**	**Y**	**Y**	**N/A**	**N/A**	**Y**		
Zhou F[129] (2024)	Cohort Study	n – 375<br>placenta accreta - 102<br>placenta percreta - 273	conservative and extirpative<br>UAE	elective and emergency cesarean delivery with anticipated hysterectomy	I, II, IV, V, VII	**Length of hospital stay<br>Apgar scale<br>pICU admission**	**JBI Critical Appraisal Checklist for Cohort Studies (Q11)**	**Y**	**Y**	**Y**	**Y**	**Y**	**Y**	**Y**	**Y**	**N/A**	**N/A**	**Y**		
Zhu H[130] (2020)	Quasiexperimental Study	n - 48<br>placenta accreta - 20<br>placenta percreta - 10	conservative and extirpative<br>IAABO, UAE	elective cesarean delivery with anticipated hysterectomy	II, III, V, VII	**Apgar scale**	**JBI Critical Appraisal Checklist for Quasiexperimental Studies (Q9)**	**Y**	**Y**	**Y**	**Y**	**N/A**	**N/A**	**Y**	**Y**	**Y**				

Table S2. Identified members of the MDT in the included studies. MDT, multidisciplinary team.

MDT member	No studies
Anesthesiologist	41
Obstetrician	31
Radiologist	11
Interventional Radiologist	18
nursing professionals	7
Neonatologist/pediatrician	29
Intensivist	12
Gynecologist	9
hematologist	3
Gynecologic Oncologist	14
Midwife	3
General Surgeon	5
Blood bank personnel	11
Maternal-fetal medicine	11
Outpatient	1
Inpatient	1
Vascular Surgery	7
Cardio-thoracic surgeon	1
Urologist	18
Laboratory Staff	1
Anesthetic Nurse/paranesthesia	3
Trauma surgeon	4
Pathologist	3
Transfusiologist	4
Perfusionist	2
Psychiatrist	1
Cardiologist	1

Table S3. Comparison of identified management protocols.

		Gatta	Lopez-Erazo	Shamshirsaz	Nguyen-Lu
**preoperative**	**anesthetic evaluation**	Outpatient consultation with obstetric anesthesiologist before scheduled admission, anesthesia consent after admission in the 34th–35th week	Formal evaluation by the anesthesiologist with recommendation of neuraxial anesthesia and evaluation of situations where GA may be preferable	Outpatient consultation with anesthesiologist before scheduled admission	Consultation with anesthesiologist as a part of multidisciplinary assessment
	**other data and recommendations**		Checklist for PAS management, administration of h2 receptor antagonist and prokinetic agent before surgery		Administration of h2 receptor antagonist, prokinetic agent and gabapentin before surgery
**intraoperative**	**mode of anesthesia**	Double catheter technique: thoracic epidural and lumbar CSE	CSE (Th 8–10 epidural and spinal anesthesia)	CSE for cesarean delivery with conversion to GA if hysterectomy is needed	Epidural catheter L2–3 or 3–4 with ultrasound guidance
	**other details**	Anesthesiologist ensuring availability of blood products and TXA	CVC if needed, depending on the hemodynamic status, two units of compatible red blood cells in the operating room	Large-bore venous lines, arterial line and CVC before GA	Large-bore venous lines before IR and arterial line before cesarean delivery
**postoperative**	**postoperative admission**	Postpartum floor if clinically stable		ICU	Labour & Delivery/ICU
	**other details**	Thoracic epidural for postoperative pain control			Epidural for postoperative pain control, epidural catheter removal after 24 hours

Table S4. Detailed data on the IR procedures used for PAS delivery. IR, interventional radiology; PAS, placenta accreta spectrum; OR, operating room: NA, neuraxial anesthesia; GA, general anesthesia.

	RCT	Quasiexperimental studies	Cohort studies	Case series	Case reports
Hybrid room utilization for IR PAS surgery		Zhu H	Gatta LA, Ronel I	Chao WT, Dolling S, Riveros-Perez E, Wei X, Yamada T	Konishi Y, Mauritz AA, Mok M, Valentine S
IR PAS surgery in OR		Clapham E, Lopez-Erazo LJ, Nieto AJ	Panigrahi AK	Mok M, Taylor NJ	Bell-Thomas SM, Ji SM, Morland D, Oshugi E, Sud S, Supraptomo RT (2021), Supraptomo RT (2023) Usman
IR with subsequent transfer to OR	Salim R	Feng S, Lopez-Erazo LJ	Nguyen-Lu N	Hania A, Lan JY, Riveros-Perez E, Sadashivaiah J, Thon S	Alford B, Chavez R, Dhansura T, Kessack LK, Kuczkowski KM, Ma Y (2019), Mok M, Morland D, Omowanile YA, Pandey M, Parva M, Paull JD, Russo M, Schiraldi R, Styron AG
IR procedures and GA only anesthesia	Salim R	Feng S, Lopez-Erazo LJ, Nieto AJ	Nguyen-Lu N, Liu C, Zabida A,	Chao WT, Hegde H, Lan JY, Kume K, Mok M, Patil Y Riveros-Perez E, Thon S, Wei X	Dhansura T, Hajmurad OS, Konishi Y, Ma Y (2019), Pandey M, Pini R, Russo M, Styron AG, Takekawa D
Timing of GA after IR	Salim R	Feng S	Nguyen-Lu N,	Akinaga C, Chao WT, Hania A, Lan JY, Lilker SJ, Patil Y, Taylor NJ, Thon S	Chavez R, Dhansura T, Hajmurad OS, Kessack LK, Ma Y (2019), Mok M , Omowanile YA, Pandey M, Paull JD, Pini R, Russo M, Styron AG, Sud S, Supraptomo RT, Wei X
Timing of GA before IR		Lopez-Erazo LJ		Akinaga C	Mok M
IR PAS procedures and NA anesthesia		Lopez-Erazo LJ, Nieto AJ, Nieto-Calvache AJ (2022), Zhu H	Herbert KA, Markley JC, Neef V, Nguyen-Lu N, Panigrahi AK, Ronel I, Zabida A,	Akinaga C, Dolling S, Hania A, Lan JY, Lilker SJ, Malik Z, Riveros-Perez E, Sadashivaiah J, Taylor NJ, Thon S, Wei X, Yamada T	Alford B, Bell-Thomas SM, Chavez R, Ji SM, Kessack LK, Konishi Y, Kuczkowski KM, Mauritz AA, Morland D, Otero CR, Parva M, Russo M, Schiraldi R, Supraptomo RT (2023), Traversa M, Usman N, Valentine S
Timing of NA before IR			Neef V, Nguyen-Lu N	Hania A, Lilker SJ, Wei X, Yamada T	Alford B, Chavez R , Kessack LK, Mauritz AA, Otero CR, Parva M, Russo M, Sadashivaiah J, Schiraldi R, Supraptomo RT (2023) Traversa M
Timing of NA after IR				Lan JY	Konishi Y, Kuczkowski KM, Morland D, Valentine S
IR PAS procedures completed in NA only anesthesia		Nieto AJ, Zhu H	Herbert KA, Neef V, Nguyen-Lu N, Ronel I	Lan JY, Lilker SJ, Riveros-Perez E, Sadashivaiah J, Taylor NJ, Wei X	Kessack LK, Kuczkowski KM, Morland D, Russo M, Traversa M
IR PAS surgery completed in NA + GA anesthesia		Lopez-Erazo LJ, Nieto AJ	Herbert KA, Nguyen-Lu N, Ronel I	Akinaga C, Dolling S, Lan JY, Lilker SJ, Malik Z, Riveros-Perez E, , Sadashivaiah J , Thon S, Wei X, Yamada T	Bell-Thomas SM, Chavez R, Ji SM, Mauritz AA, Otero CR, Parva M, Schiraldi R, Usman N, Valentine S

Table S5. Complementary data on the main issues of interest. CD, cesarean delivery.

	RCT	Quasiexperimental studies	Cohort studies	Case series	Case reports
**General data**					
Presence of risk factors for PAS development	Salim R	Feng S, Nieto AJ, Zhu H	Angstmann T, Binici O, Buyukkurt S Gatta LA, Herbert KA, Kocaoglu N, Liu C, Mansouri M, Markley JC, Neef V, Nguyen-Lu N, Oygen Ö, Panigrahi AK, Panjeton GD, Ronel I, Shamshirsaz AA, Weiniger CF, Young H, Zabida A, Zhou F	Ahmad M, Alvarado-Ramos S, Biji KP, Butwick AJ, Chao WT, Hania A, Hegde H, Ioscovich A, Karacaer F, Korolev A, Kume K , Lilker SJ, Mok M , Munoz LA, Patil Y, Riveros-Perez E, Sadashivaiah J, Setyawan N, Shantikaratri E, Sindos M, Taylor NJ, Thomas MA, Thon S, Wei X, Yamada T	Alford B, Batra J, Anthony Cometa M, Bamidele B, Bell-Thomas SM, Bergakker SA , Bhavani-Shankar K, Busani S, Chavez R, Churchill S, Dhansura T, Dogra S, Estella NM, Faris AS, Feldman JB, Frasca D, Glynn JC , Günaydın B , Hunter T, Hajmurad OS, Humphrey J, Hunter T , Ismail S, Ji SM, Jiang X, Kessack LK, Khokhar RS, Konishi Y , Kuczkowski KM, Laranjo M, Li P , Ma Y (2019), Ma Y (2020), Mauritz AA, Morland D, Nagy CJ , Nanji JA, Omowanile YA , Orr T , Oshugi E, Pandey M, Pareek S, Parva M, Paull JD, Pini R, Rubab UI, Russo M, Schiraldi R, Seyhan TO, Silva M, Steel C, Styron AG, Sud S, Sultan P, Supraptomo RT, Supraptomo RT (2023), Traversa M, Usman N, Valentine S, Wijaya T
Previous CD	Salim R	Feng S, Nieto AJ, Zhu H	Angstmann T, Binici O, Buyukkurt S, Gatta LA, Herbert KA, Kocaoglu N, Liu C, Mansouri M, Markley JC, Neef V, Nguyen-Lu N, Oygen Ö, Panigrahi AK, Panjeton GD, Ronel I, Shamshirsaz AA, Weiniger CF, Young H, Zabida A, Zhou F	Ahmad M, Alvarado-Ramos S, Biji KP, Butwick AJ, Chao WT, Hania A, Hegde H, Ioscovich A, Karacaer F, Korolev A, Kume K , Mok M , Munoz LA, Patil Y, Riveros-Perez E, Sadashivaiah J, Setyawan N, Shantikaratri E, Sindos M, Taylor NJ, Thomas MA, Thon S, Wei X, Yamada T	Alford B, Bamidele B, Batra J, Bell-Thomas SM, Bergakker SA , Bhavani-Shankar K, Busani S, Chavez R, Churchill S, Dhansura T, Dogra S, Estella NM, Faris AS, Feldman JB, Frasca D, Glynn JC , Günaydın B, Hajmurad OS, Humphrey J, Hunter T , Ismail S, Ji SM, Jiang X, Kessack LK, Khokhar RS, Konishi Y, Kuczkowski KM, Laranjo M, Li P , Ma Y (2019), Ma Y (2020), Mauritz AA, Morland D, Nagy CJ , Nanji JA, Omowanile YA , Orr T , Oshugi E, Pandey M, Pareek S, Parva M, Paull JD, Rubab UI, Russo M, Schiraldi R, Seyhan TO, Silva M, Steel C, Styron AG, Sud S, Sultan P, Supraptomo RT, Supraptomo RT (2023), Traversa M, Usman N, Valentine S, Wijaya T
Presence of placenta previa	Salim R	Zhu H	Angstmann T, Binici O, Buyukkurt S, Gatta LA, Herbert KA, Kocaoglu N, Liu C, Markley JC, Neef V, Nguyen-Lu N, Oygen Ö, Panigrahi AK, Panjeton GD, Ronel I, Weiniger CF, Young H, Zabida A, Zhou F	Ahmad M, Akinaga C, Butwick AJ, Hegde H, Ioscovich A, Karacaer F, Lilker SJ, Mok M , Munoz LA, Patil Y, Riveros-Perez E, Sindos M, Taylor NJ, Yamada T	Batra J, Bell-Thomas SM, Busani S, Estella NM, Faris AS, Glynn JC , Günaydın B , Hajmurad OS, Humphrey J, Hunter T, Jiang X, Kessack LK, Kuczkowski KM, Mauritz AA, Nanji JA, Pandey M, Pareek S, Parva M, Paull JD, Pini R, Rubab UI, Russo M, Silva M, Steel C, Sultan P, Supraptomo RT, Traversa M, Valentine S, Wijaya T
Previous uterine instrumentation	Salim R	Nieto AJ	Liu C, Nguyen-Lu N, Young H	Chao WT, Karacaer F, Korolev A, Lilker SJ,	Frasca D, Humphrey J, Ismail S, Ji SM, Kuczkowski KM, Li P, Ma Y (2020), Russo M, Schiraldi R, Steel C, Supraptomo RT (2023)

(unspecified or aside from CD)				Munoz LA, Setyawan N, Taylor NJ, Yamada T	
**Structure of anesthetic service**					
Anesthesiologist as an MDT member		Lekic Z, Lopez-Erazo LJ, Loreto M, Nieto-Calvache AJ (2022),	Angstmann T, Gatta LA, Mansouri M, Panigrahi AK, Rana S, Shamshirsaz AA, Weiniger CF	Alvarado-Ramos S, Biji KP, Chao WT, Lilker SJ, Mok M, Munoz LA, Taylor NJ	Alford B, Batra J, Busani S, Dhansura T, Faris AS, Hunter T, Ismail S, Ji SM, Jiang X, Khokhar RS, Li P, Mauritz AA, Otero CR, Pareek S, Parva M, Rubab UI, Russo M, Serrano C, Styron AG, Sud S, Traversa M, Valentine S
**Periprocedural monitoring**					
Monitoring of the depth of anesthesia			Nguyen-Lu N		Laranjo M, Orr T, Otero CR
Monitoring of neuromuscular blockade					Ismail S
The use of an arterial line	Abdullah S, Plakhotina EN, Salim R	Clapham E, Feng S, Lekic Z , Lopez-Erazo LJ, Zhu H, , Loreto M	Angstmann T, Gatta LA, Mansouri M, Markley JC, Neef V, Nguyen-Lu N, Panigrahi AK, Panjeton GD, , Shamshirsaz AA, Urfalioglu A, Weiniger CF, Zabida A	Ahmad M, Akinaga C, Alvarado-Ramos S, Biji KP, Butwick AJ, Chao WT, Dayican HY, Dolling S, Elagamy A, Hania A, Hegde H, Ioscovich A, Lan JY, Karacaer F, Malik Z, Mok M , Munoz LA, Sadashivaiah J, Shantikaratri E, Sindos M, Taylor NJ, Thomas MA, Thon S, Wei X, Yamada T, Elagamy A,	Anthony Cometa M, Bamidele B, Bell-Thomas SM, Bhavani-Shankar K , Biele C , Busani S, Chavez R, Churchill S, Dhansura T, Dogra S, Estella NM, Faris AS, Feldman JB, Frasca D, Hajmurad OS, Humphrey J, Hunter T , Ismail S, Jiang X, Ji SM, Kamani AA , Khokhar RS, Konishi Y , Kuczkowski KM, Li P , Ma Y (2019), Mauritz AA, Morland D, Nagy CJ , Nanji JA, Omowanile YA , Orr T, Oshugi E, Otero CR, Pandey M, Parva M, Paull JD, Pini R, Rubab UI, Russo M, Schiraldi R, Serrano C, Seyhan TO, Silva M, Steel C, Styron AG, Sud S, Sultan P, Supraptomo RT, Takekawa D, Traversa M, Usman N, Valentine S, VanMatre M
Monitoring of central venous pressure		Zhu H	Rana S, Urfalioglu A	Biji KP	Dogra S, Estella NM, Faris AS, Kamani AA, , Oshugi E
The use of minimally invasive and non-invasive hemodynamic monitoring	Abdullah S	Clapham E, Loreto M		Alvarado-Ramos S	Morland D, Nagy CJ, Oshugi E, Traversa M
The use of trans esophageal echocardiography or doppler					Batra J, Nanji JA, Oshugi E, Schiraldi R
**Hemodynamic management and fluid therapy**					

Type or volume of crystalloids infused	Plakhotina EN	Clapham E, Loreto M, Nieto AJ	Binici O, Mansouri M, Nguyen-Lu N, Oygen Ö, Shamshirsaz AA, Urfalioglu A, Weiniger CF, Zabida A	Alvarado-Ramos S, Biji KP, Butwick AJ, Chao WT, Hegde H, Ioscovich A, Karacaer F, Korolev A, Sindos M, Wei X	Bamidele B, Bergakker SA , Bhavani-Shankar K, Busani S, Dogra S, Estella NM, Feldman JB, Frasca D, Hajmurad OS, Humphrey J, Hunter T, Kamani AA, Kessack LK, Khokhar RS, Konishi Y, Laranjo M, Ma Y (2020), Mauritz AA, Nagy CJ, Oshugi E, Rubab UI, Russo M, Schiraldi R, Seyhan TO, Silva M, Steel C, Styron AG, Sud S, Sultan P, Supraptomo RT, Takekawa D, Usman N, Valentine S, Wijaya T
Type or volume of colloids infused	Abdullah S		Binici O, Nguyen-Lu N, Shamshirsaz AA, Urfalioglu A, Weiniger CF	Butwick AJ, Dayican HY, Hegde H, Karacaer F, Korolev A	Bamidele B, Bell-Thomas SM, Bhavani ,Bhavani-Shankar K, Humphrey J, Jiang X, Laranjo M, Ma Y (2020), Nagy CJ, Parva M, Rubab UI, Schiraldi R, Silva M, Steel C, Sultan P, Supraptomo RT, Takekawa D, Usman N, Valentine S, VanMatre M, Wijaya T
Two large-bore peripheral cannulas scheme	Salim R	Loreto M	Angstmann T, Rana S, Panjeton GD, Zabida A	Biji KP, Elagamy A, Shantikaratri E, Thon S, Lan JY, Yamada T	Bell-Thomas SM, Bergakker SA ,Biele C, Dhansura T, Dogra S, Hunter T, Khokhar RS, Laranjo M, Nagy CJ, Oshugi E, Rubab UI, Russo M, Supraptomo RT, Usman N, VanMatre M, Bamidele B, Günaydın B, Humphrey J, Ji SM , Omowanile YA, Pandey M, Pareek S, Paull JD, Pini R, Seyhan TO, Styron AG, Valentine S
Central venous cannulation	Abdullah S, Plakhotina EN, Salim R	Nieto AJ, Zhu H	Angstmann T, Kocaoglu N, Neef V, Rana S, Shamshirsaz AA, Urfalioglu A	Ahmad M, Akinaga C, Alvarado-Ramos S, Biji KP, Butwick AJ, Chao WT, Dayican HY, Hegde H, Ioscovich A, Korolev A, Lan JY, Mok M , Munoz LA, Nguyen-Lu N, Riveros-Perez E, Sadashivaiah J, Setyawan N, Sindos M, Taylor NJ, Thomas MA, Wei X, Yamada T	Chavez R, Dogra S, Estella NM, Faris AS, Frasca D, Hajmurad OS, Hunter T, Ismail S, Kamani AA, , Khokhar RS, , Konishi Y , Kuczkowski KM, Li P, Nagy CJ , Nanji JA, Oshugi E, Pareek S, Parva M, Rubab UI, Russo M, Schiraldi R, Silva M, Supraptomo RT, Usman N, VanMatre M
The use of phenylephrine	Plakhotina EN		Nguyen-Lu N, Panjeton GD, Markley JC		Bamidele B, Bergakker SA, Bhavani-Shankar K, Frasca D, Humphrey J, Hunter T, Ismail S, Kessack LK, Konishi Y, Laranjo M, Mauritz AA, Morland D, Nagy CJ, Styron AG, VanMatre M
The use of ephedrine		Loreto M	Nguyen-Lu N, Panjeton GD, Markley JC	Alvarado-Ramos S, Butwick AJ	Anthony Cometa M, Bamidele B, Bergakker SA, Bhavani-Shankar K, Humphrey J, Laranjo M, Ma Y (2020), Seyhan TO, Styron AG, VanMatre M
The use of norepinephrine		Loreto M		Biji KP, Dayican HY, Karacaer F, Korolev A, Kume K, Munoz LA, Taylor NJ	Batra J, Dogra S, Feldman JB, Hunter T, Laranjo M, Pini R, Rubab UI, Schiraldi R

The use of adrenaline				Butwick AJ	Rubab UI, Seyhan TO
The use of dopamine				Karacaer F, Kume K	Hunter T
The use of vasopressin					Batra J
**Blood product management and hemostasis**					
Target values or thresholds for blood product administration provided		Feng S	Zabida A, Neef V	Alvarado-Ramos S, Biji KP, Ioscovich A, Karacaer F, Munoz LA, Sadashivaiah J	Bergakker SA, Busani S
The use of fixed blood product ratio used			Panigrahi AK, Shamshirsaz AA	Ahmad M	Ismail S, Pareek S, Usman N
The use of thromboelastography				Alvarado-Ramos S, Dolling S	Biele C, Churchill S, Feldman JB, Nanji JA
The use of cryoprecipitate	Salim R	Nieto AJ, Nieto-Calvache AJ (2022)	Nguyen-Lu N, Panigrahi AK, Ronel I, Weiniger CF	Alvarado-Ramos S, Biji KP, Dolling S, Hegde H, Karacaer F, Malik Z, Taylor NJ, Thomas MA,	Alford B, Bell-Thomas SM, Faris AS, Glynn JC, Ismail S, , Jiang X, Kamani AA, Kessack LK, Nanji JA, Sultan P, Valentine S
The use of tranexamic acid		Nieto AJ, Nieto-Calvache AJ (2022)	Binici O, Neef V, Nguyen-Lu N, Oygen Ö, Rana S, Young H, Zabida A	Dayican HY, Dolling S, Karacaer F, Hegde H, Korolev A, Sindos M, Taylor NJ	Biele C, Busani S, Dogra S, Faris AS, Günaydın B , Hajmurad OS, Laranjo M, Mauritz AA, Pandey M, Pini R, Rubab UI, Schiraldi R, Supraptomo RT (2023), Usman N
The use of recombined factor VII				Thon S, Sindos M	Faris AS, Frasca D, Glynn JC, Kessack LK, Schiraldi R, Usman N
The use of fibrinogen		Clapham E	Binici O, Neef V, Oygen Ö		Biele C, Churchill S, Jiang X, Laranjo M, Pini R, Schiraldi R
The use of prothrombin complex			Neef V	Sindos M	Biele C, Glynn JC
**Postoperative care**					
ICU admission due to the need for mechanical ventilation			Buyukkurt S, Kocaoglu N , Markley JC , Nguyen-Lu N, Urfalioglu A, Zabida A	Biji KP, Kume K, Lilker SJ, Munoz LA, Riveros-Perez E, Setyawan N, Taylor NJ	Bell-Thomas SM, Faris AS, Feldman JB, Frasca D, Humphrey J, Ji SM, Orr T, Steel C, Sultan P, Takekawa D, Usman N, Wijaya T

1. Abdullah, S.; Abdeltawab, M.; Youssry, M.; Elsonbaty, A.I. The Use of Thoracic Fluid Content As A Guide For 6% HES Infusion during Hypervolemic Hemodilution Among Placenta Accreta Patients Undergoing Cesarean Section. A Randomized Controlled Trial. *Egyptian Journal of Hospital Medicine* **2022**, *88*, 3834–3838, doi:10.21608/EJHM.2022.252241.
2. Ahmad, M. Strategies for Anesthesia Management of Cesarean Deliveries with Abnormal Placental Placement–Anesthetist's Perspective. *Pakistan Armed Forces Medical Journal* **2021**, *71*, 1778–1781, doi:10.51253/pafmj.v71i5.5381.
3. Akinaga, C.; Taniguchi, M.; Naruse, S.; Asaba, H.; Nakajima, Y. General Anesthesia at Cesarean Section for Placenta Previa. *J. Anesth.* **2024**, *38*, 291–292, doi:10.1007/s00540-024-03313-9.
4. Alford, B.M. Suspected Placenta Accreta. *International Student Journal of Nurse Anesthesia* **2019**, *18*, 31–34.
5. Alvarado-Ramos, S.; Lara-Díaz, V.J.; López-Gutiérrez, M.R.; Torcida-González, M.E.; Campos-Rodríguez, J.F. Minimally Invasive Hemodynamic Assessment during Obstetric Hysterectomy for Invasive Placentation with Epidural Anesthesia. *Anesthesiology Research and Practice* **2020**, *2020*, doi:10.1155/2020/1968354.
6. Angstmann, T.; Gard, G.; Harrington, T.; Ward, E.; Thomson, A.; Giles, W. Surgical Management of Placenta Accreta: A Cohort Series and Suggested Approach. *American Journal of Obstetrics and Gynecology* **2010**, *202*, 38.e1-38.e9, doi:10.1016/j.ajog.2009.08.037.
7. Anthony Cometa, M.; Wasilko, S.M.; Wendling, A.L. Concurrent Intraoperative Uterine Rupture and Placenta Accreta. Do Preoperative Chronic Hypertension, Preterm Premature Rupture of Membranes, Chorioamnionitis, and Placental Abruption Provide Warning to This Rare Occurrence? *Romanian Journal of Anaesthesia and Intensive Care* **2018**, *25*, 83–85, doi:10.21454/rjaic.7518.251.acc.
8. Bamidele, B. Anesthetic Management of Placenta Accreta. *International Student Journal of Nurse Anesthesia* **2018**, *17*, 19–22.
9. Batra, J.; Mourad, M.; Collado, F.K.; Takeda, K.; Greenwald, A.; Ring, L.; Chen, X.; Haythe, J. Case Report of Pregnancy Complicated by Severe Pulmonary Hypertension from Mitral Stenosis and Placenta Accreta Spectrum Disorder: Management of Two Life-Threatening Conditions. *Eur Heart J Case Rep* **2024**, *8*, ytae055, doi:10.1093/ehjcr/ytae055.
10. Bell-Thomas, S.M.; Penketh, R.J.; Lord, R.H.; Davies, N.J.; Collis, R. Emergency Use of a Transfemoral Aortic Occlusion Catheter to Control Massive Haemorrhage at Caesarean Hysterectomy. *BJOG: An International Journal of Obstetrics and Gynaecology* **2003**, *110*, 1120–1122, doi:10.1111/j.1471-0528.2003.01133.x.
11. Bergakker, S.A. Case Report: Management of Elective Cesarean Delivery in the Presence of Placenta Previa and Placenta Accreta. *AANA Journal* **2010**, *78*, 380–384, doi:PMID: 21067085.
12. Bhavani-Shankar, K.; Lynch, E.P.; Datta, S. Airway Changes during Cesarean Hysterectomy. *Canadian Journal of Anesthesia* **2000**, *47*, 338–341, doi:10.1007/BF03020949.
13. Biele, C.; Kaufner, L.; Schwickert, A.; Nonnenmacher, A.; von Weizsäcker, K.; Muallem, M.Z.; Henrich, W.; Braun, T. Conservative Management of Abnormally Invasive Placenta Complicated by Local Hyperfibrinolysis and Beginning Disseminated Intravascular Coagulation. *Archives of Gynecology and Obstetrics* **2021**, *303*, 61–68, doi:10.1007/s00404-020-05721-0.
14. Biji, K.P.; Rashmi, R.; Rekha, K.; Vineetha, P.; Abida, B. Protocol Based Blood Management in Major Obstetric Haemorrhage- A Case Series. *Journal of Clinical and Diagnostic Research* **2021**, *15*, UR01–UR04, doi:10.7860/JCDR/2021/50467.15209.
15. Binici, O.; Buyukfirat, E. Anesthesia for Cesarean Section in Parturients with Abnormal Placentation: A Retrospective Study. *Cureus* **2019**, *11*, doi:10.7759/cureus.5033.
16. Busani, S.; Ghirardini, A.; Petrella, E.; Neri, I.; Casari, F.; Venturelli, D.; De Santis, M.; Montagnani, G.; Facchinetti, F.; Girardis, M. A Challenging Case of Pregnancy with Placenta Accreta and Very Rare Irregular Antibodies versus Cromer Blood Group System: A Case Report. *J Med Case Rep* **2015**, *9*, 112, doi:10.1186/s13256-015-0607-7.
17. Butwick A.J.;Hilton G.;Riley E.T.;Carvalho B.; Non-Invasive Measurement of Hemoglobin during Cesarean Hysterectomy: A Case Series. *International Journal of Obstetric Anesthesia* **2011**, *20*, 240–245, doi:10.1016/j.ijoa.2011.03.009.
18. Buyukkurt, S.; Sucu, M.; Hatipoglu, I.; Ozlu, F.; Unlugenc, H.; Evruke, C.; Demir, C. Placenta Accreta Spectrum Surgery with the Joel Cohen Incision for Abdominal Access: A Single-Center Experience. *Ginekol. Pol.* **2023**, *94*, 532–538, doi:10.5603/GP.a2023.0050.
19. Chao, W.-T.; Ke, H.-H.; Shen, S.-H.; Yeh, C.-C.; Wang, P.-H.; Ho, C.-M.; Horng, H.-C. A Theoretical Analysis of Prophylactic Common Iliac Arterial Occlusion for Potential Massive Bleeding during Cesarean Delivery: Decision-Making Considerations — A 2-Year Retrospective Study. *Taiwanese Journal of Obstetrics and Gynecology* **2022**, *61*, 282–289, doi:10.1016/j.tjog.2022.02.016.
20. Chavez, R. Placental Increta: A Case Study. *International Student Journal of Nurse Anesthesia* **2017**, *16*, 38–41.

21. Churchill, S.; Stacey, M. Placenta Percreta and Uterine Rupture. *Anaesthesia* **2018**, *73*, 49, doi:10.1111/anae.14192.
22. Clapham, E.; Hammon, L.; Newton, R.; Woolnough, M. Impact of Introduction of Enhanced Recovery Protocol for Abnormally Invasive Placentation Cases. *International Journal of Obstetric Anesthesia* **2018**, *35*, S24.
23. Dayican, H.Y.; Akkus, I.B. Anesthetic Approach in Case of Placenta Percreata: Case Series. *Anestezi Derg.* **2023**, *31*, 158–161.
24. Dhansura, T.; Kapadia, D.; Bhorkar, N.; Shaikh, T. Anaesthesiologist's Role in the Multidisciplinary Approach to Placenta Percreta. *Indian Journal of Anaesthesia* **2015**, *59*, 513–515, doi:10.4103/0019-5049.163002.
25. Dogra, S.; Surampadi, K.; Madapu, D.; Pandya, S. Vascular Clamps in Perimortem Caesarean Delivery in Parturients with Placenta Accreta Spectrum: Case Report and Literature Review. *Journal of Obstetric Anaesthesia and Critical Care* **2023**, *13*, 94–98, doi:10.4103/JOACC.JOACC_33_22.
26. Dolling, S.; Bose, L.; Weale, N. Experience of Managing Abnormally Invasive Placenta over an Eight-Year Period in a UK Centre. *International Journal of Obstetric Anesthesia* **2022**, *50*, 23, doi:10.1016/j.ijoa.2022.103329.
27. Elagamy A.;Abdelaziz A.;Ellaithy M.; The Use of Cell Salvage in Women Undergoing Cesarean Hysterectomy for Abnormal Placentation. *International Journal of Obstetric Anesthesia* **2013**, *22*, 289–293, doi:10.1016/j.ijoa.2013.05.007.
28. Estella, N.M.; Berry, D.L.; Baker, B.W.; Wali, A.; Belfort, M.A. Normovolemic Hemodilution before Cesarean Hysterectomy for Placenta Percreta. *Obstetrics and Gynecology* **1997**, *90*, 669–670, doi:10.1016/S0029-7844(97)00394-3.
29. Faris Ali S;Tawfic Qutaiba A;Jeyaraj Leo; Abnormal Placentation: Challenges and Keys for Success. *J Anesth Clin Care* **2014**, *1: 003*, doi:10.24966/ACC-8879/100003.
30. Feldman Joshua Bradley;Kumaraswami Sangeeta; Cesarean Hysterectomy in a Parturient with Morbidly Adherent Placenta Complicated by Postoperative Ischemic Stroke Secondary to Vertebral Artery Dissection: A Case Report. **2019**, *12*, 14.
31. Feng, S.; Liao, Z.; Huang, H. Effect of Prophylactic Placement of Internal Iliac Artery Balloon Catheters on Outcomes of Women with Placenta Accreta: An Impact Study. *Anaesthesia* **2017**, *72*, 853–858, doi:10.1111/anae.13895.
32. Frasca, D. A Cesarean Hysterectomy for Invading Placenta Percreta: Anesthetic Safety Considerations- A Case Report. *AANA Journal* **2012**, *80*, 373–378, doi:PMID: 26050278.
33. Gatta, L.A.; Weber, J.M.; Gilner, J.B.; Lee, P.S.; Grotegut, C.A.; Herbert, K.A.; Bashir, M.; Pieper, C.F.; Ronald, J.; Pabon-Ramos, W.; et al. Transfusion Requirements with Hybrid Management of Placenta Accreta Spectrum Incorporating Targeted Embolization and a Selective Use of Delayed Hysterectomy. *American Journal of Perinatology* **2022**, *29*, 1503–1513, doi:10.1055/s-0042-1754321.
34. Glynn, J.C.; Plaat, F. Prothrombin Complex for Massive Obstetric Haemorrhage 21. *Anaesthesia* **2007**, *62*, 202–203, doi:10.1111/j.1365-2044.2007.04972.x.
35. Günaydın B.;Kurdoğlu M.;Güler İ.;Bashiri M.;Büyüktaşkın F.;Keleşoğlu M.D.;İnan G.; Management of Neuraxial Anaesthesia for Emergent Caesarean Section for Placenta Previa. *Turk J Anaesthesiol Reanim* **2016**, *44*, 43, doi:10.5152/TJAR.2016.52385.
36. Hajmurad Omar S.;Choxi Ankeet A.;Zahid Zahira;Dudaryk Roman; Aortoiliac Thrombosis Following Tranexamic Acid Administration During Urgent Cesarean Hysterectomy: A Case Report. *A A Case Rep* **2017**, *9*, 93.
37. Hania A.;Harnett C.;Morrison J.;Klemmer K.;Costello J.; Placenta Accreta Spectrum: A 2-Year Retrospective Observational Study. *Irish medical journal* **2022**, *115*, 629, doi:PMID: 36300594.
38. Hegde, H.V.; Joshiraj, B.; Paul, M.; Weerasinghe, A.S.; Mulgund, K.G.; Marimuthu, S.R.; Pai, A.G.; Abdullah, R.M. A Retrospective Study in 12-Year Cohort of 216 Parturients with Invasive Placentation in a Tertiary Care Centre in Oman: Clinical Outcomes Indicate Severe Maternal Morbidity. *J Obstet Gynaecol Can* **2024**, *46*, 102341, doi:10.1016/j.jogc.2023.102341.
39. Herbert, K.A.; Gatta, L.A.; Fuller, M.; Grotegut, C.A.; Gilner, J.; Habib, A.S. Anesthetic Management of Placenta Accreta Spectrum at an Academic Center and a Comparison of the Combined Spinal Epidural with the Double Catheter Technique: A Retrospective Study. *Journal of Clinical Anesthesia* **2022**, *77*, N.PAG-N.PAG, doi:10.1016/j.jclinane.2021.110573.
40. Humphrey, J. Primary Cesarean Delivery Results in Emergency Hysterectomy Due to Placenta Accreta: A Case Study. *AANA Journal* **2015**, *83*, 28–34, doi:PMID: 25842631.
41. Hunter, T.; Kleiman, S. Anaesthesia for Caesarean Hysterectomy in a Patient with a Preoperative Diagnosis of Placenta Percreta with Invasion of the Urinary Bladder. *Canadian Journal of Anaesthesia* **1996**, *43*, 246–251, doi:10.1007/bf03011743.

42. Ioscovich, A.; Mirochnitchenko, E.; Halpern, S.; Samueloff, A.; Grisaru-Granovsky, S.; Gozal, Y.; Einav, S. Perioperative Anaesthetic Management of High-Order Repeat Caesarean Section: Audit of Practice in a University-Affiliated Medical Centre. *International Journal of Obstetric Anesthesia* **2009**, *18*, 314–319, doi:10.1016/j.ijoa.2009.01.014.
43. Ismail, S.; Azizullah, A. Management of Parturient with Triplet Pregnancy and Placenta Percreta: Importance of Multi-Disciplinary Approach. *Journal of Obstetric Anaesthesia and Critical Care* **2019**, *9*, doi:10.4103/joacc.JOACC_60_18.
44. Lan Jheng-Yan;Wang Mao-Hsien;Fan Shou-Zen;Chen Li-Kuei; Impact of Anesthetic Methods on Neonatal Outcome in Women Receiving Temporary Balloon Occlusion of the Common Iliac Artery during Cesarean Section for Placenta Accreta. *Taiwanese Journal of Obstetrics and Gynecology* **2011**, *50*, 515–517, doi:10.1016/j.tjog.2011.10.021.
45. Ji, S.M.; Cho, C.; Choi, G.; Song, J.; Kwon, M.A.; Park, J.H.; Kim, S. Successful Management of Uncontrolled Postpartum Hemorrhage Due to Morbidly Adherent Placenta with Resuscitative Endovascular Balloon Occlusion of the Aorta during Emergency Cesarean Section - A Case Report. *Anesth Pain Med (Seoul)* **2020**, *15*, 314–318, doi:10.17085/apm.19051.
46. Jiang, X.; Lin, X.; Han, X.; Ma, Y.; Zhao, F. Successful Resuscitation of a Patient with Pernicious Placenta Previa and Placenta Accreta Who Had Massive Life-Threatening Bleeding during Cesarean Section : A Case Report. *Medicine (United States)* **2019**, *98*, doi:10.1097/MD.0000000000015025.
47. Kamani A.A.S.;Gambling D.R.;Christilaw J.;Flanagan M.L.; Anaesthetic Management of Patients with Placenta Accreta. *Canadian Journal of Anaesthesia* **1987**, *34*, 613–617, doi:10.1007/BF03010522.
48. Karacaer, F.; Biricik, E.; Ilgınel, M.; Tunay, D.; Sucu, M.; Ünlügenç, H. Retrospective Analysis of Eighty-Nine Caesarean Section Cases with Abnormal Placental Invasion. *Turkish Journal of Anaesthesiology and Reanimation* **2019**, *47*, 112–119, doi:10.5152/TJAR.2018.31799.
49. Kessack, L.K.; Hawkins, N. Severe Hypotension Related to Cell Salvaged Blood Transfusion in Obstetrics. *Anaesthesia* **2010**, *65*, 745–748, doi:10.1111/j.1365-2044.2010.06301.x.
50. Khokhar, R.S.; Baaj, J.; Khan, M.U.; Dammas, F.A.; Rashid, N. Placenta Accreta and Anesthesia: A Multidisciplinary Approach. *Saudi Journal of Anaesthesia* **2016**, *10*, 332–334, doi:10.4103/1658-354X.174913.
51. Kocaoglu, N.; Gunusen, I.; Karaman, S.; Ergenoglu, A.M.; Firat, V. Management of Anesthesia for Cesarean Section in Parturients with Placenta Previa with/without Placenta Accreta: A Retrospective Study. *Ginekologia polska* **2012**, *83*, 99–103, doi:PMID: 22568353.
52. Konishi, Y.; Yamamoto, S.; Sugiki, K.; Sakamoto, H.; Sawamura, S. A Novel and Multidisciplinary Strategy for Cesarean Delivery with Placenta Percreta: Intraoperative Embolization in a Hybrid Suite. *A and A Case Reports* **2016**, *7*, 135–138, doi:10.1213/XAA.0000000000000375.
53. Korolev A.Yu.;Pyregov A.V.;Fedorova T.A.;Shmakov R.G.;Gerasimov Y.A.;Shpiluk M.A.;Medvedeva A.A.; Safety of Regional Anesthesia during Delivery in Pregnant Women with Placenta Increta. *Akusherstvo i Ginekologiya (Russian Federation)* **2019**, *2019*, 92–97, doi:10.18565/aig.2019.1.92-97.
54. Kuczkowski, K.M. Anesthesia for the Repeat Cesarean Section in the Parturient with Abnormal Placentation: What Does an Obstetrician Need to Know? *Archives of Gynecology and Obstetrics* **2006**, *273*, 319–321, doi:10.1007/s00404-005-0104-0.
55. Kume, K.; Tsutsumi, Y.M.; Soga, T.; Sakai, Y.; Kambe, N.; Kawanishi, R.; Hamaguchi, E.; Kawahara, T.; Kasai, A.; Nakaji, Y.; et al. A Case of Placenta Percreta with Massive Hemorrhage during Cesarean Section. *Journal of Medical Investigation* **2014**, *61*, 208–212, doi:10.2152/jmi.61.208.
56. Laranjo, M.; Aniceto, L.; Domingues, C.; Gonçalves, L.; Fonseca, J. Managing Placenta Accreta and Massive Hemorrhage: A Case Report on Anesthetic and Surgical Interventions. *Cureus Journal of Medical Science* **2024**, *16*, doi:10.7759/cureus.64071.
57. Lekic, Z.; Ahmed, E.; Peeker, R.; Sporrong, T.; Karlsson, O. Striking Decrease in Blood Loss with a Urologist-Assisted Standardized Multidisciplinary Approach in the Management of Abnormally Invasive Placenta. *Scandinavian Journal of Urology* **2017**, *51*, 491–495, doi:10.1080/21681805.2017.1352617.
58. Li, P.; Liu, X.; Li, X.; Wei, X.; Liao, J. Clinical Outcomes and Anesthetic Management of Pregnancies with Placenta Previa and Suspicion for Placenta Accreta Undergoing Intraoperative Abdominal Aortic Balloon Occlusion during Cesarean Section. *BMC Anesthesiology* **2020**, *20*, doi:10.1186/s12871-020-01040-8.
59. Lilker, S.J.; Meyer, R.A.; Downey, K.N.; MacArthur, A.J. Anesthetic Considerations for Placenta Accreta. *International Journal of Obstetric Anesthesia* **2011**, *20*, 288–292, doi:10.1016/j.ijoa.2011.06.001.

60. Liu, C.; Chu, R.; Song, N.; Yang, Q.; Song, X.; Li, L.; Zhang, M.; Li, Y.; Xu, Y.; Li, Y.; et al. Perinatal Outcomes Comparison between Neuraxial and General Anesthesia in Pregnant Women with Placenta Accreta Spectrum: A Multicenter Retrospective Study. *J. Anesth.* **2024**, *38*, 167–178, doi:10.1007/s00540-023-03287-0.
61. Lopez-Erazo, L.J.; Sánchez, B.; Blanco, L.F.; Nieto-Calvache, A.J. Placenta Accreta Spectrum Anaesthetic Management with Neuraxial Technique Can Be Facilitated by Multidisciplinary Groups. *Indian Journal of Anaesthesia* **2021**, *65*, 153–156, doi:10.4103/ija.IJA_1216_20.
62. Loreto, M.; Pisanti, M.; Celentani, M.; Pasta, G.; Erman, A.; Santangelo, C.; Giaccari, L.G.; Sansone, P.; Villani, R. Non Invasive Hemodynamic Monitoring for Fluids and Blood Resuscitation during Placenta Praevia Accreta Cesarean Delivery: A Retrospective Observational Study. *Journal of Anesthesia, Analgesia and Critical Care* **2022**, *2*, doi:https://doi.org/10.1186/s44158-022-00083-2.
63. Ma, Y.; You, Y.; Jiang, X.; Lin, X.; Chen, Y. Parallel Transverse Uterine Incisions Combined with Cell Salvage Minimized Bleeding in a Patient with Pernicious Placenta Previa and an Unexplained Decrease in Hemoglobin after Transfusion of Allogeneic Red Blood Cells: A Case Report. *Medicine (United States)* **2019**, *98*, doi:10.1097/MD.0000000000015434.
64. Ma, Y.; You, Y.; Jiang, X.; Lin, X. Use of Nitroglycerin for Parallel Transverse Uterine Cesarean Section in Patients with Pernicious Placenta Previa and Placenta Accrete and Predicted Difficult Airway: A Case Report and Review of Literature. *Medicine* **2020**, *99*, e18943, doi:10.1097/MD.0000000000018943.
65. Malik, Z.; Hartopp, R.; Shonfeld, A. The “Triple P” Procedure for the Management of Morbidly Adherent Placentas. *International Journal of Obstetric Anesthesia* **2017**, *31*, S15, doi:10.1016/j.ijoa.2017.03.004.
66. Mansouri, M.; DeStefano, K.; Monks, B.; Singh, J.; McDonnold, M.; Morgan, J.; Hale, R.; Adusumalli, J.; Horton, A.; Haeri, S. Treatment of Morbidly Adherent Placentation Utilizing a Standardized Multidisciplinary Approach in the Community Hospital-Private Practice Setting. *AJP reports* **2017**, *7*, e211–e214, doi:10.1055/s-0037-1608641.
67. Markley, J.C.; Farber, M.K.; Perlman, N.C.; Carusi, D.A. Neuraxial Anesthesia during Cesarean Delivery for Placenta Previa with Suspected Morbidly Adherent Placenta: A Retrospective Analysis. *Anesthesia and Analgesia* **2018**, *127*, 930–938, doi:10.1097/01.aoa.0000552925.04369.df.
68. Mauritz Amy A.;Dominguez Jennifer E.;Guinn Nicole R.;Gilner Jennifer;Habib Ashraf S.; Blood-Conservation Strategies in a Blood-Refusal Parturient with Placenta Previa and Placenta Percreta. *A and A Case Reports* **2016**, *6*, 111–113, doi:10.1213/XAA.0000000000000258.
69. Mok, M.; Heidemann, B.; Dundas, K.; Gillespie, I.; Clark, V. Interventional Radiology in Women with Suspected Placenta Accreta Undergoing Caesarean Section. *International Journal of Obstetric Anesthesia* **2008**, *17*, 255–261, doi:10.1016/j.ijoa.2007.11.010.
70. Morland, D.; Wight, W.; Waugh, J. Caesarean Hysterectomy in a Patient with Severe Mitral Stenosis and Placenta Accreta: A Case Report. *International Journal of Obstetric Anesthesia* **2009**, *18*, S50, doi:10.1016/j.ijoa.2009.03.006.
71. Munoz, L.; Mendoza, G.; Gomez, M.; Reyes, L.; Arevalo, J. Anesthetic Management of Placenta Accreta in a Low-Resource Setting: A Case Series. *International Journal of Obstetric Anesthesia* **2015**, *24*, 329–334, doi:10.1016/j.ijoa.2015.05.005.
72. Nagy CJ; Wheeler AS; Archer TL Acute Normovolemic Hemodilution, Intraoperative Cell Salvage and PulseCO Hemodynamic Monitoring in a Jehovah’s Witness with Placenta Percreta. *International Journal of Obstetric Anesthesia* **2008**, *17*, 159–163, doi:10.1016/j.ijoa.2007.07.005.
73. Nanji, J.A.; Ansari, J.R.; Yurashevich, M.; Ismawan, J.M.; Lyell, D.J.; Karam, A.K.; Hovsepian, D.M.; Riley, E.T. Transesophageal Echocardiographic Observation of Caval Thrombus Followed by Intraoperative Placement of Inferior Vena Cava Filter for Presumed Pulmonary Embolism during Cesarean Hysterectomy for Placenta Percreta: A Case Report. *A and A Practice* **2019**, *12*, 37–40, doi:10.1213/XAA.0000000000000836.
74. Neef, V.; Flinspach, A.N.; Eichler, K.; Woebbecke, T.R.; Noone, S.; Kloka, J.A.; Jennewein, L.; Louwen, F.; Zacharowski, K.; Raimann, F.J. Management and Outcome of Women with Placenta Accreta Spectrum and Treatment with Uterine Artery Embolization. *J Clin Med* **2024**, *13*, doi:10.3390/jcm13041062.
75. Nguyen-Lu, N.; Carvalho, J.C.A.; Kingdom, J.; Windrim, R.; Allen, L.; Balki, M. Mode of Anesthesia and Clinical Outcomes of Patients Undergoing Cesarean Delivery for Invasive Placentation: A Retrospective Cohort Study of 50 Consecutive Cases. *Canadian Journal of Anesthesia* **2016**, *63*, 1233–1244, doi:10.1007/s12630-016-0695-x.
76. Nieto, A.J.; Echavarría, M.P.; Carvajal, J.A.; Messa, A.; Burgos, J.M.; Ordoñez, C.; Benavidez, J.P.; Mejía, M.; López, L.; Fernández, P.A.; et al. Placenta Accreta: Importance of a Multidisciplinary Approach in the Colombian Hospital Setting. *Journal of Maternal-Fetal and Neonatal Medicine* **2020**, *33*, 1321–1329, doi:10.1080/14767058.2018.1517328.

77. Nieto-Calvache, A.J.; Lopez-Erazo, leydi; Sanchez, B.; Blanco-Solano, L.F.; Billefals-Vallejo, E.; Lopez-Girón, M.C.; Vergara-Galliadi, L.M.; Zambrano, M.A.; Burgos-Luna, J. 414 Multidisciplinary Management Facilitates the Application of Neuraxial Anesthesia in Placenta Accreta Spectrum. *American Journal of Obstetrics and Gynecology* **2021**, *224*, S266, doi:10.1016/j.ajog.2020.12.435.
78. Nieto-Calvache, A.J.; López-Girón, M.C.; Quintero-Santacruz, M.; Bryon, A.M.; Burgos-Luna, J.M.; Echavarría-David, M.P.; López, L.; Macia-Mejia, C.; Benavides-Calvache, J.P. A Systematic Multidisciplinary Initiative May Reduce the Need for Blood Products in Patients with Abnormally Invasive Placenta. *Journal of Maternal-Fetal and Neonatal Medicine* **2022**, *35*, 738–744, doi:10.1080/14767058.2020.1731460.
79. Ohsugi, E.; Kato, R.; Hosokawa, Y.; Oe, K. Anesthetic Management of Cesarean Hysterectomy Using Intra-Aortic Balloon Occlusion in a Patient with Fontan Circulation and Placenta Increta: A Case Report. *JA Clin Rep* **2023**, *9*, 20, doi:10.1186/s40981-023-00611-1.
80. Omowanile, Y.A.; Weiler, L.N.; Mhyre, J.M.; Khan, F.A. Double Dilemma - Management of a Pregnant Patient with a Difficult Airway Presenting with Undiagnosed Placenta Percreta: A Case Report. *A and A Case Reports* **2017**, *9*, 1–3, doi:10.1213/XAA.0000000000000508.
81. Orr, T.; McNab, R.; Roberts, F. Massive Intraoperative Cell-Salvage Transfusion in a Case of Placenta Percreta with Uterine Rupture. *International Journal of Obstetric Anesthesia* **2018**, *35*, S57.
82. Otero, C.R.; Aguilera, Z.C.B.; Expósito, R.M.; López, C.G.; Fraile, J.R.R.; Olivares, M.C.; Gómez, M.M.; Requena, M.M.G. Multidisciplinary Management of a Patient of Placenta Increta with Resuscitative Balloon Occlusion of the Aorta (REBOA) through Axillary Access. *Anesthesia and Analgesia* **2021**, *133*, 1115.
83. Oygen, Ö.; Yildirim, Z. Evaluation of 10-Year Anesthesia Management in Patients Diagnosed with Placenta Accreata Spectrum and Placenta Previa: A Comparative Study. *Medicina Balear* **2024**, *39*, doi:10.3306/AJHS.2024.39.02.32.
84. Pandey, M.; Chopra, S.; Bana, M.; Agrawal, M. Anaesthesia Management in Case of Placenta Accreta Undergoing Caesarean Delivery with Internal Iliac Artery Balloon Catheterisation and Embolisation. *Journal of Clinical and Diagnostic Research* **2024**, *18*, UD10–UD12, doi:10.7860/JCDR/2024/71424.19765.
85. Panigrahi, A.K.; Yeaton-Massey, A.; Bakhtary, S.; Andrews, J.; Lyell, D.J.; Butwick, A.J.; Goodnough, L.T. A Standardized Approach for Transfusion Medicine Support in Patients with Morbidly Adherent Placenta. *Anesthesia and Analgesia* **2017**, *125*, 603–608, doi:10.1213/ANE.0000000000002050.
86. Panjeton, G.D.; Reynolds, P.S.; Saleem, D.; Mehkri, Y.; Samra, R.; Wendling, A. Neuraxial Anesthesia and Postoperative Opioid Administration for Cesarean Delivery in Patients with Placenta Accreta Spectrum Disorder: A Retrospective Cohort Study. *International Journal of Obstetric Anesthesia* **2022**, *49*, N.PAG-N.PAG, doi:10.1016/j.ijoa.2021.103220.
87. Pareek, S.; Ajmani, T.S.; Batra, M.; Agarwal, A. A Case Report: Multidisiciplinary Approach in Successful Management of a Parturient Having Placenta Accreta Spectrum with a Standardized Massive Blood Transfusion Protocol. *Journal of Cardiovascular Disease Research* **2024**, *15*, 651–656, doi:10.48047.
88. Patil, Y.; Motghare, A.; Walavalkar, D.; Chincholi, I. Retrospective Observational Case Series of Management of Placenta Accreta at Tertiary Care Institution. *Journal of Obstetric Anaesthesia and Critical Care* **2023**, *13*, 71–74, doi:10.4103/JOACC.JOACC_4_22.
89. Parva, M.; Chamchad, D.; Keegan, J.; Gerson, A.; Horrow, J. Placenta Percreta with Invasion of the Bladder Wall: Management with a Multi-Disciplinary Approach. *Journal of Clinical Anesthesia* **2010**, *22*, 209–212, doi:10.1016/j.jclinane.2009.03.018.
90. Paull, J.D.; Smith, J.; Williams, L.; Davison, G.; Devine, T.; Holt, M. Balloon Occlusion of the Abdominal Aorta during Caesarean Hysterectomy for Placenta Percreta. *Anaesthesia and Intensive Care* **1995**, *23*, 731–734, doi:10.1177/0310057x9502300616.
91. Pini, R.; Latta, M.; Santonastaso, D.P.; Antonazzo, P.G.M.; Giacomini, G.; Bisulli, M.; Bolondi, G.; Bissoni, L.; Agnoletti, V. Management of Postpartum Hemorrhage in Urgent Cesarean Delivery for Placenta Accreta by Resuscitative Endovascular Balloon Occlusion of the Aorta (REBOA), a Case Report. *Eur J Obstet Gynecol Reprod Biol* **2023**, *286*, 152–153, doi:10.1016/j.ejogrb.2023.05.015.
92. Plakhotina, E.N.; Belousova, T.N.; Kulikov, I.A.; Latyshev, R.V.; Pavlyutina, K.M. The Choice of Anesthesia during Organ-Saving Operations Concerning Patients with Placenta Accreta Spectrum Disorders. *Sklifosovsky Journal Emergency Medical Care* **2020**, *9*, 221–230, doi:10.23934/2223-9022-2020-9-2-221-230.
93. Rana, S.; Munir, A.; Quraishi, Q.; Akhtar, A.; Pervaiz, E.; Syed, H. Anaesthetic Practices and Maternal Outcome in Rising Placenta Accreta Spectrum in Tertiary Care Hospital. *Journal of Rawalpindi Medical College* **2020**, *24*, 108–111, doi:10.37939/jrmc.v24i2.1130.
94. Riveros-Perez, E.; Wood, C. Retrospective Analysis of Obstetric and Anesthetic Management of Patients with Placenta Accreta Spectrum Disorders. *International Journal of Gynecology & Obstetrics* **2018**, *140*, 370–374, doi:10.1002/ijgo.12366.

95. Ronel, I.; Aptekman, B.; Kori, I.; Levin, I.; Ronel, R.; Greenberger, C.; Weiniger, C.F. Perioperative Outcomes of Placenta Accreta Spectrum Cesarean Delivery in a Hybrid vs Labour and Delivery Operating Room. *Canadian Journal of Anesthesia* **2023**, doi:10.1007/s12630-022-02385-z.
96. Rubab, U.I. Anesthetic Implications in Managing a Case of Placenta Percreta: A Case Report. *Anaesth. Pain Intensive Care* **2023**, *27*, 614–618, doi:10.35975/apic.v27i5.2240.
97. Russo, M.; Krenz, E.I.; Hart, S.R.; Kirsch, D.K. Multidisciplinary Approach to the Management of Placenta Accreta. *Ochsner Journal* **2011**, *11*, 84–88, doi:PMID: 21603341.
98. Sadashivaiah, J.; Wilson, R.; Thein, A.; McLure, H.; Hammond, C.J.; Lyons, G. Role of Prophylactic Uterine Artery Balloon Catheters in the Management of Women with Suspected Placenta Accreta. *International Journal of Obstetric Anesthesia* **2011**, *20*, 282–287, doi:10.1016/j.ijoa.2011.06.006.
99. Salim, R.; Chulski, A.; Romano, S.; Garmi, G.; Rudin, M.; Shalev, E. Precesarean Prophylactic Balloon Catheters for Suspected Placenta Accreta: A Randomized Controlled Trial. *Obstetrics & Gynecology* **2015**, *126*, 1022–1028, doi:10.1097/AOG.0000000000001113.
100. Schiraldi, R.; Brogly, N.; Guasch, E.; Gilsanz, F. Transoesophageal Doppler-Guided Fluid Management in Massive Obstetric Hemorrhage. *European Journal of Anaesthesiology* **2012**, *29*, 69, doi:10.1016/j.ijoa.2013.07.001.
101. Serrano, C.; Ehrig, J.C.; Hofkamp, M.P. A 29-Year-Old Woman Presenting for Urgent Cesarean Hysterectomy: A Multidisciplinary Care Challenge. *Baylor University Medical Center Proceedings* **2023**, *36*, 528–529, doi:10.1080/08998280.2023.2210795.
102. Setyawan, N.; Permana, S. Massive Transfusion in Cesarean Section Patients with Placenta Accreta: A Case Series. *Bali Journal of Anesthesiology* **2021**, *5*, 191–194, doi:10.4103/bjoa.bjoa_221_20.
103. Seyhan, T.; Sungur, M.; Edipoglu, I.; Bastu, E. Combined Spinal Epidural Anaesthesia for Caesarean Section and Hysterectomy in a Parturient with Placenta Accreta. *Turkish Journal of Anaesthesiology and Reanimation* **2014**, *42*, 148–150, doi:10.5152/TJAR.2014.59389.
104. Shamshirsaz A. Maternal Morbidity in Patients with Morbidly Adherent Placenta Treated with and without a Standardized Multidisciplinary Approach. *American Journal of Obstetrics and Gynecology* **2015**, *212*, 218.e9, doi:10.1016/j.ajog.2014.08.019.
105. Shantikaratri, E.T.; Isngadi, I.; Hartono, R. Combined Spinal Epidural Anesthesia with Hypervolemic Hemodilution Technique Showed Good Fetomaternal Outcomes in Placenta Accreta Spectrum Patients Who Underwent Elective Sectio Cesarean Surgery: A Case Series. *Regional Anesthesia & Pain Medicine* **2023**, *48*, A136.2-A137, doi:10.1136/rapm-2023-ESRA.239.
106. Silva, M.; Swinson, A.; Halpern, S.; Margarido, C. Transfusion-Alternative Strategies in a Jehovah's Witness Patient with Placenta Accreta and Severe Preeclampsia. *Canadian Journal of Anesthesia* **2011**, *58*, S87, doi:10.1007/s12630-011-9586-3.
107. Sindos, M.; Kalmantis, K.; Samartzis, K.; Diakosavvas, M.; Kalampalikis, A.; Kalopita, K.; Stamatakis, E.; Valsamidis, D.; Daskalakis, G. Peripartum Hysterectomy: A Four-Year Obstetric and Anesthetic Experience in a Tertiary Referral Hospital in Greece. *Cureus* **2022**, *14*, e25062, doi:10.7759/cureus.25062.
108. Steel, C.; Vaida, S.; Mets, B. Massive Blood Transfusion in a Patient with Immunoglobulin a Deficiency Undergoing Cesarean Delivery. *Anesthesia and Analgesia* **2010**, *110*, 1088–1090, doi:10.1213/ANE.0b013e3181d45268.
109. Styron, A.G.; George, R.B.; Allen, T.K.; Peterson-Layne, C.; Muir, H.A. Multidisciplinary Management of Placenta Percreta Complicated by Embolic Phenomena. *International Journal of Obstetric Anesthesia* **2008**, *17*, 262–266, doi:10.1016/j.ijoa.2008.03.002.
110. Sud, S.; Dwivedi, D.; Singh, S.; Gautam, A. Multidisciplinary Approach with Favorable Outcome in Management of Placenta Accreta. *Journal of Obstetric Anaesthesia and Critical Care* **2020**, *10*, 147–148, doi:10.4103/joacc.JOACC_29_19.
111. Sultan, P.; Hilton, G.; Butwick, A.; Carvalho, B. Continuous Spinal Anesthesia for Cesarean Hysterectomy and Massive Hemorrhage in a Parturient with Placenta Increta. *Can J Anaesth* **2012**, *59*, 473–477, doi:10.1007/s12630-012-9681-0.
112. Supraptomo, R.T.H. Epidural Anesthesia in a Patient with Placenta Accreta for Intra-Aortic Ballooning and Cesarean Section. *Anaesthesia, Pain and Intensive Care* **2023**, *27*, 768–771, doi:10.35975/apic.v27i6.2144.
113. Supraptomo, Rt.; Alma, H.A. Anesthesia Management in Patient with Placenta Percreta Performed Intra-Aortic Ballooning Caesarean Section. *Solo Journal of Anesthesi, Pain and Critical Care* **2021**, *1*, 15–26, doi:https://doi.org/10.20961/soja.v1i1.49481.
114. Takekawa, D.; Jinushi, K.; Kitayama, M.; Hirota, K. Rebound Hyperkalemia after Cessation of Ritodrine in a Parturient Undergoing Cesarean Section. *JA Clinical Reports* **2017**, *3*, doi:10.1186/s40981-016-0071-4.

115. Taylor, N.J.; Russell, R. Anaesthesia for Abnormally Invasive Placenta: A Single-Institution Case Series Abnormally Invasive Placenta. *International Journal of Obstetric Anesthesia* **2017**, *30*, 10–15, doi:10.1016/j.ijoa.2017.01.008.
116. Thomas M.A.F.;Krishnan P.; Abnormally Invasive Placenta Management in a District General Hospital. *International Journal of Obstetric Anesthesia* **2019**, *39*, S52.
117. Thon, S.; McLintic, A.; Wagner, Y. Prophylactic Endovascular Placement of Internal Iliac Occlusion Balloon Catheters in Parturients with Placenta Accreta: A Retrospective Case Series. *International Journal of Obstetric Anesthesia* **2011**, *20*, 64–70, doi:10.1016/j.ijoa.2010.08.006.
118. Traversa, M.; Campeggi, A.; Volpe, N.; Rocco, M. Multidisciplinary Approach Plan to Manage Challenging Case of Placenta Accreta. *International Journal of Obstetric Anesthesia* **2024**, 104101.
119. Urfalioglu, A.; Öksüz, G.; Bilal, B.; Teksen, S.; Calişir, F.; Boran, Ö.F.; Öksüz, H. Retrospective Evaluation of Anesthetic Management in Cesarean Sections of Pregnant Women with Placental Anomaly. *Anesthesiology Research and Practice* **2020**, *2020*, doi:10.1155/2020/1358258.
120. Usman, N.; Noblet, J.; Low, D.; Thangaratinam, S. Intra-Aortic Balloon Occlusion without Fluoroscopy for Severe Postpartum Haemorrhage Secondary to Placenta Percreta. *International Journal of Obstetric Anesthesia* **2014**, *23*, 91–93, doi:10.1016/j.ijoa.2013.06.006.
121. Valentine, S. Multidisciplinary Approach to Placenta Percreta: An Observational Case Study. *Journal of perianesthesia nursing : official journal of the American Society of PeriAnesthesia Nurses* **2019**, *34*, 483–490, doi:10.1016/j.jopan.2018.11.001.
122. VanMatre Melissa; Anesthesia Considerations for Placenta Accreta. *International Student Journal of Nurse Anesthesia* **2010**, *9*, 11.
123. Wei, X.; Zhang, J.; Chu, Q.; Du, Y.; Xing, N.; Xu, X.; Zhou, Y.; Zhang, W. Prophylactic Abdominal Aorta Balloon Occlusion during Caesarean Section: A Retrospective Case Series. *International Journal of Obstetric Anesthesia* **2016**, *27*, 3–8, doi:10.1016/j.ijoa.2015.12.001.
124. Weiniger, C.F.; Elram, T.; Ginosar, Y.; Mankuta, D.; Weissman, C.; Ezra, Y. Anaesthetic Management of Placenta Accreta: Use of a Pre-Operative High and Low Suspicion Classification. *Anaesthesia* **2005**, *60*, 1079–1084, doi:10.1111/j.1365-2044.2005.04369.x.
125. Wijaya, T. Total Placenta Previa with High-Risk Morbidly Adherent Placenta and Transverse Lie Fetal Position. *Bali Journal of Anesthesiology* **2021**, *5*, 201–203, doi:10.4103/bjoa.bjoa_236_20.
126. Yamada, T.; Hirahata, E.; Ihara, N.; Nishimura, D.; Inoue, K.; Kato, J.; Nagata, H.; Minamishima, S.; Morisaki, H. Cesarean Hysterectomy in a Hybrid Operating Room for Placenta Percreta: A Report of Three Cases. *JA Clinical Reports* **2019**, *5*, doi:10.1186/s40981-019-0230-5.
127. Young, H.; Ehrig, J.C.; Hammonds, K.; Hofkamp, M.P. Effect of a Placenta Accreta Spectrum Multidisciplinary Team and Checklist on Maternal Outcomes for Planned Hysterectomy at Time of Cesarean Delivery. *Baylor University Medical Center Proceedings* **2022**, *35*, 755–758, doi:10.1080/08998280.2022.2109113.
128. Zabida, A.; Zahavi, G.; Bartoszko, J.; Otálora-Esteban, M.; Weinstein, J.; Frogel, J.; Miller, L.; Sivan, E.; Orkin, D.; Dolgoker, I.; et al. Improving Blood Product Management in Placenta Accreta Patients with Severe Bleeding: Institutional Experience. *International Journal of Obstetric Anesthesia* **2023**, *56*, 103904, doi:10.1016/j.ijoa.2023.103904.
129. Zhou, F.; Liu, N.; Huang, G.; Yu, H.; Wang, X. Fluid Resuscitation Strategy in Patients with Placenta Previa Accreta: A Retrospective Study. *Front Med (Lausanne)* **2024**, *11*, 1454067, doi:10.3389/fmed.2024.1454067.
130. Zhu, H.; Wang, S.; Shi, J.; Yao, L.; Wang, L.; Chen, H.; Fang, X. Prophylactic Endovascular Balloon Occlusion of the Aorta in Cases of Placenta Accreta Spectrum during Caesarean Section: Points from the Anaesthesiologist's Perspective. *BMC Pregnancy & Childbirth* **2020**, *20*, 1–8, doi:10.1186/s12884-020-03136-y.
